# Tobacco Smoking Leads to Extensive Genome-Wide Changes in DNA Methylation

**DOI:** 10.1371/journal.pone.0063812

**Published:** 2013-05-17

**Authors:** Sonja Zeilinger, Brigitte Kühnel, Norman Klopp, Hansjörg Baurecht, Anja Kleinschmidt, Christian Gieger, Stephan Weidinger, Eva Lattka, Jerzy Adamski, Annette Peters, Konstantin Strauch, Melanie Waldenberger, Thomas Illig

**Affiliations:** 1 Research Unit of Molecular Epidemiology, Helmholtz Zentrum München, German Research Center for Environmental Health, Neuherberg, Germany; 2 Institute of Genetic Epidemiology, Helmholtz Zentrum München, German Research Center for Environmental Health, Neuherberg, Germany; 3 Hannover Unified Biobank, Hannover Medical School, Hannover, Germany; 4 Department of Dermatology, Allergology, and Venerology, University Hospital Schleswig-Holstein, Campus Kiel, Kiel, Germany; 5 Graduate School of Information Science in Health (GSISH), Technische Universität München, Munich, Germany; 6 Institute of Experimental Genetics, Genome Analysis Center, Helmholtz Zentrum München, Neuherberg, Germany; 7 Chair of Experimental Genetics, Technische Universität München, Munich, Germany; 8 Institute of Epidemiology II, Helmholtz Zentrum München, German Research Center for Environmental Health, Neuherberg, Germany; 9 Institute of Medical Informatics, Biometry and Epidemiology, Chair of Genetic Epidemiology, Ludwig-Maximilians-Universität, Munich, Germany; University of Cincinnati, United States of America

## Abstract

Environmental factors such as tobacco smoking may have long-lasting effects on DNA methylation patterns, which might lead to changes in gene expression and in a broader context to the development or progression of various diseases. We conducted an epigenome-wide association study (EWAs) comparing current, former and never smokers from 1793 participants of the population-based KORA F4 panel, with replication in 479 participants from the KORA F3 panel, carried out by the 450K BeadChip with genomic DNA obtained from whole blood. We observed wide-spread differences in the degree of site-specific methylation (with p-values ranging from 9.31E-08 to 2.54E-182) as a function of tobacco smoking in each of the 22 autosomes, with the percent of variance explained by smoking ranging from 1.31 to 41.02. Depending on cessation time and pack-years, methylation levels in former smokers were found to be close to the ones seen in never smokers. In addition, methylation-specific protein binding patterns were observed for cg05575921 within *AHRR*, which had the highest level of detectable changes in DNA methylation associated with tobacco smoking (–24.40% methylation; p = 2.54E-182), suggesting a regulatory role for gene expression. The results of our study confirm the broad effect of tobacco smoking on the human organism, but also show that quitting tobacco smoking presumably allows regaining the DNA methylation state of never smokers.

## Introduction

Epigenetic changes have been causally related to a variety of disease conditions including monogenic and complex multifactorial diseases [1]. The establishment and maintenance of epigenetic modifications, such as DNA methylation, can be modulated by environmental factors [2]–[4].

Tobacco smoking is a leading cause of disease and premature death worldwide [5]–[7]. The complex, dynamic and reactive mixture of an estimated 7,000 chemicals affects every organ system in the body and causes a wide spectrum of cardiovascular and chronic obstructive pulmonary diseases as well as various types of cancer, in particular lung cancer, through mechanisms that include DNA damage, inflammation, and oxidative stress [2], [3], [8], [9]. So far, it is insufficiently known how these mechanisms are triggered by tobacco smoking, but an association with altered DNA methylation patterns has been shown for a number of single genes, mostly cancer-related, and in genome-wide methylation studies [10]–[15]. These studies on tobacco smoking were relatively limited regarding the density of CpG site coverage and/or the number of samples analyzed. Although a few studies have already been carried out with the Illumina 27K BeadChip [10], [15], this array is limited by the fact that it only targets CpG sites located within the proximal promoter region of transcription start sites, with a focus on loci implicated in cancer. Until now, three studies concerning tobacco smoking have been accomplished with the 450K BeadChip, one using a small number of lymphoblasts and pulmonary macrophages of current and never smokers [12], another one using cord blood samples from newborns to study the effect of maternal smoking [16], and a very recent one that assessed the impact of current and former smoking on DNA methylation using whole blood samples from healthy individuals who subsequently developed breast or colon cancer and matched controls [17].

## Results and Discussion

### Illumina 450K Analysis: Genome-wide Effect of Tobacco Smoking on the Methylation Status

**Figure 1:**
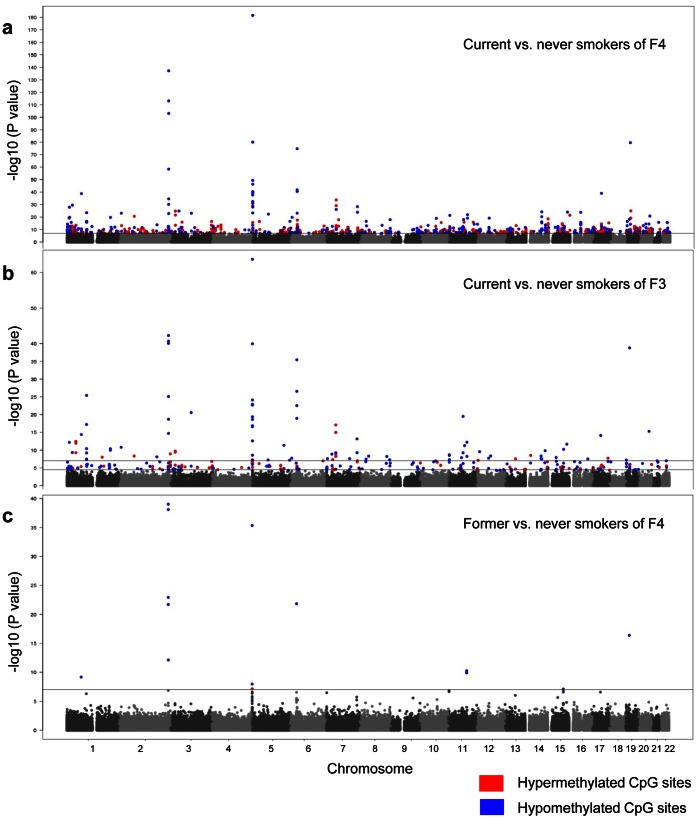
Genome-wide effect of current and former tobacco smoking on DNA methylation displayed by Manhattan Plots. The continuous lines mark the 1E-07 significance thresholds, the lower line in Figure 1b marks the 5E-05. The significant CpG sites are color coded with the direction of the aberration in current/former smokers, using blue for hypomethylated and red for hypermethylated CpG sites. a) Current vs. never smokers of the F4 discovery panel; b) Current vs. never smokers of the F3 replication panel; c) Former vs. never smokers of the F4 discovery panel.

To investigate the effect of tobacco smoking on DNA methylation, we performed a genome-wide DNA methylation analysis with the Illumina 450K BeadChip using DNA obtained from whole blood. The characteristics of the discovery (F4) and the replication (F3) panel are summarized in Table 1. Visual presentation of the genome-wide distribution of the significant, differentially-methylated CpG sites of current vs. never smokers in the discovery (F4; current N = 262, never N = 749) and replication (F3; current N = 236, never N = 232) panel are represented as Manhattan Plots in Figure 1a and 1b respectively.

**Table 1:** Characteristics of the study populations.

	Discovery panel: F4		p-value Never-		p-value Never-	Replication panel: F3			p-value Never-
	Never smokers	Former smokers	Former*	Current smokers	Current*	Never smokers	Current smokers		Current*
N	749 (41.77%)	782 (43.61%)		262 (14.61%)		232 (49.57%)	236 (50.43%)		
Age (years)	62.09 (31–81)	61.02 (35–81)	0.01^a^	56.96 (46–76)	<0.001^a^	53.16 (34–83)	52.78 (35–80)		ns ^a^
Gender			<0.001^b^		<0.001^b^				ns ^b^
Female	490 (65.42%)	310 (39.64%)		120 (45.80%)		115 (49.57%)	116 (49.15%)		
Male	259 (34.58%)	472 (60.36%)		142 (54.20%)		117 (50.43%)	120 (50.85%)		
Smoking									
Pack-years	0	23.00 (0.60–195)		33.64 (0.54–127)					
Time since quitting (years)		21.83 (0.08–62.5)							
BMI (kg/m^2^)	28.05 (18.99–45.17)	28.61 (17.50–55.99)	ns ^a^	27.11 (18.68–47.31)	<0.001^a^	27.31 (15.07–45.31)	26.74 (17.50–45.28)		ns ^a^
WBC count (WBC/nl)	5.64 (2.80–14.50)	5.75 (2.70–13.00)	ns ^a^	7.08 (2.90–17.90)	<0.001^a^	6.61 (3.40–29.10)	8.06 (3.90–15.10)		0.02^a^
Alcohol (g/kg/day)	0.15 (0.00–1.38)	0.22 (0.00–1.42)	<0.001^a^	0.23 (0.00–1.55)	0.005^a^	0.17 (0.00–0.95)	0.23 (0.00–1.21)		<0.001^a^

BMI: Body Mass Index, WBC: White Blood Cells; each individual characteristic is given in mean with range in parentheses. * Group comparison - ns: not significant according to ^a^ paired Wilcoxon rank sum test with never smokers as reference and ^b^ Fisher’s exact test for gender.

Depending upon the smoking status, we identified 972 CpG sites with differential methylation levels after conservative correction for multiple testing (p≤1E-07) throughout the genome in F4 (Table S1), of which 187 CpG sites could be replicated in F3 (p≤5E-5; Table S2). Table 2 displays all replicated CpG sites of current vs. never smokers with a methylation difference of more than 5% in both panels. In addition, a meta-analysis of the F4 and F3 data sets was performed, displayed by the corresponding p-value in Table 2 and Table S2.

**Table 2:** Genome-wide significant differentially-methylated CpG sites of current compared to never smokers.

			Discovery panel (F4)			Replication panel (F3)			Meta-analysis	Methylation ß-value as median (first quartile - third quartile)			
										Discovery panel (F4)		Replication panel (F3)	
CpG	Chr	Gene	Median ß-value methylation difference in %	p-value	Explained variance in %	Median ß-value methylation difference in %	p-value	Explained variance in %	p-value	Never smokers	Current smokers	Never smokers	Current smokers
cg04885881	1	x^a^	–7.41	1.35E-28	9.57	–6.71	6.62E-13	9.83	7.19E-40	0.3115 (0.25–0.39)	0.2374 (0.18–0.31)	0.2899 (0.24–0.36)	0.2228 (0.17–0.29)
cg15542713	1	HIVEP3	9.52	2.54E-08	2.03	9.95	3.65E-13	9.51	3.56E-18	0.4002 (0.31–0.50)	0.4954 (0.38–0.60)	0.3784 (0.30–0.46)	0.4779 (0.40–0.57)
cg25189904	1	GNG12	–8.19	1.71E-39	14.27	–7.40	4.13E-15	11.42	9.11E-53	0.2740 (0.23–0.33)	0.1921 (0.16–0.24)	0.2856 (0.24–0.33)	0.2116 (0.17–0.26)
cg12876356	1	GFI1	–8.48	2.92E-17	5.46	–22.63	6.18E-18	13.59	2.47E-32	0.5475 (0.49–0.62)	0.4627 (0.37–0.55)	0.7010 (0.56–0.80)	0.4746 (0.33–0.60)
cg09935388	1	GFI1	–15.31	3.27E-24	7.39	–16.47	3.98E-26	18.93	1.26E-46	0.5765 (0.49–0.70)	0.4234 (0.32–0.54)	0.4757 (0.40–0.54)	0.3110 (0.24–0.39)
cg27241845	2	x^a^	–8.74	2.79E-35	10.96	–8.36	2.07E-19	13.78	6.03E-53	0.4617 (0.41–0.52)	0.3743 (0.31–0.42)	0.4457 (0.41–0.49)	0.3621 (0.31–0.43)
cg21566642	2	ALPPL2^b^	–16.70	6.90E-138	36.24	–15.58	8.82E-41	27.13	7.86E-175	0.5658 (0.51–0.61)	0.3988 (0.35–0.45)	0.5511 (0.50–0.59)	0.3953 (0.31–0.44)
cg01940273	2	ALPPL2^b^	–7.89	9.28E-114	31.50	–7.53	5.46E-43	28.33	1.92E-154	0.3437 (0.32–0.37)	0.2648 (0.24–0.29)	0.2941 (0.27–0.31)	0.2189 (0.20–0.25)
cg15693572	3	x^a^	10.87	3.84E-14	4.50	6.90	1.74E-06	4.60	3.69E-19	0.2942 (0.22–0.41)	0.4029 (0.29–0.53)	0.3159 (0.23–0.40)	0.3850 (0.29–0.50)
cg23480021	3	x^a^	18.63	2.67E-22	8.74	15.77	1.95E-10	8.09	3.63E-31	0.4259 (0.30–0.60)	0.6122 (0.44–0.78)	0.4266 (0.29–0.58)	0.5844 (0.43–0.78)
cg03274391	3	x^a^	18.06	1.67E-25	8.79	11.93	3.74E-10	7.78	5.47E-34	0.3190 (0.23–0.47)	0.4997 (0.34–0.70)	0.2872 (0.21–0.41)	0.4065 (0.29–0.55)
cg15417641	3	CACNA1D	7.92	1.25E-16	4.90	5.28	1.90E-06	4.61	1.65E-21	0.6818 (0.58–0.76)	0.7610 (0.68–0.83)	0.6019 (0.52–0.68)	0.6546 (0.60–0.73)
cg21188533	3	CACNA1D	10.18	1.12E-10	3.10	5.56	4.38E-05	3.42	2.33E-14	0.5724 (0.45–0.69)	0.6742 (0.55–0.76)	0.5309 (0.41–0.63)	0.5865 (0.50–0.70)
cg03991871	5	AHRR	–5.73	4.84E-41	12.72	–7.88	1.23E-23	17.61	8.03E-63	0.9469 (0.92–0.96)	0.8896 (0.80–0.93)	0.9175 (0.89–0.94)	0.8387 (0.69–0.89)
cg05575921	5	AHRR	–24.40	2.54E-182	41.02	–23.29	1.81E-64	39.69	8.70E-244	0.8841 (0.86–0.90)	0.6401 (0.60–0.73)	0.9028 (0.88–0.92)	0.6699 (0.58–0.77)
cg26703534	5	AHRR	–6.17	2.14E-38	10.90	–5.77	7.83E-25	17.21	3.66E-61	0.6269 (0.60–0.65)	0.5652 (0.52–0.60)	0.6180 (0.60–0.63)	0.5603 (0.53–0.60)
cg25648203	5	AHRR	–7.96	4.73E-47	12.98	–7.55	2.03E-23	16.96	1.01E-68	0.8642 (0.83–0.89)	0.7846 (0.72–0.84)	0.8473 (0.81–0.87)	0.7719 (0.71–0.82)
cg21161138	5	AHRR	–10.45	8.58E-81	21.63	–10.60	1.19E-40	28.49	1.49E-119	0.6998 (0.67–0.73)	0.5954 (0.53–0.65)	0.7304 (0.70–0.75)	0.6244 (0.57–0.68)
cg06126421	6	x^a^	–17.05	1.72E-75	23.60	–17.89	3.73E-36	24.58	8.42E-110	0.7724 (0.70–0.82)	0.6019 (0.48–0.69)	0.6919 (0.63–0.74)	0.5131 (0.42–0.61)
cg14753356	6	x^a^	–5.37	8.14E-24	8.58	–6.41	2.98E-23	17.61	5.90E-44	0.2383 (0.20–0.28)	0.1846 (0.15–0.22)	0.2451 (0.20–0.28)	0.1810 (0.15–0.22)
cg00931843	6	TIAM2	6.37	1.69E-10	2.98	5.66	1.09E-07	5.73	1.37E-16	0.2963 (0.23–0.39)	0.3600 (0.27–0.47)	0.3293 (0.25–0.42)	0.3859 (0.29–0.46)
cg22132788	7	MYO1G	6.68	1.99E-34	11.04	5.02	8.44E-18	13.68	1.54E-50	0.8599 (0.81–0.90)	0.9267 (0.88–0.95)	0.8727 (0.83–0.90)	0.9229 (0.88–0.95)
cg12803068	7	MYO1G	14.96	7.08E-30	10.34	15.12	1.04E-15	12.29	6.33E-44	0.7382 (0.60–0.85)	0.8878 (0.77–0.94)	0.7014 (0.60–0.81)	0.8527 (0.71–0.92)
cg11207515	7	CNTNAP2	8.28	2.53E-10	3.44	5.29	2.16E-07	5.30	3.77E-16	0.2725 (0.21–0.35)	0.3552 (0.27–0.44)	0.2166 (0.17–0.27)	0.2695 (0.21–0.33)
cg25305703	8	x^a^	–6.05	2.09E-08	2.97	–6.17	6.16E-09	6.16	2.75E-15	0.7103 (0.65–0.76)	0.6498 (0.58–0.73)	0.6679 (0.62–0.71)	0.6062 (0.54–0.67)
cg26361535	8	ZC3H3	–5.54	4.14E-09	2.83	–6.35	4.39E-07	4.90	1.35E-14	0.7593 (0.70–0.81)	0.7040 (0.63–0.77)	0.7442 (0.69–0.79)	0.6807 (0.64–0.75)
cg21611682	11	LRP5	–5.24	1.09E-18	6.50	–5.38	3.16E-20	14.13	9.90E-36	0.5276 (0.50–0.56)	0.4752 (0.43–0.52)	0.4844 (0.46–0.51)	0.4306 (0.40–0.47)
cg23126342	13	PCDH9	9.11	7.16E-14	4.38	7.56	2.73E-08	6.20	1.23E-20	0.5136 (0.43–0.60)	0.6047 (0.52–0.67)	0.4865 (0.39–0.57)	0.5621 (0.46–0.64)
cg01208318	14	x^a^	–7.74	2.54E-12	4.15	–6.20	1.20E-05	3.59	1.57E-16	0.2836 (0.20–0.37)	0.2063 (0.16–0.28)	0.2246 (0.15–0.30)	0.1626 (0.12–0.23)
cg19572487	17	RARA	–10.02	9.37E-40	14.02	–7.81	7.56E-15	11.92	9.85E-53	0.4477 (0.40–0.51)	0.3475 (0.30–0.43)	0.4502 (0.40–0.51)	0.3722 (0.31–0.44)
cg00835193	19	LINGO3	–8.23	2.44E-08	2.26	–11.66	4.40E-07	5.01	9.20E-14	0.8822 (0.75–0.93)	0.7999 (0.61–0.92)	0.8938 (0.77–0.94)	0.7772 (0.59–0.91)
cg03636183	19	F2RL3	–14.74	2.42E-80	22.45	–17.63	1.65E-39	26.94	5.58E-118	0.4930 (0.45–0.54)	0.3456 (0.28–0.43)	0.5152 (0.47–0.56)	0.3390 (0.28–0.43)

Displayed are the results of the linear model calculated with M-value adjusted for age, sex, BMI, alcohol and white blood cell count (p-value and explained variance), as well as the median ß-value methylation difference between current and never smokers for the discovery panel (F4) with genome-wide significance (p-value ≤1E-07) for all CpG sites with a DNA methylation difference of >5% in F4 and F3, the corresponding results of the same CpG sites for the replication panel F3 for comparison (significance level ≤5E-05) and the corresponding p-value gained by meta-analysis of F4 and F3, sorted by chromosome and mapinfo (Genome build 37). ^a^ According to UCSC Genome Browser no annotated transcripts are associated with these CpG sites; ^b^ According to UCSC Genome Browser no annotated transcripts are associated with these CpG sites, but SNPs within the same region (shore of a CpG Island) have a predicted function on the *ALPPL2* gene, which is located several kb apart from this CpG island.

Overall, genome-wide significant differentially-methylated CpG sites could be detected in each of the 22 autosomes with p-values ranging from 9.31E-08 to 2.54E-182, and with a percent of variance explained by smoking of 1.31 to 41.02 (Table S2). Among the CpG sites showing DNA methylation differences of more than 5%, a remarkable clustering of smoking dependent changes in methylation patterns could be identified on chromosome 2q37.1 and 5p15.3 (Figure 1a and Table 2).

The most striking and significant CpG site, cg05575921 (current smokers; F4: –24.40%, p = 2.54E-182, explained variance = 41.02%; F3: –23.29%, p = 1.81E-64, explained variance = 39.69%), is located in the region of chromosome 5p15.3 within the *AHRR* gene (Table 2 and Figure S1a). The human *AHRR* (aryl hydrocarbon receptor (*AHR*) repressor) codes for an evolutionary conserved bHLH-PAS (basic helix-loop-helix/Per-AHR nuclear translocator (ARNT)-Sim) protein. This protein is part of the aryl hydrocarbon receptor *(*AHR) signaling cascade, which mediates dioxin toxicity, is involved in regulation of cell growth and differentiation [18], [19] and the modulation of the immune system [20]. Furthermore, evidence exists for AHR crosstalk with estrogen receptor (ER) signaling, thereby impacting cell proliferation and metabolism by P450 enzymes [21]. An overview of the *AHRR* gene structure is given in Figure S1a.

Tobacco smoke is a remarkable source of polycyclic aromatic hydrocarbons (PAHs) that trigger the AHR signaling pathway [22]–[24], leading to several pathological effects in humans through AHR-dependent changes in gene expression [25]–[28]. AHRR is a known tumor suppressor, mediating detoxification of PAHs, which are the principle carcinogenic agents causing tobacco-related lung and other cancers [29]. Recently a differential methylation of CpG sites in smokers within the *AHRR* gene has been demonstrated in lymphoblasts and pulmonary macrophages by Monick et al. [12]. Our findings are also in line within another recent study of Joubert et al. carried out in cord blood of newborns in order to analyze epigenome-wide methylation in relation to maternal smoking during pregnancy. This study also found cg05575921 in *AHRR* to be the most statistically significant CpG site and showed that lower methylation was associated with higher levels of maternal smoking [16]. Furthermore, *AHRR* was also found to be differentially-methylated in the very recent study of Shenker et al. carried out in whole blood [17].

The second most striking region on chromosome 2q37.1 comprises 13 smoking-dependent, differentially-methylated CpG sites that could be detected in F4, of which 10 could be replicated in F3 (Table 2, Table S1, Table S2 and Figure S1b). Three closely related alkaline phosphatase genes, placental (*ALPP*), placental-like (*ALPPL2*) and intestinal (*ALPI*) are located within this region. Five of the detected CpG sites, including the second most outstanding CpG site respective to significance and level of detectable changes in DNA methylation patterns associated with tobacco smoking (cg21566642; F4: –16.70%, p = 6.90E-138, explained variance = 36.24%; F3: –15.58%; p = 8.82E-41, explained variance = 27.13%), were located within or in the shore of a CpG island (CGI) 9kb apart from the 3′UTR of the *ALPPL2* gene. Even though this CGI is far apart from the *ALPPL2* gene, SNPs within this CGI are predicted to have a functional impact on the *ALPPL2* gene (http://genome.ucsc.edu/). CpG sites in this region were also found to be differentially-methylated in pulmonary macrophages within the study of Monick et al. [12] and in whole blood within the study of Shenker [17]. The same group further showed an association of cg01940273 (F4: –7.89%, p = 9.28E-114, explained variance = 31.50%; F3: –7.53%; p = 5.46E-43, explained variance = 28.33%) with developing breast cancer [17].

Alkaline phosphatases (ALPs) are responsible for the dephosphorylation of various molecules such as proteins, nucleotides or alkaloids. Quantitative variations of circulating alkaline phosphatase concentrations are associated with premature birth [30], [31], low birth weight [32], [33] and pre-eclampsia [34]. Serum ALPP and ALPPL2 enzyme levels are increased up to 10-fold in 80% of cigarette smokers [35]–[37] and were shown to be elevated in patients with a number of cancers, especially seminoma [38], [39].

An additional 25 CpG sites showed DNA methylation differences of more than 5% (Table 2), located in the genes *HIVEP3, GNG12, GFI1, CACNA1D, TIAM2, MYO1G*, *CNTNAP2, ZC3H3, LRP5, PCDH9, RARA, LINGO3* and *F2RL3*, or in regions with no annotated transcripts (for detailed information, see Box S1). Previous studies have already reported a significant association of tobacco smoking with CpG site cg03636183, located within the *F2RL3* gene (current smokers; F4: –14.74%, p = 2.42E-80; F3: –17.63%, p = 1.65E-39) [10], [12], [15], [17]. The F2RL3 protein is relevant for cardiovascular physiology and plays a role in platelet activation [40] and cell signaling [4]. Breitling and co-workers reported an association of *F2RL3* methylation with mortality among patients with stable coronary heart disease [41]. Furthermore, we were able to replicate an association at the *GPR15* locus, which showed relative hypomethylation in current smokers in two recent studies using the Illumina 27K BeadChip (cg19859270; current smokers; F4: –1.31%, p = 9.00E-24; F3: –1.94%, p = 2.79E-21) (Table S2) [10], [15]. We could replicate the association at the intergenic region at 6p21.33, that has recently been demonstrated by Shenker et al. (cg06126421; current smokers; F4: –17.05%, p = 1.72E-75; F3: –17.89%, p = 3.73E-36) [17], and were moreover able to detect three additional sites within this region (cg14753356, cg24859433, cg15342087) (Table S2). Replication of sites found within Shenker and co-workers, accompanied by additional findings for the corresponding regions, could also be achieved for the genes *GNG12* (cg25189904), *GFI1* (cg09935388), *CNTNAP2* (cg25949550) and *LRP5* (cg21611682) (please see Table S2 for additional sites found within these genes) [17]. *GFI1* (cg09662411 and cg09935388), *MYOIG* (cg22132788 and cg04180046) and *CNTNAP2* (cg25949550) could also be identified at genome–wide significance in relation to maternal smoking by the study of Joubert et al. [16].

In order to test for gender-specific effects of tobacco smoking on differential DNA methylation, an interaction model was analyzed with the use of the discovery panel (F4), where the smoking status * sex interaction was included in the main model. No significant CpG sites were detected for the interaction term, suggesting no difference between males and females in methylation change due to smoking. Nonetheless, female and male subjects were analyzed separately with the use of the discovery panel (F4), with additional adjustment for pack-years as well as the previously mentioned covariates, as men and women showed a considerable difference in this variable (p<0.001). In males 42 CpG sites were found to be significant differentially-methylated in current compared to never smokers. Compared with the general analysis that included both sexes, 35 of these sites have been replicated in F3, 5 were only significant in F4 but not F3, and two sites were found to be only significant in the separate male analysis (cg05498905 and cg00395697). In females only 10 CpG sites were found to be significant differentially-methylated in current compared to never smokers, all than one (cg12806681; p = 2.00E-05 in males) were also present within the significant sites of the male analysis and replicated in F3 (Table S3). Overall, the difference in DNA methylation between current and never smokers was found to be only slightly more pronounced in males than in females. Most CpG sites detected in the model for men, in addition to the nine overlapping CpG sites, were close to the genome-wide significance level also observed in the female model, which explains why no significant CpG sites were detected for the interaction term.

### Sequenom EpiTYPER Analysis: Technical Validation of the 450K Results

The differential methylation for the three most significant loci (*AHRR* - cg05575921, *ALPP/ALPPL2* - cg21566642 and *F2RL3* - cg03636183), was validated via Sequenom’s EpiTYPER approach on 20 randomly selected current and never smokers of the KORA F4 panel. The characteristics are summarized in Table S4 and association results, covering several CpG sites within these regions, are displayed in Table S5. Two of the three CpG sites could not be directly covered by the EpiTYPER assay, owing to low mass (cg03636183, <1.500 Da) and high mass (cg21566642, >7.000 Da) of the cleavage product, thus lying outside the analytical window of the mass spectrometry. However, both target and their flanking CpG sites are located in or on the shore of a CGI, and distribution of DNA methylation within a definite genomic element as a CGI is known to be relatively homogeneous. This uniformity leads to similar levels of DNA methylation and therefore allows the representative analysis of CpG sites neighboring the actual target CpG site [42]. The top CpG site (cg05575921) was validated directly. Within the three regions assayed in the EpiTYPER analysis, only one additional CpG site, CpG_7 of the *AHRR* loci, corresponded to another 450K CpG site, cg23576855. This CpG site was also significantly associated with current smoking in our analysis, but had to be excluded as it did not show normally distributed residuals (please see method section for more information).

The association with smoking status of the loci from the 450K experiment could be technically validated with this technique (significant after Bonferroni p≤0.05/28 = 0.0018), demonstrating the reliability of the array in general.

### Genome-wide Effect of Former Tobacco Smoking on the Methylation Status

To investigate if the changes in DNA methylation remain after quitting tobacco smoking, we analyzed the DNA methylation level of former smokers compared to never smokers in the F4 panel (former N = 782, never N = 749; see Table 1 for characteristics of the study populations). The results are shown in Figure 1c. In former smokers, the methylation levels of most CpG sites, which were differentially-methylated in current vs. never smokers, were almost comparable to the state found in never smokers. However, 13 of the 187 replicated CpG sites showed significantly lower methylation levels in former smokers compared to never smokers, although differences were less pronounced (Table 3). Except for cg03604011, all of the significant CpG sites in former smokers were hypomethylated compared to never smokers (Figure 1c and Table 3).

**Table 3:** Significant differentially-methylated CpG sites of former compared to never smokers (KORA F4).

			Former smokers			Current smokers			Methylation ß-value as median (first quartile - third quartile)		
CpG	Chr.	Gene	Median ß-value methylation difference in %	p-value	Explained variance in %	Median ß-value methylation difference in %	p-value	Explained variance in %	Never smokers	Former smokers	Current smokers
cg25189904	1	GNG12	−2.36%	6.85E–10	2.54	−8.19%	1.71E–39	14.27	0.2740 (0.23–0.33)	0.2504 (0.21–0.31)	0.1921 (0.16–0.24)
cg03329539	2	ALPPL2^b^	–0.61%	7.58E-13	3.26	–2.39%	3.66E-59	19.04	0.1598 (0.15–0.17)	0.1537 (0.14–0.17)	0.1359 (0.13–0.15)
cg06644428	2	ALPPL2^b^	–3.09%	1.20E-23	6.20	–7.05%	6.37E-31	12.14	0.2142 (0.17–0.27)	0.1833 (0.14–0.23)	0.1437 (0.11–0.19)
cg05951221	2	ALPPL2^b^	–1.87%	8.18E-39	9.77	–5.21%	8.92E-104	31.07	0.2199 (0.20–0.24)	0.2012 (0.18–0.22)	0.1678 (0.15–0.19)
cg21566642	2	ALPPL2^b^	–4.31%	9.49E-40	10.22	–16.70%	6.90E-138	36.24	0.5658 (0.51–0.61)	0.5227 (0.46–0.57)	0.3988 (0.35–0.45)
cg01940273	2	ALPPL2^b^	–2.26%	2.01E-22	5.56	–7.89%	9.28E-114	31.50	0.3437 (0.32–0.37)	0.3211 (0.30–0.35)	0.2648 (0.24–0.29)
cg11554391	5	AHRR	–0.15%	1.10E-08	2.28	–1.03%	5.07E-24	7.71	0.0911 (0.08–0.10)	0.0895 (0.08–0.10)	0.0808 (0.07–0.09)
cg05575921	5	AHRR	–3.31%	4.62E-36	9.25	–24.40%	2.54E-182	41.02	0.8841 (0.86–0.90)	0.8510 (0.79–0.89)	0.6401 (0.60–0.73)
cg03604011	5	AHRR	1.58%	7.21E–08	1.84	3.40%	7.87E–30	9.49	0.0759 (0.06–0.10)	0.0917 (0.07–0.12)	0.1099 (0.08–0.15)
cg06126421	6	x^a^	–5.65%	1.50E-22	5.34	–17.05%	1.72E-75	23.60	0.7724 (0.70–0.82)	0.7159 (0.63–0.79)	0.6019 (0.48–0.69)
cg11660018	11	PRSS23	–1.67%	1.28E-10	2.46	–3.87%	1.29E-22	6.88	0.2926 (0.27–0.32)	0.2759 (0.25–0.30)	0.2538 (0.23–0.28)
cg23771366	11	PRSS23	–0.98%	5.55E-11	2.93	–2.26%	7.62E-20	7.05	0.1993 (0.18–0.22)	0.1895 (0.17–0.21)	0.1767 (0.16–0.19)
cg03636183	19	F2RL3	–3.48%	4.21E-17	4.75	–14.74%	2.42E-80	22.45	0.4930 (0.45–0.54)	0.4582 (0.40–0.51)	0.3456 (0.28–0.43)

Displayed are the results of the linear model calculated with M-value adjusted for age, sex, BMI, alcohol and white blood cell count (p-value and explained variance), as well as the median ß-value methylation difference between former and never smokers, for former smokers of F4 with genome-wide significance (p-value ≤1E-07) and the corresponding results of the same CpG sites for current smokers; sorted by chromosome and mapinfo (Genome build 37).**^a^** According to UCSC Genome Browser no annotated transcripts are associated with these CpG sites; **^b^** According to UCSC Genome Browser no annotated transcripts are associated with these CpG sites, but SNPs within the same region (shore of a CpG Island) have a predicted function on the *ALPPL2* gene, which is located several kb apart from this CpG island.

### The Effect of Cessation Time and Cumulative Smoke Exposure (Pack-years) on DNA Methylation in Former Smokers

**Figure 2:**
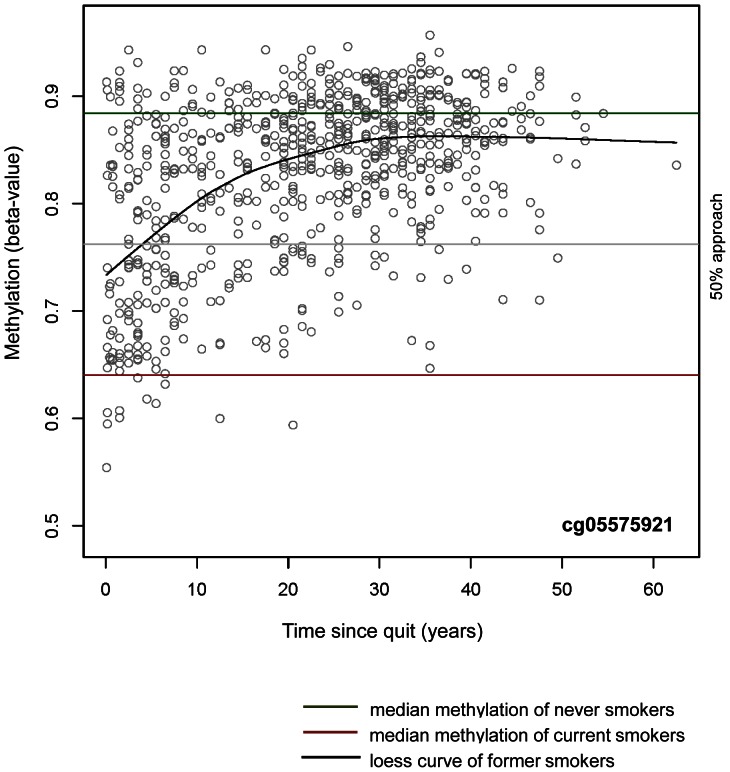
Influence of cessation time on the DNA methylation state of cg05575921. The years required for former smokers to obtain a median ß-value methylation state at CpG site cg05575921 that is closer to or equals the one of never smokers is illustrated by a loess curve in the scatterplot; the x-axis displays the cessation time in years, the y-axis displays the methylation level with the use of numbers between 0 (for 0% methylation) and 1 (for 100% methylation); horizontal brown line: median methylation level of current smokers; horizontal green line: median methylation level of never smokers; horizontal grey line: center line of current and never smokers median ß-value methylation; please see Table 4 for detailed data.

The time course over which DNA methylation is subject to change is not known, but it is assumed that it occurs in a CpG site-specific manner. Therefore, we assessed the linear effect of time after quitting smoking, on the degree of DNA methylation in former smokers of the F4 panel. This was found to be significant in 36 of the 187 CpG sites (p = 8.44E-08–7.73E-44, explained variance = 3.15–21.48%; Table 4). To get an impression of the time period that may be needed for former smokers to achieve the median ß-value methylation state of never smokers, a smooth curve was plotted in the scatter plot. Years needed for former smokers to gain a median ß-value methylation state that is closer to or equals the one of never smokers are visualized by scatter plots (Figure S2). While in the majority of cases a relatively fast approach to the level of never smokers could be detected in former smokers who have quit recently, this seemed to slow down substantially depending on how many years, or decades ago, a person quit smoking. Interestingly, the degree of methylation difference between current vs. never smokers did not seem to have an impact on how close former smokers could come to the state of never smokers. For example, cg05575921 within *AHRR*, which exhibited the highest difference in median ß-value methylation (current smokers; –21.09%), showed a relatively fast approach to the methylation level of never smokers within the first years of quitting. This approach seemed to stagnate after a few decades, as the median ß-value methylation level of former smokers never completely approached the level of never smokers (Figure 2). The study of Wan et al., carried out with the 27K BeadChip, was able to detect three sites that were differentially methylated according to time since quitting. We were able to replicate the site within the *F2RL3* gene (cg03636183), but could not confirm the other two sites in the genes *GPR15* and *LRRN3* [15]. A recent large-scale whole-genome gene expression study by Bosse et al., carried out on non-tumor lung tissue from patients with lung cancer, showed that the expression of most genes with altered smoking-dependent expression reverted to the levels of never smokers, but some genes also showed very slow or no reversibility in expression. Moreover, within this study *AHRR* was found to be the most significant probe set between never and current smokers with a fold change of 6.1. Upon smoking cessation, the expression of this gene fell extensively, but changes slowed down substantially in later years, never reaching the level of never smokers, which corresponds to the progress of DNA methylation changes we were able to detect within this gene (Figure 2 and Table 4) [43].

**Table 4:** The effect of cessation time on DNA methylation.

CpG	Chr.	Gene	Median methylation ß-value in % Current smokers	Median methylation ß-value in % Neversmokers	Median methylation ß-value in % Former smokers	p-value	Explained variance in %
cg25189904	1	GNG12	19.21	27.40	25.04	5.19E-13	6.80
cg23079012	2	x^a^	94.84	95.94	95.80	2.48E-14	7.49
cg03329539	2	ALPPL2^b^	13.59	15.98	15.37	1.92E-14	7.22
cg06644428	2	ALPPL2^b^	14.37	21.42	18.33	5.71E-12	5.80
cg05951221	2	ALPPL2^b^	16.78	21.99	20.12	1.06E-29	14.92
cg21566642	2	ALPPL2^b^	39.88	56.58	52.27	7.26E-34	16.75
cg01940273	2	ALPPL2^b^	26.48	34.37	32.11	5.94E-22	11.29
cg15693572	3	x^a^	40.29	29.42	32.53	4.84E-09	4.45
cg03274391	3	x^a^	49.97	31.90	37.32	2.15E-08	4.10
cg11554391	5	AHRR	8.08	9.11	8.95	1.91E-09	4.51
cg17924476	5	AHRR	32.08	26.28	27.95	6.03E-08	3.83
cg03991871	5	AHRR	88.96	94.69	93.59	4.14E-12	6.16
cg11902777	5	AHRR	2.67	3.79	3.37	3.97E-11	5.71
cg05575921	5	AHRR	64.01	88.41	85.10	7.73E-44	21.48
cg14817490	5	AHRR	9.68	13.50	13.33	2.79E-08	3.96
cg21161138	5	AHRR	59.54	69.98	68.05	9.81E-15	7.57
cg03604011	5	AHRR	10.99	7.59	9.17	2.29E-09	4.58
cg06126421	6	x^a^	60.19	77.24	71.59	3.84E-24	11.03
cg14753356	6	x^a^	18.46	23.83	21.57	4.60E-08	3.28
cg24859433	6	x^a^	89.19	93.22	92.46	8.17E-10	4.55
cg15342087	6	x^a^	90.36	93.71	93.16	7.48E-08	3.45
cg23565821	6	CUTA	14.55	14.72	15.09	6.29E-08	3.15
cg12803068	7	MYO1G	88.78	73.82	76.30	7.47E-11	5.51
cg21322436	7	CNTNAP2	23.81	28.55	27.95	1.35E-08	3.94
cg25949550	7	CNTNAP2	7.14	8.52	8.31	2.31E-09	4.57
cg24540678	8	x^a^	7.87	8.55	8.36	3.79E-10	4.86
cg11660018	11	PRSS23	25.38	29.26	27.59	7.45E-10	4.54
cg23771366	11	PRSS23	17.67	19.93	18.95	3.56E-13	6.91
cg09084200	11	VPS26B/NCAPD3	14.32	14.85	14.93	8.44E-08	3.22
cg02583484	12	HNRNPA1	12.67	14.76	14.22	3.56E-08	3.81
cg23161492	15	ANPEP	10.53	12.12	11.75	4.82E-08	3.84
cg19572487	17	RARA	34.75	44.77	42.73	1.71E-08	4.20
cg03636183	19	F2RL3	34.56	49.30	45.82	1.73E-32	16.88
cg12303084	20	ZMYND8	9.28	9.91	10.00	2.33E-08	3.93
cg00871610	21	MIR802	48.34	52.67	50.85	2.18E-12	6.01
cg01127300	22	x^a^	41.93	45.76	44.69	1.15E-08	4.02

The results of the linear model for time since quitting are displayed, with genome-wide significance level p≤1E-07, calculated with M-value and adjusted for age, sex, BMI, alcohol and white blood cell count (p-value and explained variance), including former smokers of F4 only, as well as the median ß-value methylation levels for current, never and former smokers; CpG sites are sorted by chromosome and mapinfo (Genome build 37); ^a^ According to UCSC Genome Browser no annotated transcripts are associated with these CpG sites; ^b^ According to UCSC Genome Browser no annotated transcripts are associated with these CpG sites, but SNPs within the same region (shore of a CpG Island) have a predicted function on the *ALPPL2* gene, which is located several kb apart from this CpG island.

However, dynamic changes in DNA methylation in former smokers not only occurred in response to cessation time, but also in response to cumulative smoke exposure (pack-years), and were found to be significant in 14 CpG sites. All 14 CpG sites were also significant in time since quitting and replicated in F3 (Table S6). The number of pack-years needed for former smokers to reach a median ß-value methylation state that is closer to or equals the one of current smokers are visualized by scatterplots in Figure S3.

To analyze the combined effect of cessation time and cumulative smoke exposure, we calculated another model that included both ‘time since quitting’ and ‘pack-years’. This approach showed that the combination of these two variates had an influence on the DNA methylation state of former smokers (Table S7). Moreover, by the use of an interaction model, two CpG sites showed genome-wide significance between time since quitting and pack-years (cg24128853, p = 2.80E-08, effect of interaction = 0.00029; cg24504601, p = 7.44E-08, effect of interaction = 0.00040). However, these two CpG sites were neither in the 36 that were found to have a significant linear effect of time after quitting smoking on the degree of DNA methylation in the former smokers nor in the combined model. Furthermore, these two sites were not found in the general smoking model. Overall, the methylation levels of subjects with the longest cessation time and the lowest cumulative smoke exposure were closest to the levels observed in never smokers (data not shown), which is in line with the results of a recent study [15].

### Functional Analysis by Electrophoretic Mobility Shift Assay (EMSA)

To assess the potential biological relevance of DNA methylation differences caused by tobacco smoking, methylation-specific DNA-protein binding analysis by electrophoretic mobility shift assay (EMSA) was carried out exemplarily for CpG site cg05575921 (*AHRR*), the most outstanding site with respect to significance and level of detectable changes in DNA methylation associated with tobacco smoking. Here, we detected methylation-specific DNA-protein binding patterns for this site using both Raji (human B-lymphoblastoid cell line, see Figure S4) and THP1 (human monocytic cell line) nuclear extracts in two independent EMSA experiments for each cell line. DNA-protein complex C1 showed higher binding affinity to the methylated site, whereas complexes C2 and C3 preferably bound to the unmethylated state of cg05575921. Binding specificity was validated by using a competitive approach (unmethylated probe competing with methylated and unmethylated probe (lanes 4–7), methylated probe competing with unmethylated and methylated probe (lanes 11–14), and both probes competing with an unrelated SP1-probe (lanes 8/9 and 15/16)).

Corroborating this observation, Monick et al. recently showed that an increase in methylation at cg05575921 was associated with a decrease in lymphoblast *AHRR* gene expression (p<0.03, N = 108) [12]. And, as mentioned earlier, the study by Bosse et al. found AHRR to be the most significant probe set between never and current smokers with a 6.1 fold change [43]. Furthermore, a recent study of Shenker et al. demonstrated AHRR expression to be 5.7 fold increased in human lung samples from current vs. never smokers, which inversely correlated with methylation levels [17]. This underscores our EMSA findings and suggests that this CpG site may have a regulatory role on gene expression, possibly mediated by differential binding of methylation-specific transcription factors, the identification of which may be the subject of future studies.

### Strengths and Limitations

The major strengths of our study are the relatively large sample size of the population-based discovery and the selected replication panel, as well as the information about former smoking. We adjusted for a large number of potential confounders and applied a method of quality assurance (filtering for detection p-value and nearby SNPs) and normalization developed by Touleimat & Tost 2012 [44].

There are also limitations to our study: despite thorough assessment of the smoking status by several questions, we do not have cotinine measurements in the KORA study to directly assess smoking burden. Passive smoking, which might also have an effect on DNA methylation, was not taken into account. The design of the present study is cross-sectional in nature; therefore we can only suggest that quitting tobacco smoking presumably allows reformation of the DNA methylation state of never smokers. Longitudinal studies are needed to confirm these results. Furthermore, the present study explores whole blood, which consists of a complex composition of cells that show individual methylation patterns [45]. However, Shenker et al. analyzed the relationship between different blood cell fractions and whole blood DNA from the same individual by the 450K. These analyses could show no evidence that any of the blood cell types have significantly different methylation levels that would confound an association with smoking. In addition, the methylation levels of sites in the *AHRR* gene between lung tissues and PBMCs were compared and found to be identical [17]. Furthermore, a similar correlation has also been reported in lymphoblasts and pulmonary macrophages by Monick et al. [12]. Additionally, several of the smoking-associated genes we were able to detect (*AHRR*, *GFI1*, *MYO1G*, *CNTNAP2*) were also reported to be differentially methylated in cord blood samples due to maternal smoking. This study by Joubert et al., directly addressed the potential impact of differential cell counts by additionally measuring polymorphonuclear and mononuclear cells with the 450K BeadChip. The differences in methylation by cell type were much smaller than the differences in methylation by smoking observed in whole blood, indicating that their findings are not explained by cell type confounding [16].

These studies show, and strengthen our findings, that even though DNA methylation is tissue specific, and the sensitivity depends on the tissue type, changes in DNA methylation may at least in some cases be reflected in whole blood. This certainly has high clinical relevance, as blood is an easily accessible biomaterial and therefore an attractive tissue for the identification and subsequent use of biomarkers.

### Conclusions

In summary, we observe evidence of significant differences in the degree of site-specific methylation in each of the 22 autosomes as a function of tobacco smoking, identifying 187 differentially-methylated CpG sites by array-based DNA methylation analysis. The corresponding genes play roles mostly in the development and function of the cellular, hematological, immune, cardiovascular, tumorigenic or reproduction system. Depending on cessation time and pack-years, methylation levels in former smokers were found to be close to levels seen in never smokers. Methylation-specific protein binding patterns observed in EMSA experiments suggest a regulatory role of CpG site cg05575921 for gene expression.

The results of our study confirm the broad effect of tobacco smoking on the human organism. Revealing the underlying molecular mechanisms that alter the epigenome due to environmental triggers will be an important aspect of future studies.

## Materials and Methods

### Ethics Statement

The study has been conducted according to the principles expressed in the Declaration of Helsinki. Written informed consent has been given by each participant. The study, including the protocols for subject recruitment and assessment and the informed consent for participants, was reviewed and approved by the local ethical committee (Bayerische Landesärztekammer).

### Study Population

The KORA S4 survey, an independent population-based sample from the general population living in the region of Augsburg, Southern Germany, was conducted in 1999/2001. The standardized examinations applied in the survey (4261 participants) have been described in detail elsewhere [46]–[48]. A total of 3080 subjects participated in a follow-up examination of S4 in 2006–08 (KORA F4), comprising individuals who, at that time, were aged 32–81 years. Methylation data of the discovery panel was analyzed with the 450K BeadChip in a subgroup of 1814 individuals (never, former and current smokers) from the KORA F4 cohort, from whom smoking status was available.

The KORA F3 cohort is a ten years follow-up survey of the KORA S3 survey examined in 1994–1995 as described previously [48], [49]. For the replication panel that was also analyzed with the 450K BeadChip, 479 individuals (never, former and current smokers) from the F3 cohort were selected.

No evidence of population stratification was found in multiple published analyses using the KORA cohort [50]. The KORA F3 and F4 surveys are completely independent with no overlap of individuals.

### Assessment of Smoking Status

The category of current smokers comprised regular smokers (smoking daily) and occasional smokers (not smoking daily). The baseline questionnaire included the smoking status (regular/occasional/former/never smoker), the number of cigarettes smoked daily (for regular smokers only), the largest number of cigarettes ever smoked daily for a whole year (for current and past smokers), and the year of beginning and (in case of past smokers) of stopping smoking. Assuming 20 cigarettes per pack, pack-years were calculated using the formula “(cigarettes per day/20) * number of years smoked”.

### Array-based DNA Methylation Analysis with Infinium Methylation 450K

Genomic DNA (1 µg) from 1814 samples was bisulfite converted using the EZ-96 DNA Methylation Kit (Zymo Research, Orange, CA, USA) according to the manufacturer’s procedure, with the alternative incubation conditions recommended when using the Illumina Infinium Methylation Assay.

Genome-wide DNA methylation was assessed using the Illumina HumanMethylation450 BeadChip, following the Illumina Infinium HD Methylation protocol. This consisted of a whole genome amplification step using 4 µl of each bisulfite converted sample, followed by enzymatic fragmentation and application of the samples to BeadChips (Illumina). The arrays were fluorescently stained and scanned with the Illumina HiScan SQ scanner. The percentage of methylation of a given cytosine is reported as a ß-value, which is a continuous variable between 0 and 1, corresponding to the ratio of the methylated signal over the sum of the methylated and unmethylated signals. The M-value is calculated as the log2 ratio of the intensities of methylated probe vs. unmethylated probe [51].

### Data Pre-processing and Initial Quality Assessment

GenomeStudio (version 2010.3) with methylation module (version 1.8.5) was used to process the raw image data generated by BeadArray Reader. Initial quality assessment of assay performance was conducted using the “Control Dashboard” in the software package and included assessment of DNP and Biotin staining, extension, hybridization, target removal, bisulfite conversion, specificity, negative and non-polymorphic controls.

9 samples of F4 and none of F3 had to be excluded because of deviations from optimal performance that also remained when the complete Illumina Infinium HD Methylation protocol was repeated, suggesting insufficient DNA quality.

For data pre-processing of the Infinium Human Methylation 450K BeadChip we used the pipeline described in Touleimat & Tost 2012 with default parameter settings to avoid bias in the analysis since the assay combines two different chemistries [44]. In brief, prior to normalization three samples with less than 80% high quality probes (detection p-value <0.01) were excluded. CpG sites in close proximity (50bp) to common SNPs were removed. Color bias adjustment based on a smooth quantile normalization method as well as background level correction based on negative-control probes was performed for each chip using the R lumi package [52]. Finally, the pipeline performs a subset quantile normalization in order to correct for the InfI/InfII shift and normalizes between samples. Therefore CpG-categories were built using the ‘relation to CpG-island’ information (South shore, South shelf, North shore, North shelf and distant) from the Illumina file. Please see Table S8 for further information on the number of samples and probes removed prior to data analysis.

### Data Analysis

9 of the 1802 F4 individuals and none of the 479 F3 individuals had to be excluded due to missing information in one or two of the covariates, resulting in a final sample size of 1793 F4 individuals for the Discovery Round and 479 F3 individuals for the Replication Round (including 11 former smokers that were not used for a separate analysis due to small sample size) (Table S8).

Associations between smoking and methylation M-values were analyzed using multivariable linear regression. A particular methylation M-value was the response variable, with smoking status being the explanatory variable and sex, age, BMI, alcohol consumption as well as white blood cell count as covariates. Analyses of current vs. never smokers as well as of former vs. never smokers were performed by means of smoking status coded as a factor variable with three levels. Also an interaction model with sex was calculated, where the interaction of the smoking factor variable with sex was included in the latter model. Besides this, the stratified analyses were calculated for males and females separately. In addition to the earlier described covariates, this model was also adjusted for pack-years, due to the significant difference of this variable in males and females.

In addition, linear models that included former smokers only were calculated with the metric explanatory variables pack-years and/or time since quitting instead of smoking status. As we experienced in a loess curve, the methylation level in former smokers at the majority of CpG sites approached the corresponding level of never smokers within increasing time since quitting, starting approximately from the level of current smokers for those who only recently quit smoking. Therefore, we plotted a smooth loess curve (smooth factor = 0.5) in a scatterplot of methylation (beta-value, only former smokers) and time since quitting, in order to visualize which impact years or decades of cessation might have on DNA methylation. The descriptive median methylation ß-values of current and never smokers are also displayed as a brown respective green line. These plots were used to get an idea of the time since quitting at which the methylation state of former smokers is closer to or equals the one of the original median difference between current smokers and never smokers. The same procedure was carried out with pack-years, to get an idea of the influence of cumulative smoke exposure on the methylation state of former smokers.

The explained variance was calculated in the linear model from the ANOVA table, taking the deviance of the variable (e.g. smoking, pack-years) divided by the null-deviance (i.e. residual deviance in the model without covariates). In calculating the explained variance of smoking, we used a two-stage-variable (never and current respective never and former).

We relied on methylation β-values for the presentation of the scatterplots, since they allow for a straightforward interpretation of the results. In the linear models with covariates we used the M-value, since it shows better statistical ability. The assumption of a normal distribution was verified for all CpG sites that showed a significant result using density plots of the residuals obtained from the multivariable linear regression as well as corresponding QQ plots. All significantly associated sites showed approximately normal distributed residuals except for cg23576855 which was therefore excluded from further analysis.

Regarding the discovery sample (F4), the global significance level of 5% was corrected for multiple comparisons of CpG sites with smoking status, following the Bonferroni procedure (0.05/468316 = approx. p = 1E-07). In the replication sample (F3) the correction was made for the number of significant CpG sites in the discovery sample (0.05/972 = approx. p = 5E-05).

All analyses were performed using the statistical package R Version 2.14 (http://www.r-project.org/), including the packages: base, datasets, graphics, grDevices, methods, stats and utils. The meta-analysis for F4 and F3 was performed with the software METAL (http://www.sph.umich.edu/csg/abecasis/Metal/; release 2011-03-25) with cohorts weighted by their sample size.

### Quantitative DNA Methylation Analysis by MassARRAY EpiTYPER

Validation of the three most significant loci (*AHRR*- cg05575921, *ALPP/ALPPL2*- cg21566642 and *F2RL3*- cg03636183) was carried out by MALDI-TOF mass spectrometry using EpiTYPER by MassARRAY (Sequenom, San Diego, CA) as previously described [53]. The target regions were amplified using the primer pairs and annealing temperatures (T_a_) described in Table S9. The chip was read by the Sequenom MALDI-TOF MS Compact Unit and visualized with the use of MassARRAY EpiTyper v1.2 software (Sequenom).

DNA methylation values were generated as ß-values, determined by comparing the signal intensities between the mass signals of methylated and non-methylated templates, which we transformed into M-values for statistical analysis. Association with smoking status was assessed by linear regression using M-values as the response variable, smoking status as the explanatory variable and sex, age, BMI, alcohol consumption as well as white blood cell count as covariates. Statistical analysis was carried out by R 2.14 (http://www.r-project.org/).

### Electrophoretic Mobility Shift Assays (EMSA)

THP1 and Raji nuclear extracts were purchased from Active Motif (THP1 # 36076, Raji # 36023). Cy5-labelled and unlabelled oligonucleotides containing the methylated or unmethylated CpG site cg05575921 were annealed and purified in a 12% polyacrylamide gel. The binding reaction was carried out with or without different concentrations of unlabeled competitor oligonucleotides using 5 µg of nuclear extract in 1x binding buffer (4% v/v Glycerol, 1 mM MgCl2, 0.5 mM EDTA, 0.5 mM DTT, 50 mM NaCl, 10 mM TrisHCl pH7.5) with 0.5 µg poly dI-dC (Roche Diagnostics) and 1 ng of labeled probe in a total volume of 10 µl for 20 min at 4°C. Protein-DNA complexes were separated on a 5.3% polyacrylamide gel by electrophoresis in 0.5×tris-borate-EDTA (TBE) buffer. The gels were visualized by scanning with the Thyphoon Trio+(GE Healthcare).

## Supporting Information

Figure S1 **Overview of the results for** ***AHRR*** **and** ***ALPP/ALPPL2*** **.** The gene structures and the significant differentially-methylated CpG sites of a) *AHRR* (aryl hydrocarbon receptor (*AHR*) repressor) and b) *ALPP/ALPPL2* (alkaline phosphatase, placental/placental-like) are displayed in current compared to never smokers of the F4 discovery panel. CpG sites which remain significant in the replication panel F3 are framed; CpG sites that were found to still be significant in former smokers are underlined.
(TIF)

Figure S2 **Influence of time since quitting on the DNA methylation state in former smokers.** Illustrated by a loess curve in the scatterplots are the years needed for former smokers to acquire a median ß-value methylation state at single CpG sites that is closer to or equals the one of never smokers; the x-axis displays the cessation time in years, the y-axis displays the methylation level with the use of numbers between 0 (for 0% methylation) and 1 (for 100% methylation); horizontal brown line: median methylation level of current smokers; horizontal green line: median methylation level of never smokers; horizontal grey line: center line of current and never smokers median ß-value methylation; please see Table 4 for detailed data.
(PDF)

Figure S3 **Influence of cumulative smoking exposure (pack-years) on the DNA methylation state in former smokers.** The pack-years needed for former smokers to achieve a median ß-value methylation state at single CpG sites that is closer to or equals the one of current smokers is displayed by a loess curve in the scatterplots; the x-axis displays the number of pack-years, the y-axis displays the methylation level with the use of numbers between 0 (for 0% methylation) and 1 (for 100% methylation); horizontal brown line: median methylation of current smokers; horizontal green line: median methylation of never smokers; horizontal grey line: center line of current and never smokers median ß-value methylation; please see Table S6 for detailed data.
(PDF)

Figure S4 **Methylation specific protein binding patterns of the CpG site cg05575921 in the** ***AHRR*** **gene.** Methylated and unmethylated Cy5-labelled probes carrying the cg05575921 site were used in competition EMSAs using Raji and THP1 nuclear extracts. This figure shows one representative experiment of an EMSA using Raji nuclear extracts. Arrows indicate shifted protein-DNA complexes showing methylation specific binding patterns (C1–C3). In lane 1+2, free oligonucleotides without incubation with nuclear extracts are shown. Lane 3+10 show the results for EMSAs for the unmethylated and methylated variant without competition. In lane 4, 5, 11, 12 competitions with the unlabeled adverse oligonucleotides were performed, whereas competitions with the same unlabeled oligonucleotides were performed in lane 6, 7, 13, 14. To ensure specificity, competitions with unlabeled SP1-consensus oligonucleotides were performed in lane 8, 9, 15, 16. (me)cg: methylated c05575921, SP1 = Specificity protein 1. The experiment using THP1 nuclear extracts resulted in comparable methylation specific band patterns (data not shown).
(TIF)

Table S1 **Significant differentially-methylated CpG sites of current compared to never smokers discovered in F4 and corresponding results of former smokers.** Displayed are a) the results of the linear model calculated with M-value adjusted for age, sex, BMI, alcohol and white blood cell count (p-value), as well as the median ß-value methylation difference between current and never smokers for the discovery panel (F4) with genome-wide significance (p≤1E-07) and b) the corresponding results of the same CpG sites for former smokers; sorted by chromosome and mapinfo (Genome build 37).
(XLS)

Table S2 **Significant differentially-methylated CpG sites of current compared to never smokers discovered in F4 and replicated in F3.** Displayed are a) the results of the linear model calculated with M-value adjusted for age, sex, BMI, alcohol and white blood cell count (p-value and explained variance), as well as the median ß-value methylation difference between current and never smokers for the discovery panel (F4) with genome-wide significance (p≤1E-07) and b) the corresponding results of the same CpG sites for the replication panel F3 for comparison (p≤5E-05); (c) the corresponding p-value gained by meta-analysis of F4 and F3; sorted by chromosome and mapinfo (Genome build 37).
(XLS)

Table S3 **Significant differentially-methylated CpG sites of current compared to never smokers in males and females.** Displayed are the results of the linear model calculated with M-value adjusted for age, BMI, alcohol, white blood cell count and pack-years (p-value and explained variance), as well as the median ß-value methylation difference between current and never smokers for the a) male and b) female subpopulation of F4 with genome-wide significance (p≤1E-07); sorted by chromosome and mapinfo (Genome build 37).
(XLS)

Table S4 **Characteristics of the study populations for EpiTYPER methylation analysis.**
(XLS)

Table S5 **Validation by EpiTYPER MassARRAY.** Displayed are the results of current vs. never smokers of the linear model adjusted for age, sex, BMI, alcohol and white blood cell count for the three most significant loci (*AHRR*- cg05575921, *ALPP/ALPPL2*- cg21566642 and *F2RL3*- cg03636183).
(XLS)

Table S6 **The effect of cumulative smoke exposure (pack-years) on DNA methylation.** The results of the linear model for pack-years are displayed, with genome-wide significance level p≤1E-07, calculated with M-value and adjusted for age, sex, BMI, alcohol and white blood cell count (p-value and explained variance), including former smokers of F4 only, as well as the median ß-value methylation levels for current, never and former smokers; sorted by chromosome and mapinfo (Genome build 37).
(XLS)

Table S7 **The combined effect of cessation time and cumulative smoke exposure on DNA methylation.** The results of the linear model are displayed, calculated with M-value adjusted for age, sex, BMI, alcohol and white blood cell count for former smokers of F4 for a) time since quit, b) pack-years, c) time since quit after adjustment for pack-years, d) pack-years after adjustment for time since quit with genome-wide significance level p≤1E-07; sorted by chromosome and mapinfo (Genome build 37).
(XLS)

Table S8 **Number of samples and probes removed prior to 450K data analysis.**
(XLS)

Table S9 **Sequences of PCR tagged primers used for EpiTYPER methylation analysis, product size of each amplicon and informative CpG sites per amplicon.**
(XLS)

Box S1 **Description of genes that correspond to CpG sites with a methylation difference of more than 5% in current vs. never smokers (in addition to** ***AHRR*** **and** ***ALPP/ALPPL2*** **).**
(PDF)
