# Supplementary figures and images for: Tobacco Smoking Leads to Extensive Genome-Wide Changes in DNA Methylation
Source: PLoS One. 2013 May 17;8(5):e63812. doi: 10.1371/journal.pone.0063812 (PMC3656907; doi:10.1371/journal.pone.0063812)

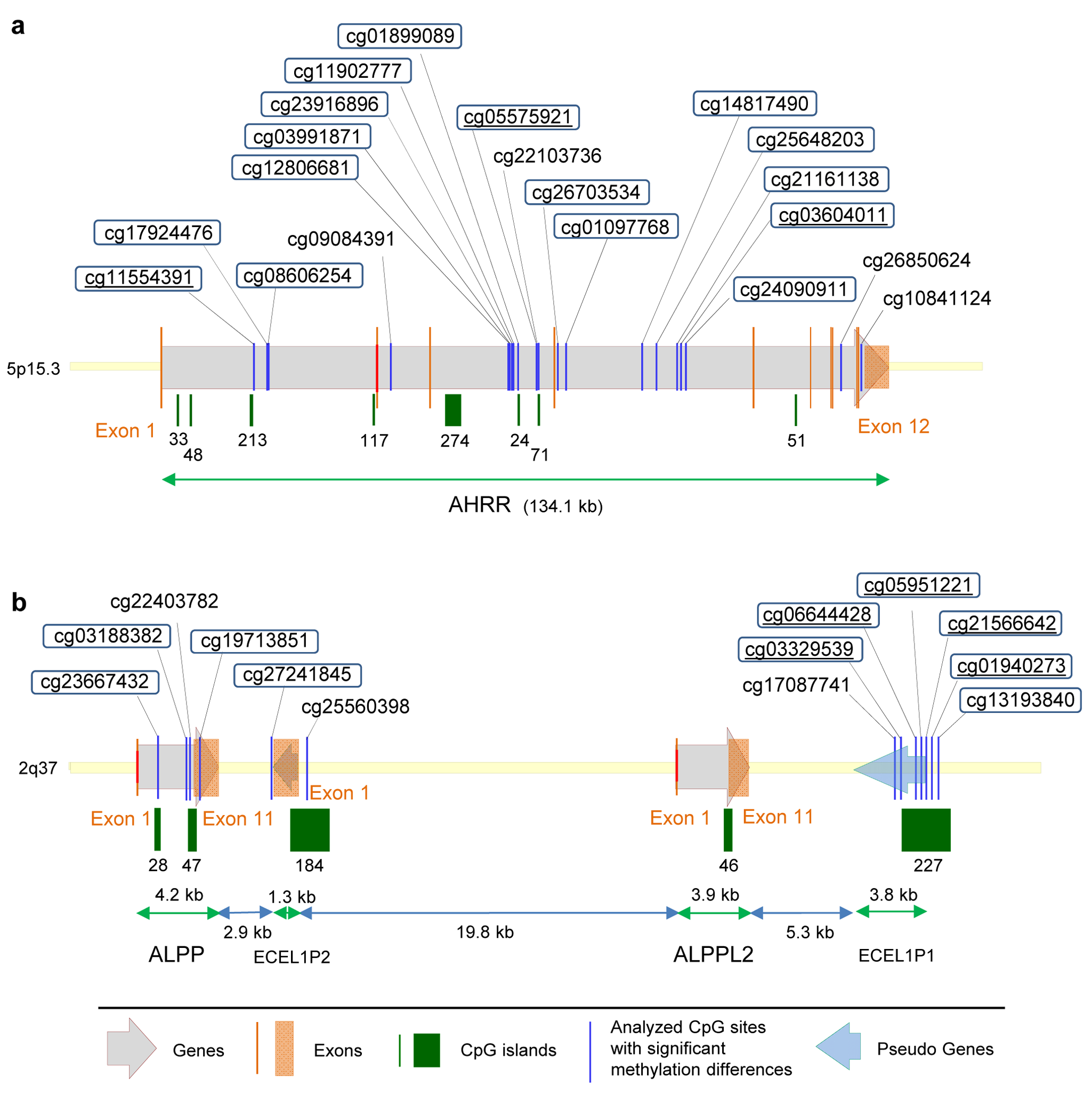

Supplement: Figure S1 — Overview of the results for AHRR and ALPP/ALPPL2 . The gene structures and the significant differentially-methylated CpG sites of a) AHRR (aryl hydrocarbon receptor (AHR) repressor) and b) ALPP/ALPPL2 (alkaline phosphatase, placental/placental-like) are displayed in current compared to never smokers of the F4 discovery panel. CpG sites which remain significant in the replication panel F3 are framed; CpG sites that were found to still be significant in former smokers are underlined. (TIF) [file pone.0063812.s001.tif]

**cg00871610**

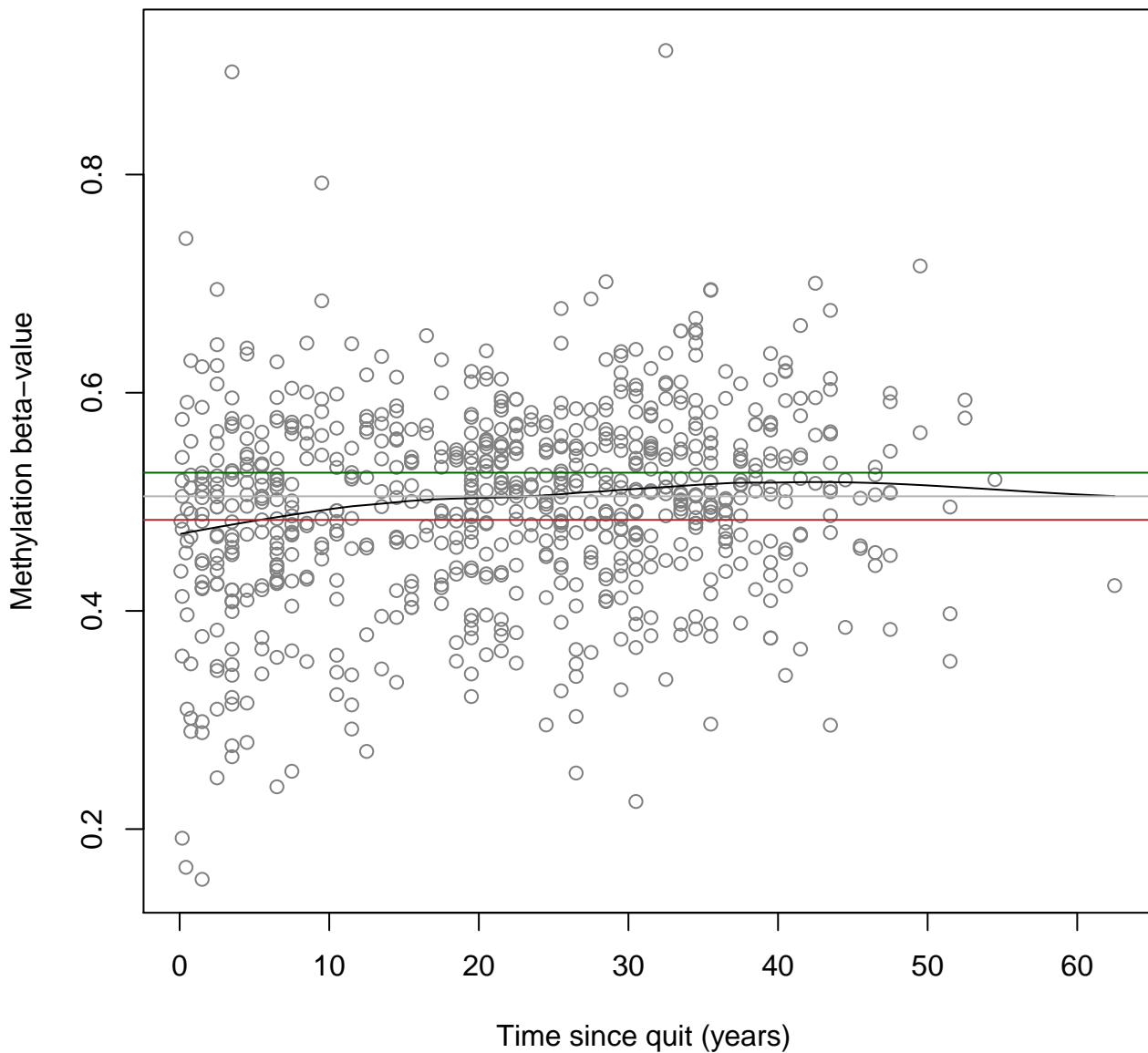

**cg01127300**

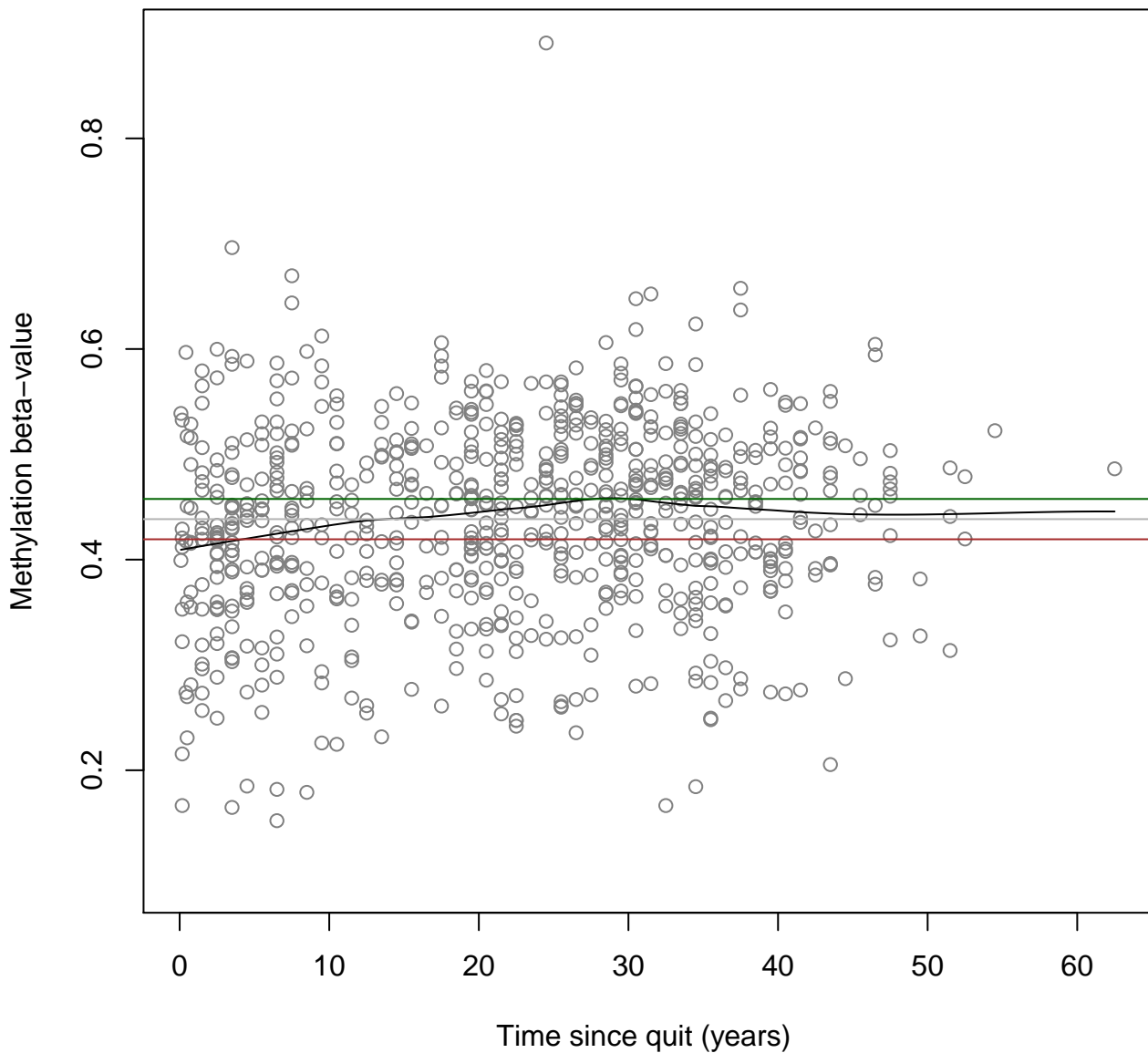

**cg01940273**

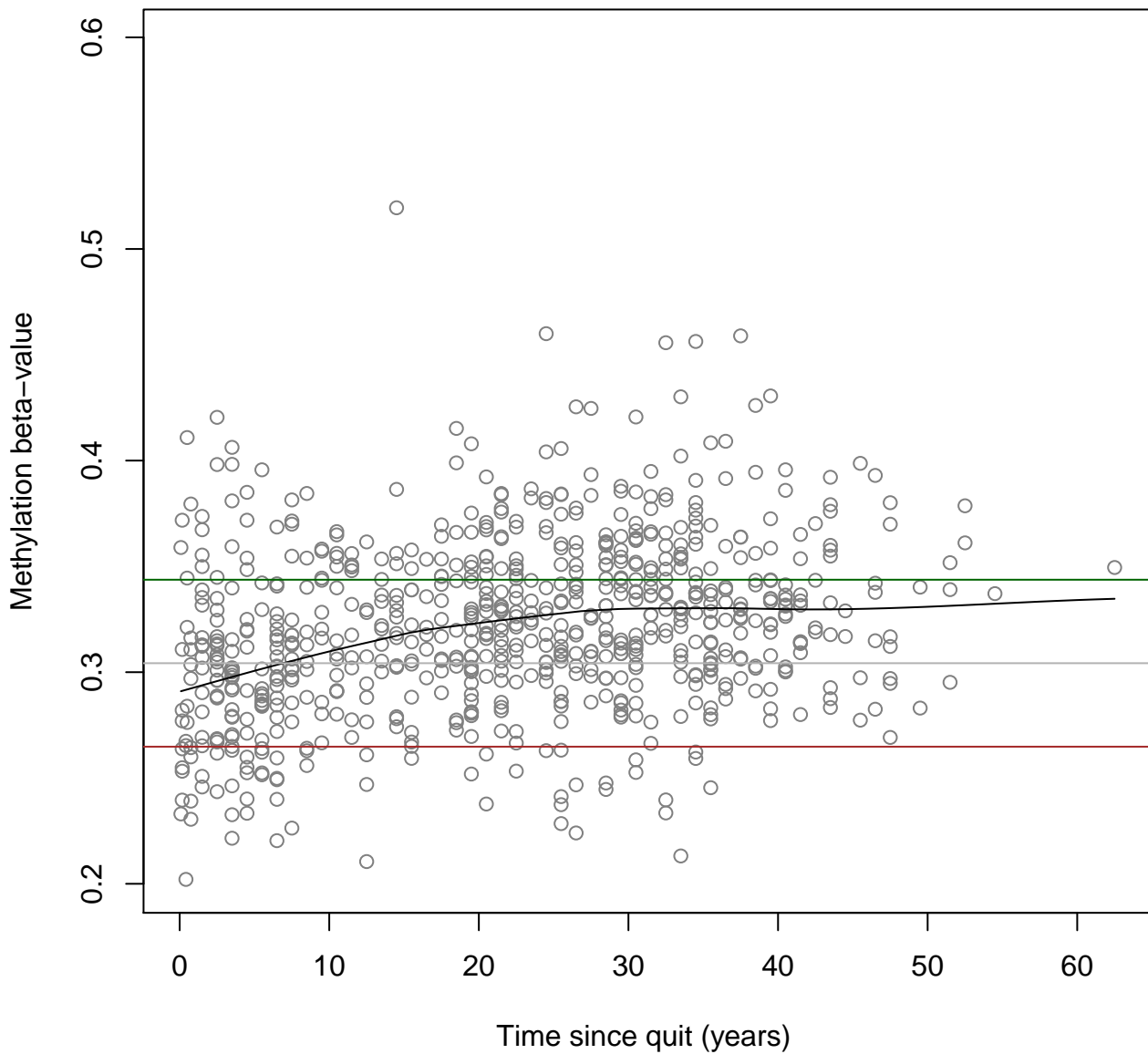

**cg02583484**

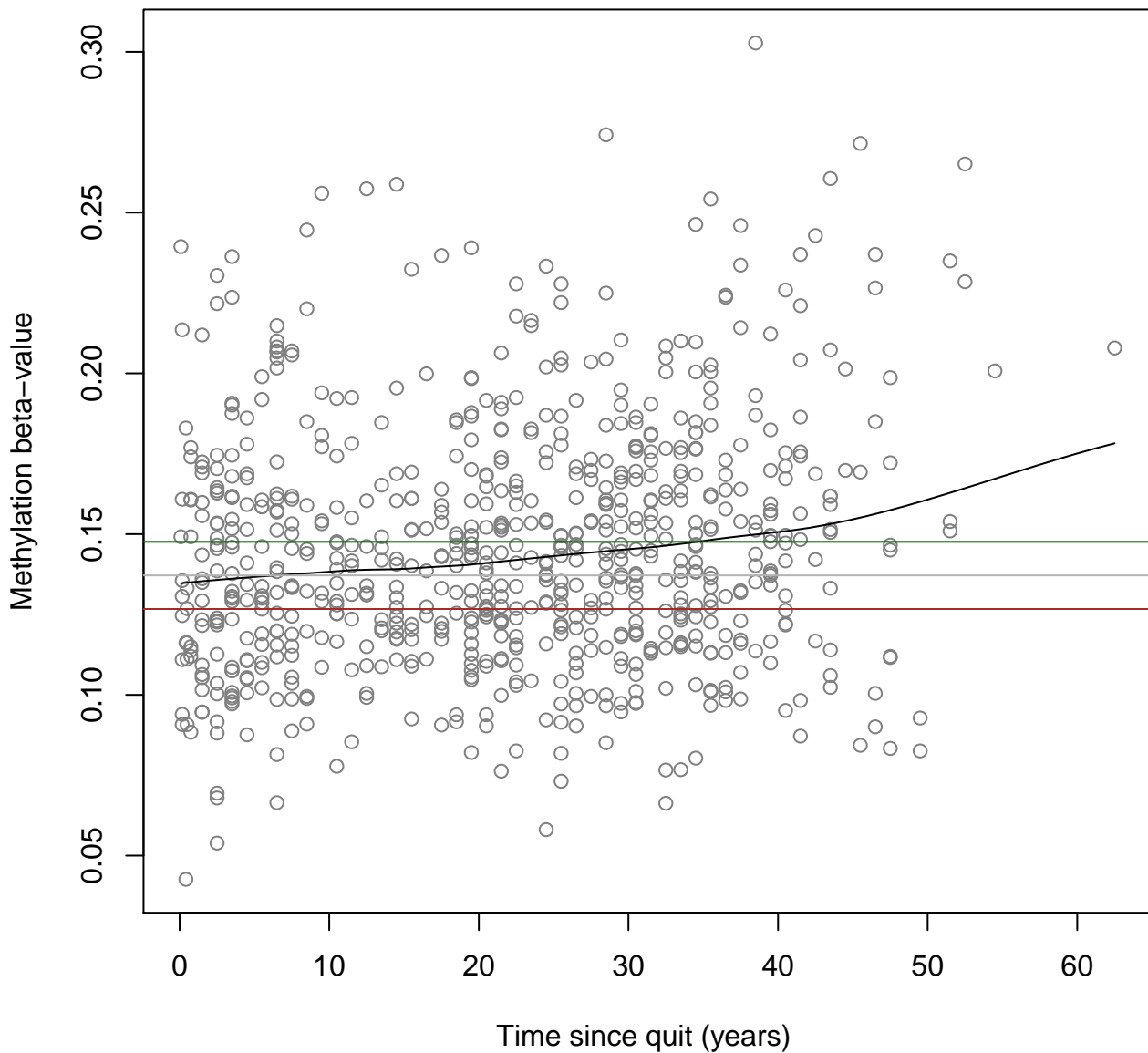

**cg03274391**

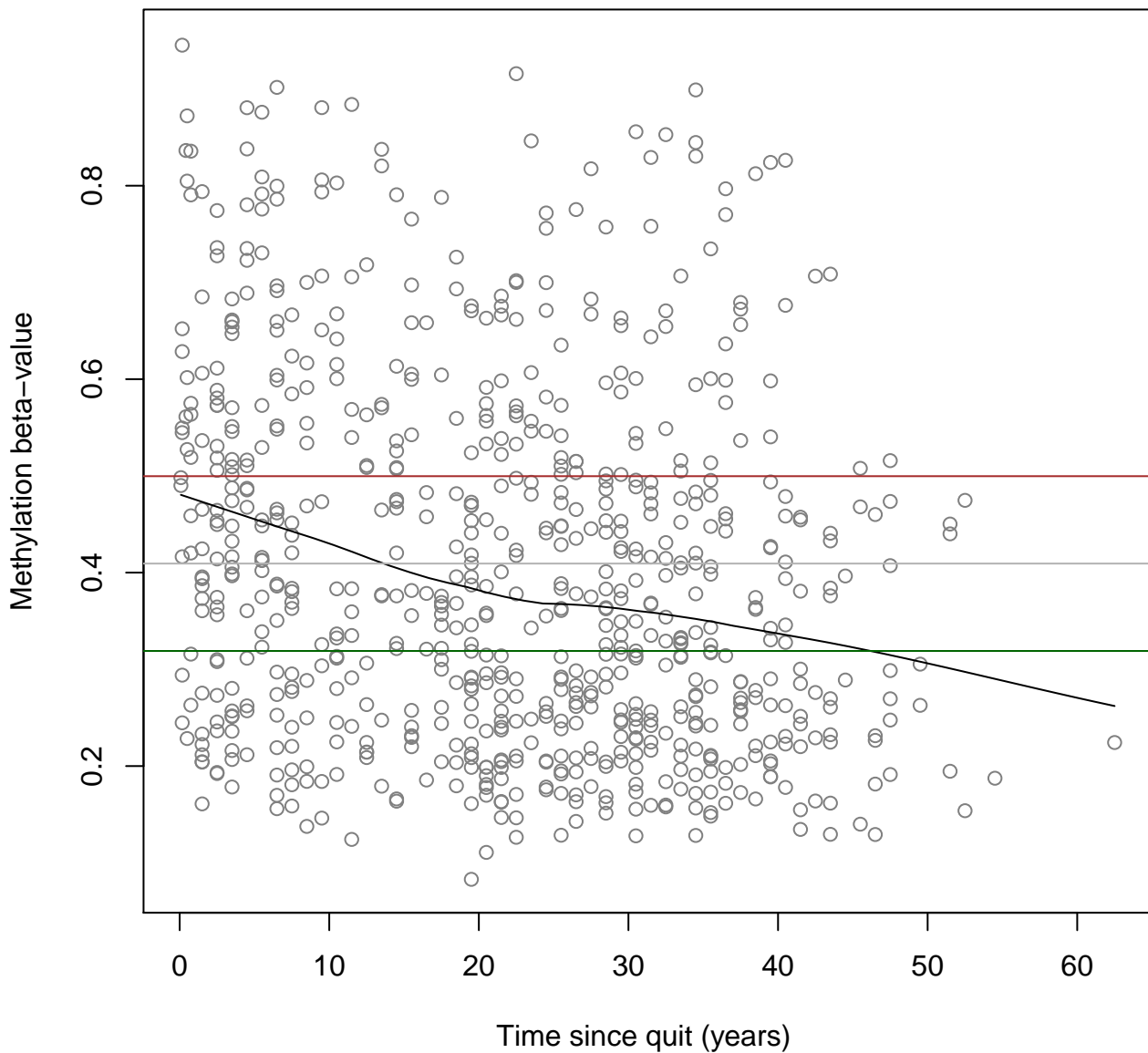

**cg03329539**

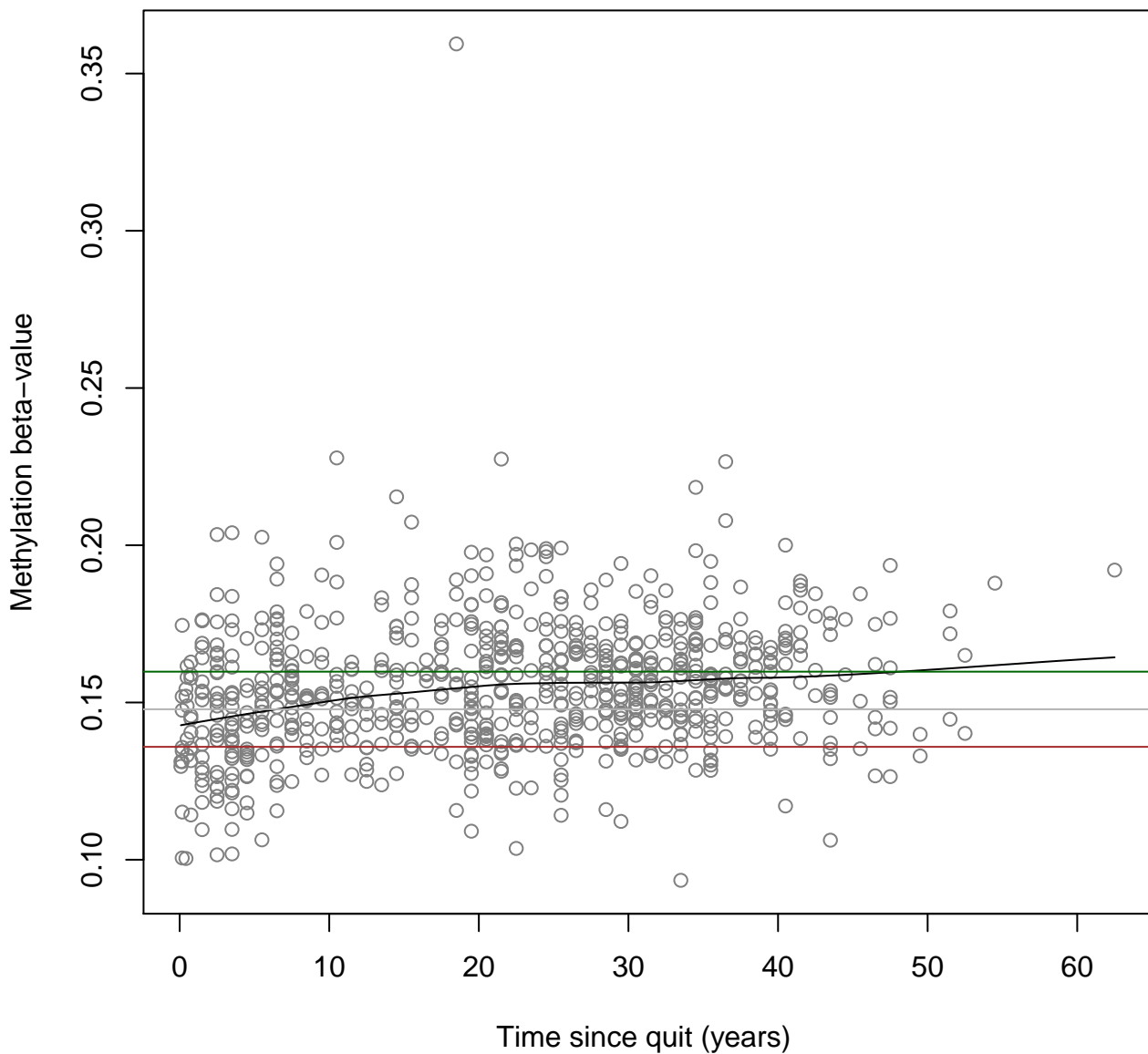

**cg03604011**

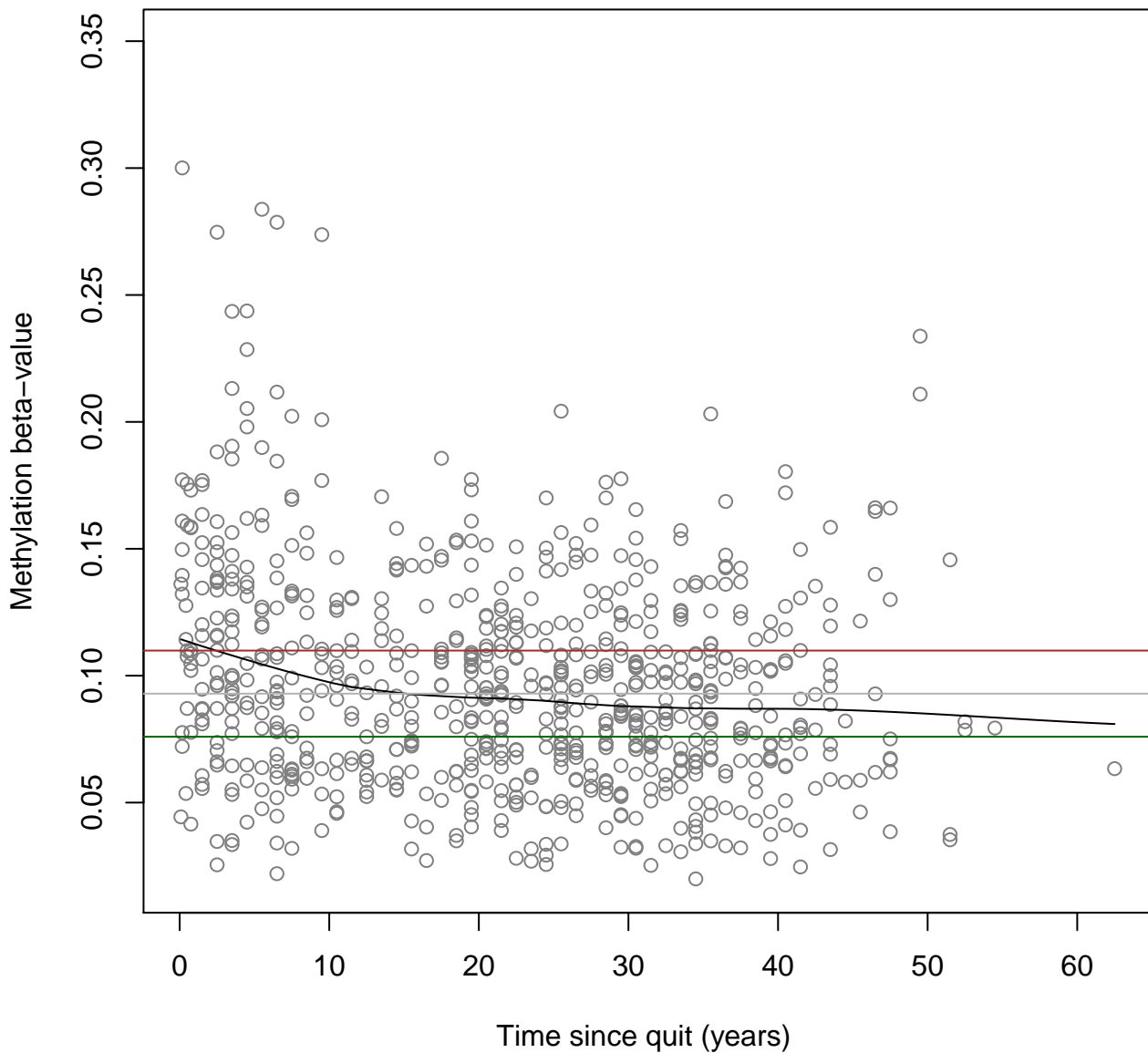

**cg03636183**

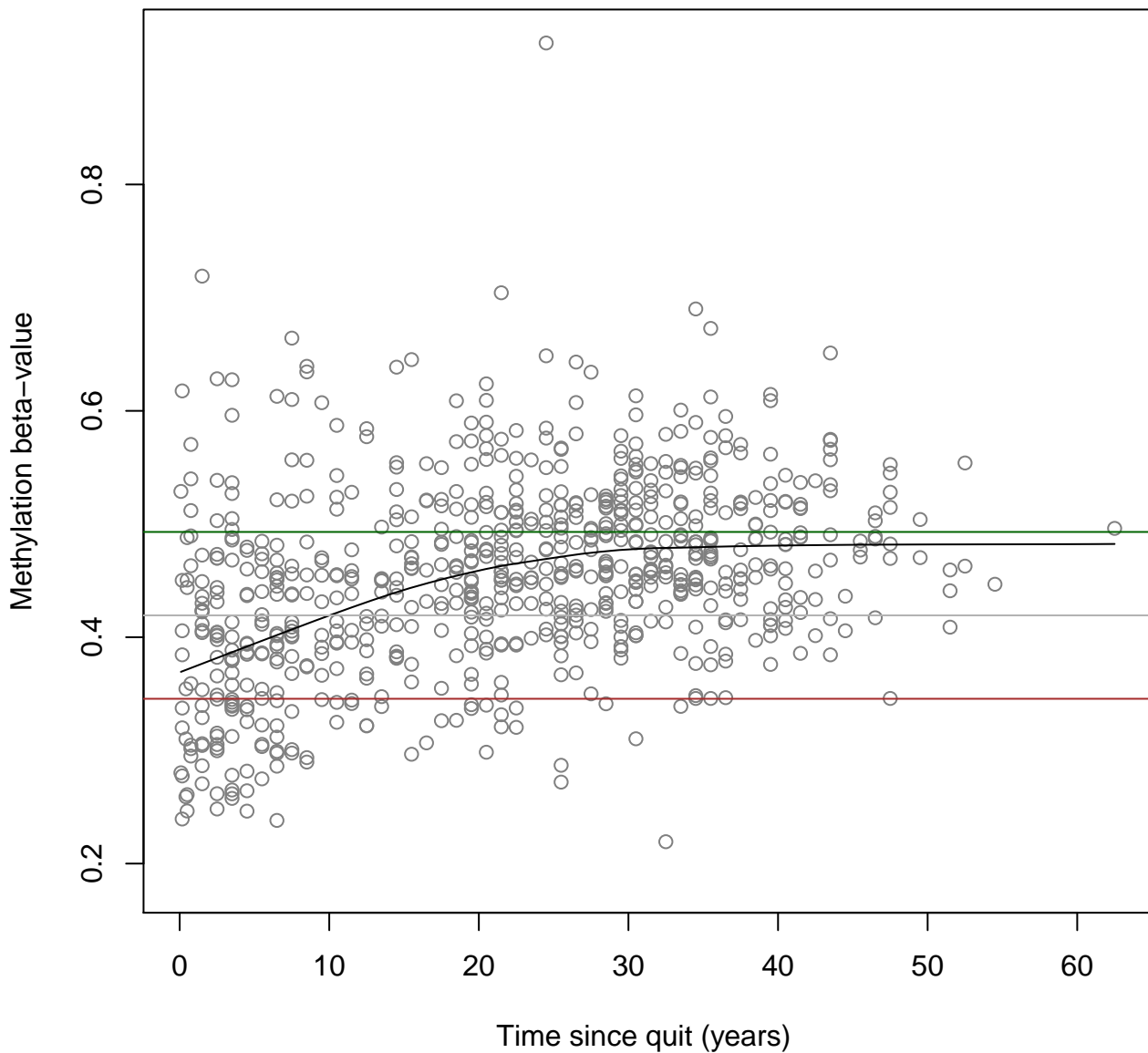

**cg03991871**

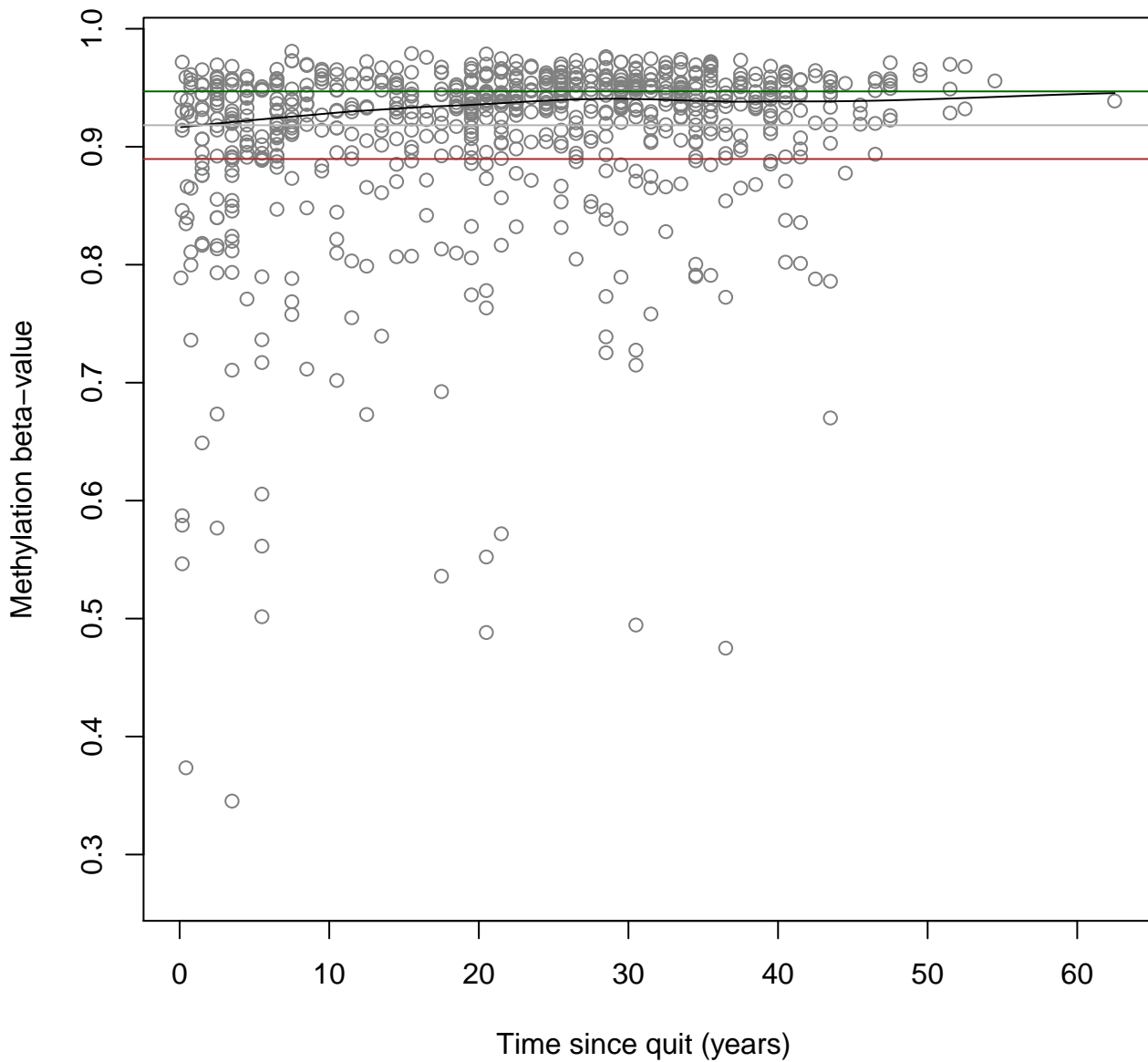

**cg05575921**

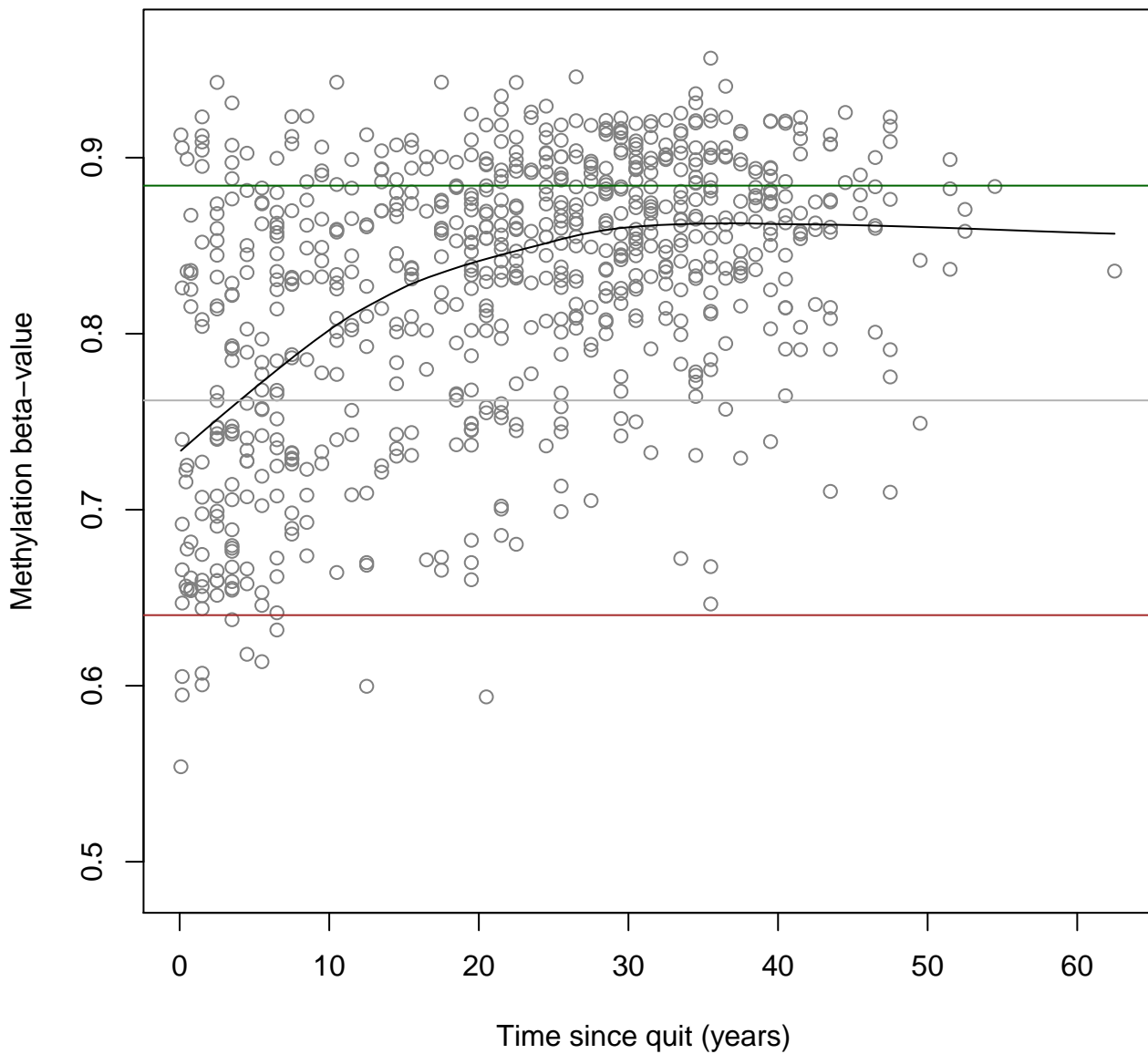

**cg05951221**

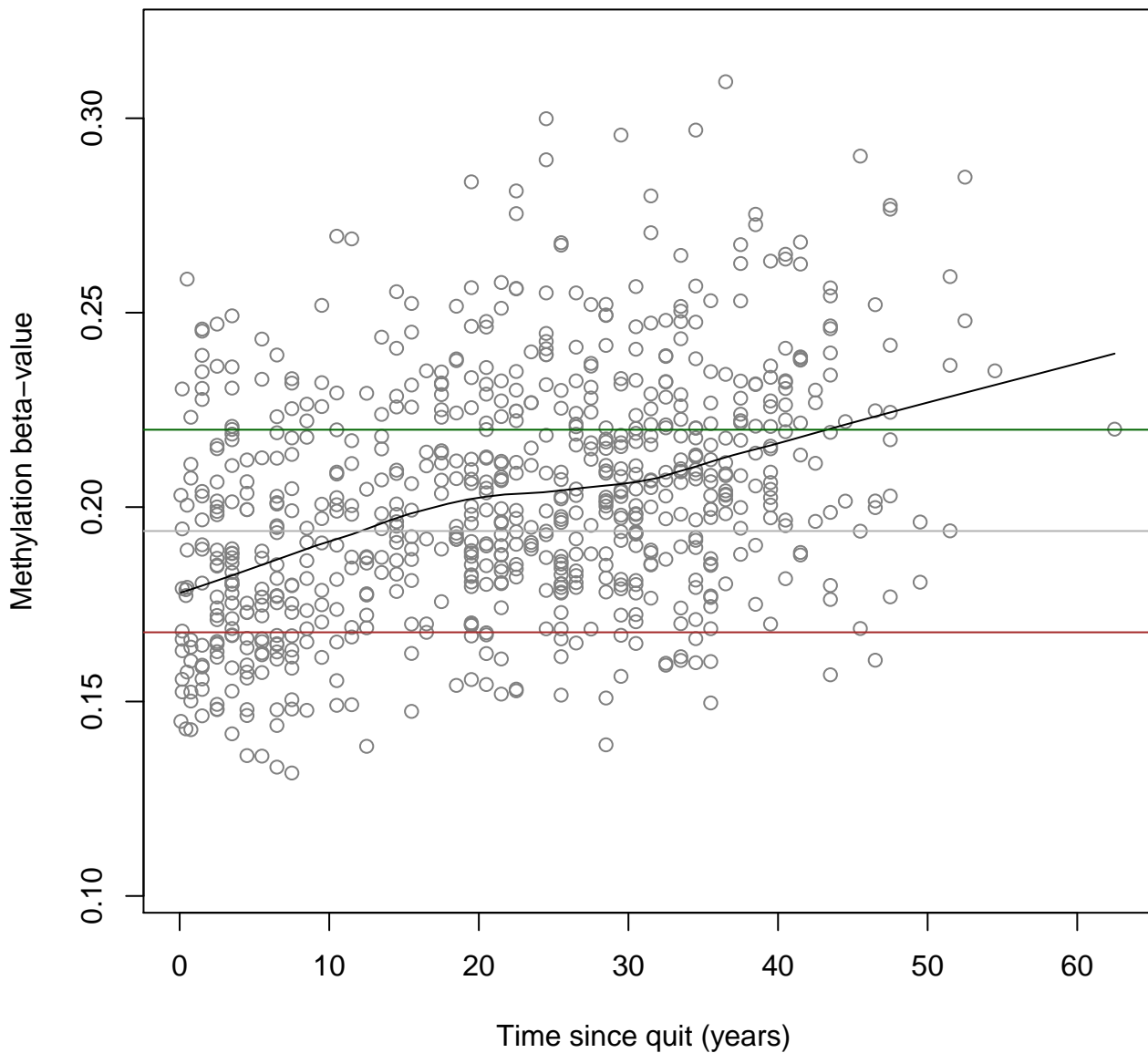

**cg06126421**

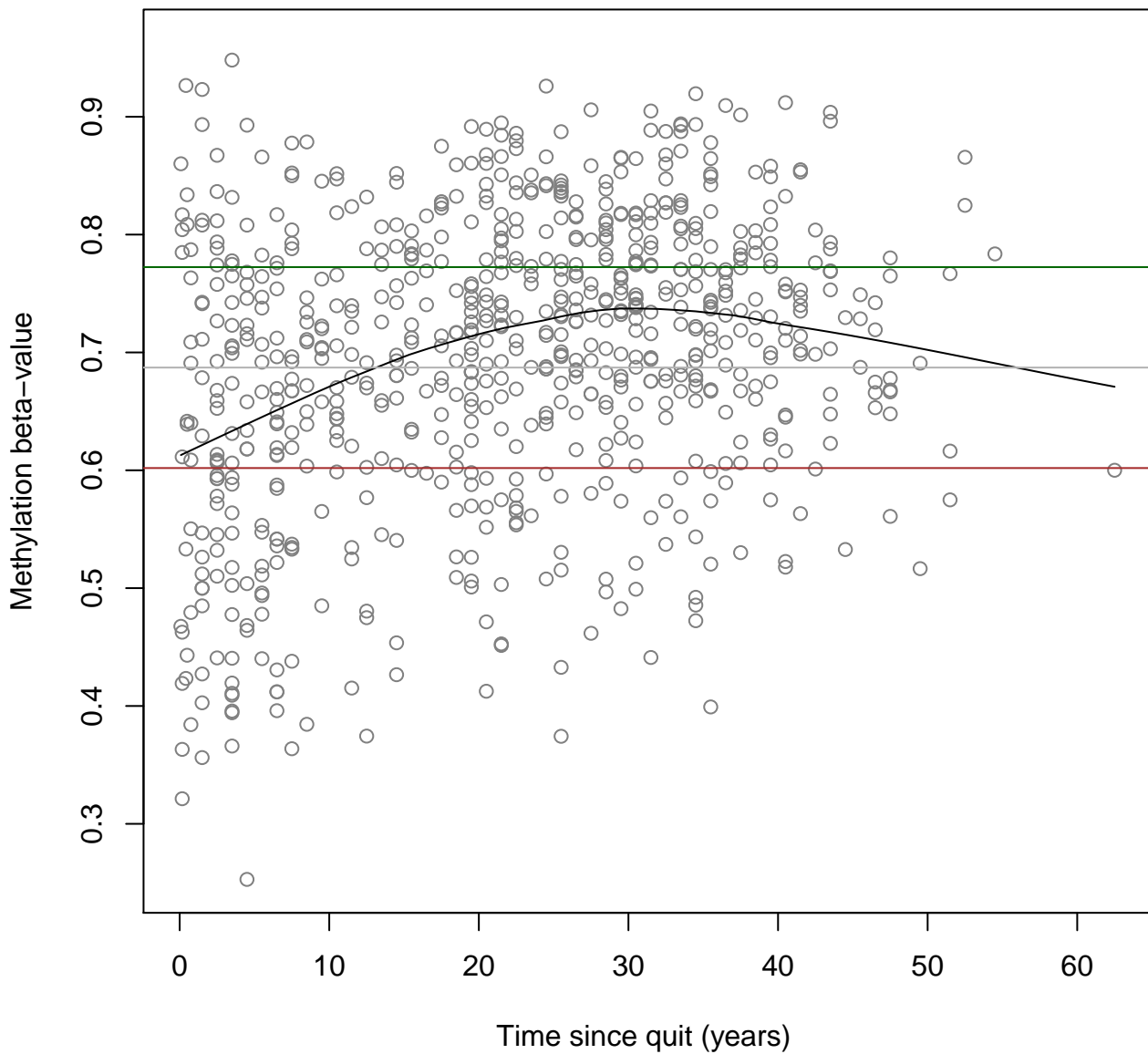

**cg06644428**

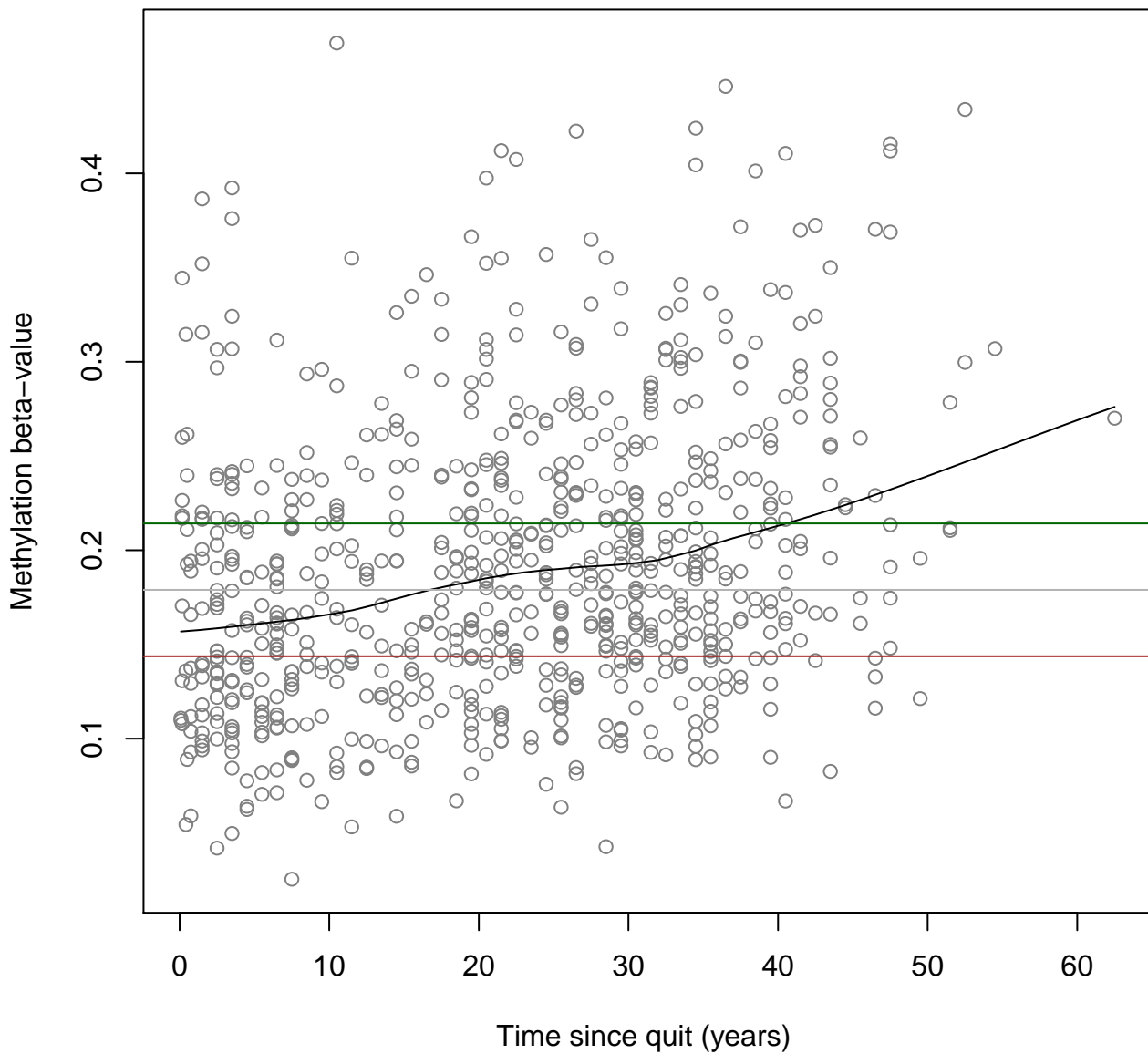

**cg09084200**

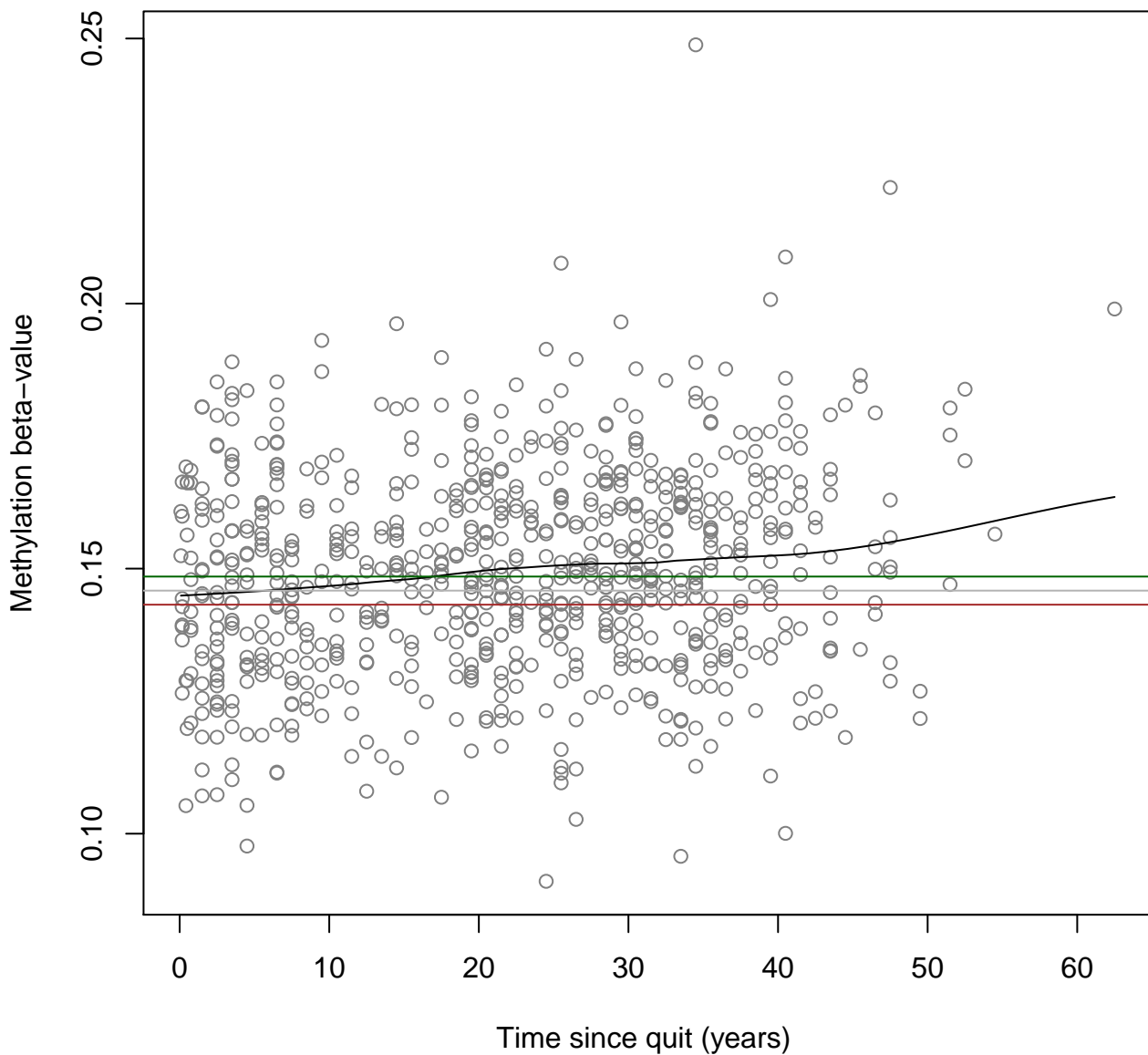

# cg11554391

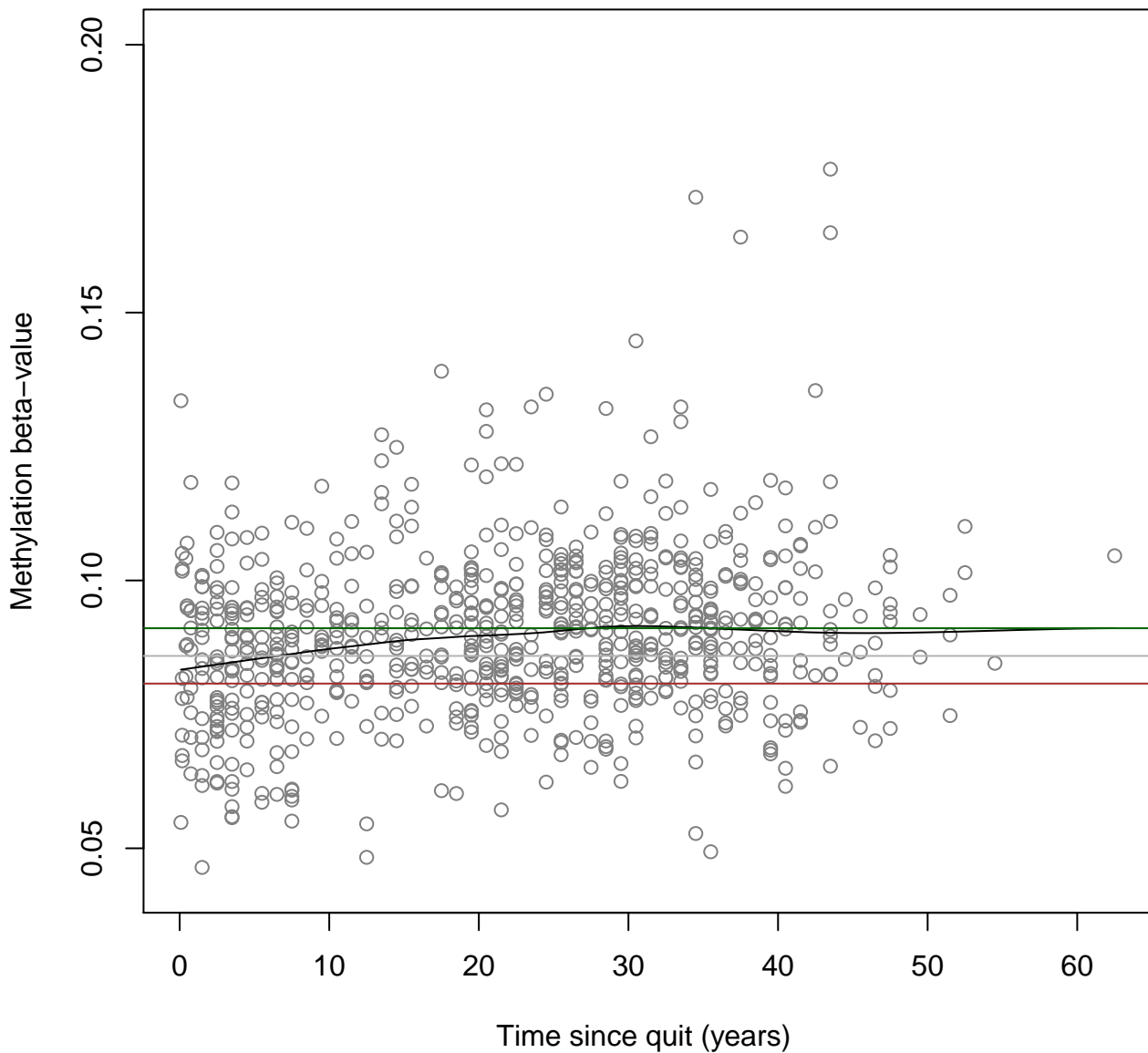

# cg11660018

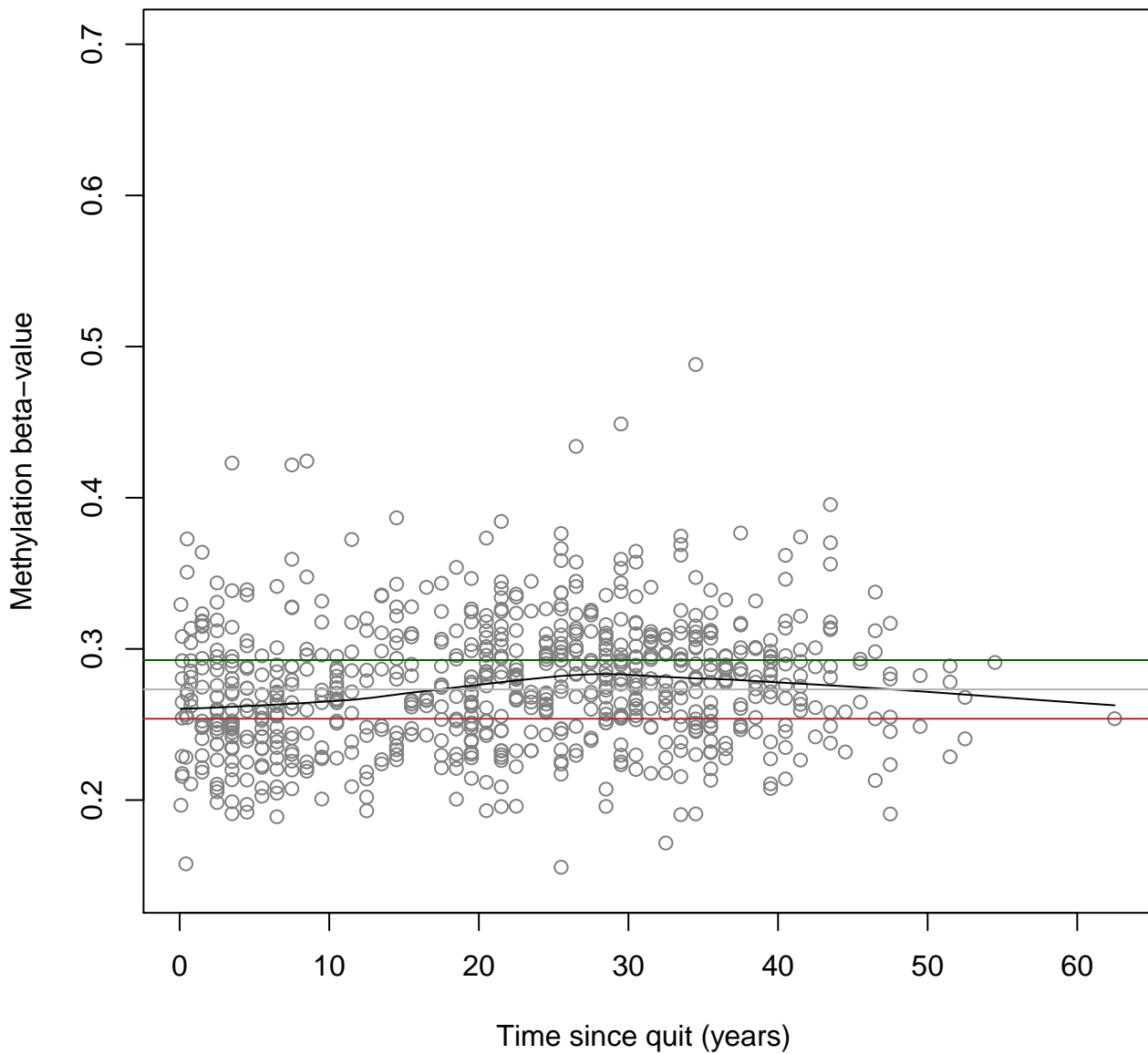

# cg11902777

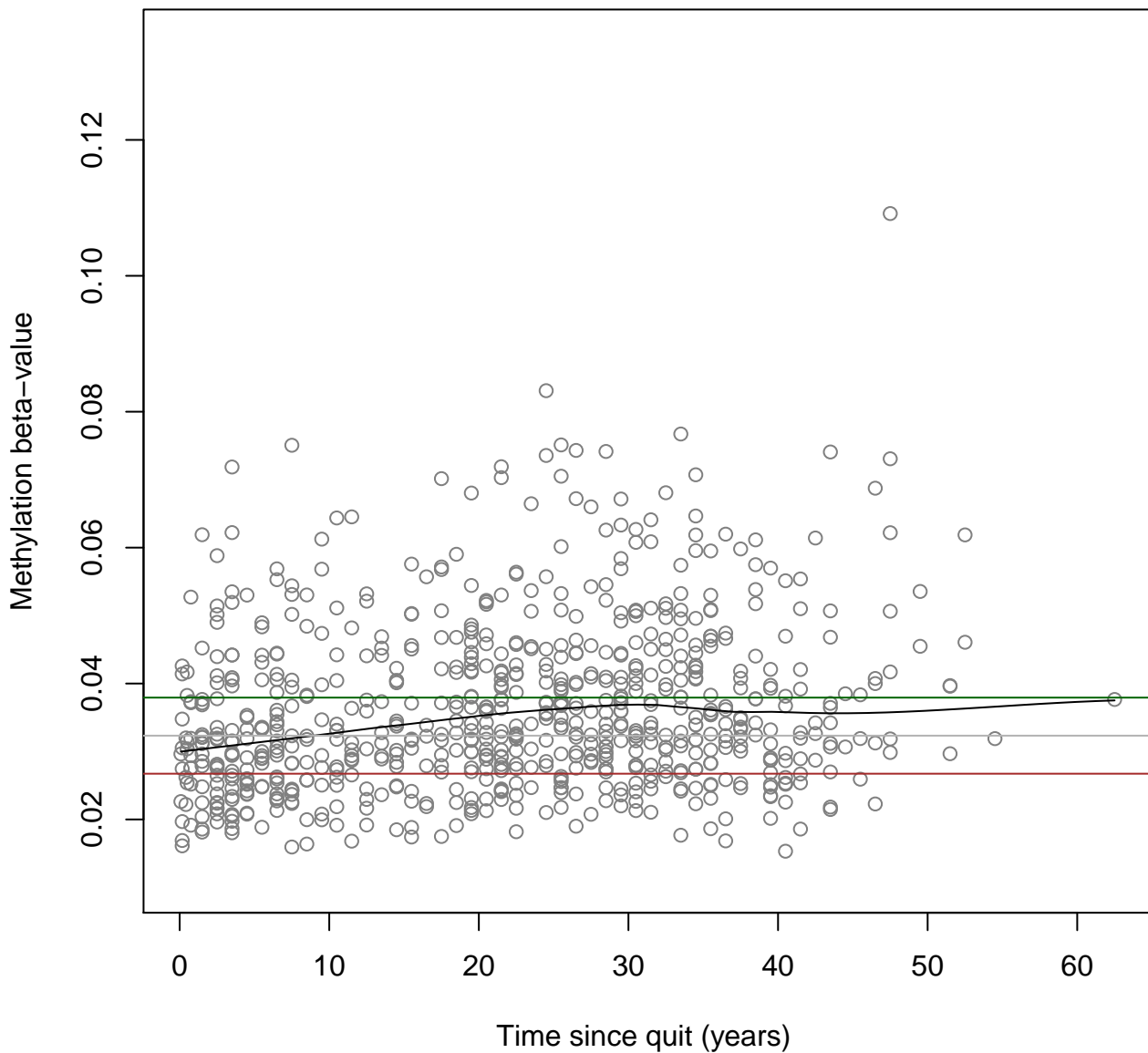

# cg12303084

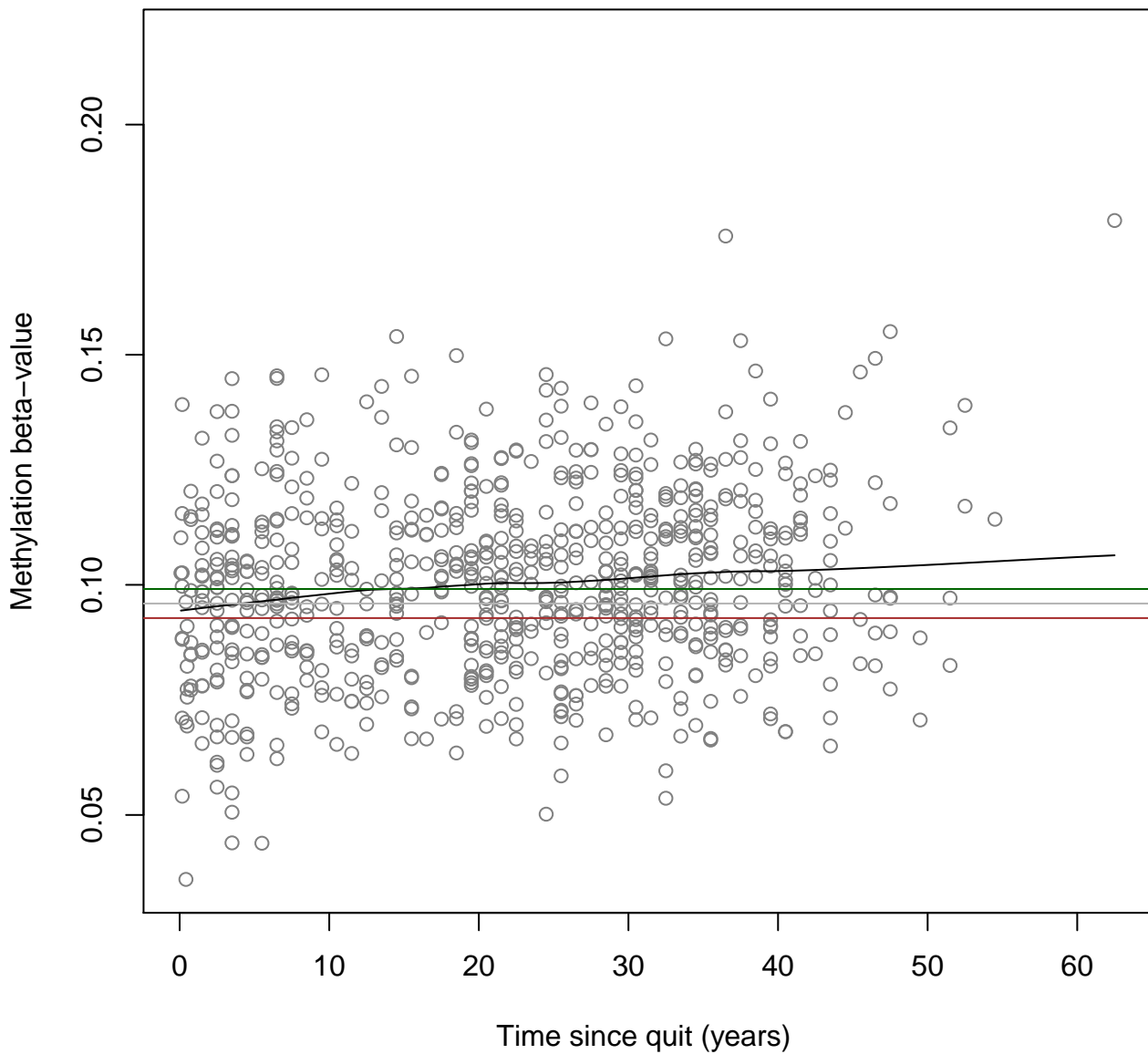

# cg12803068

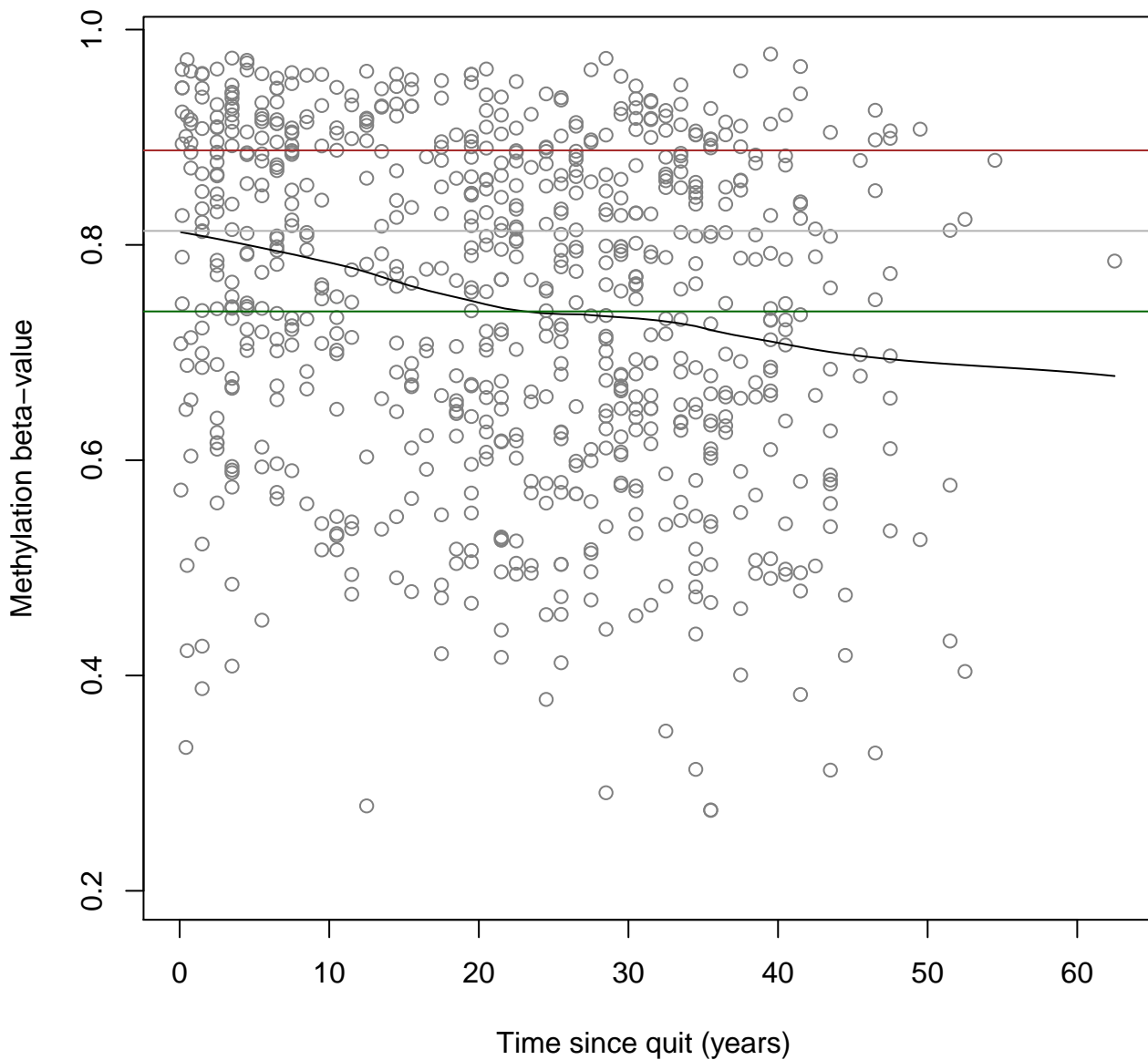

# cg14753356

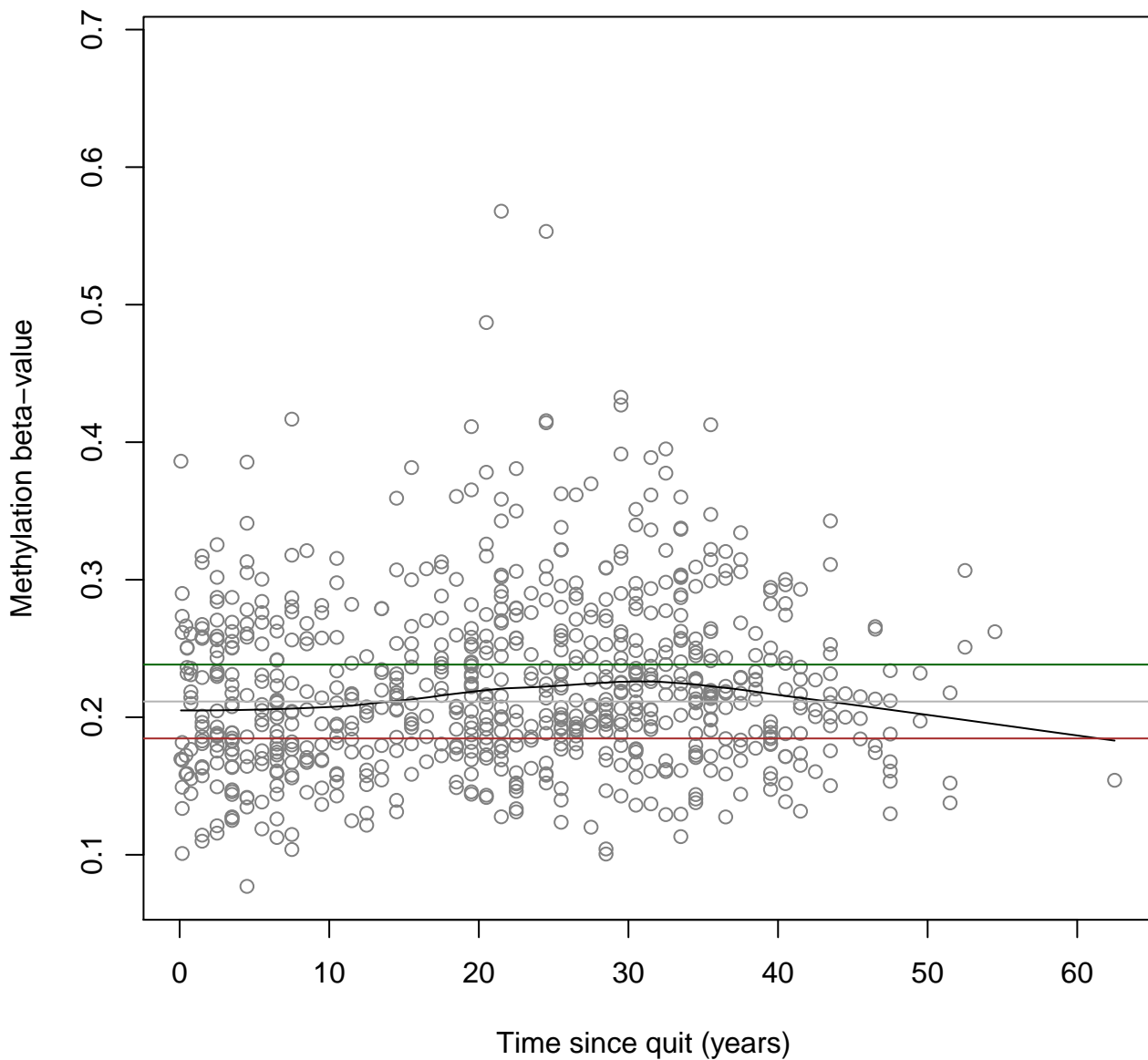

# cg14817490

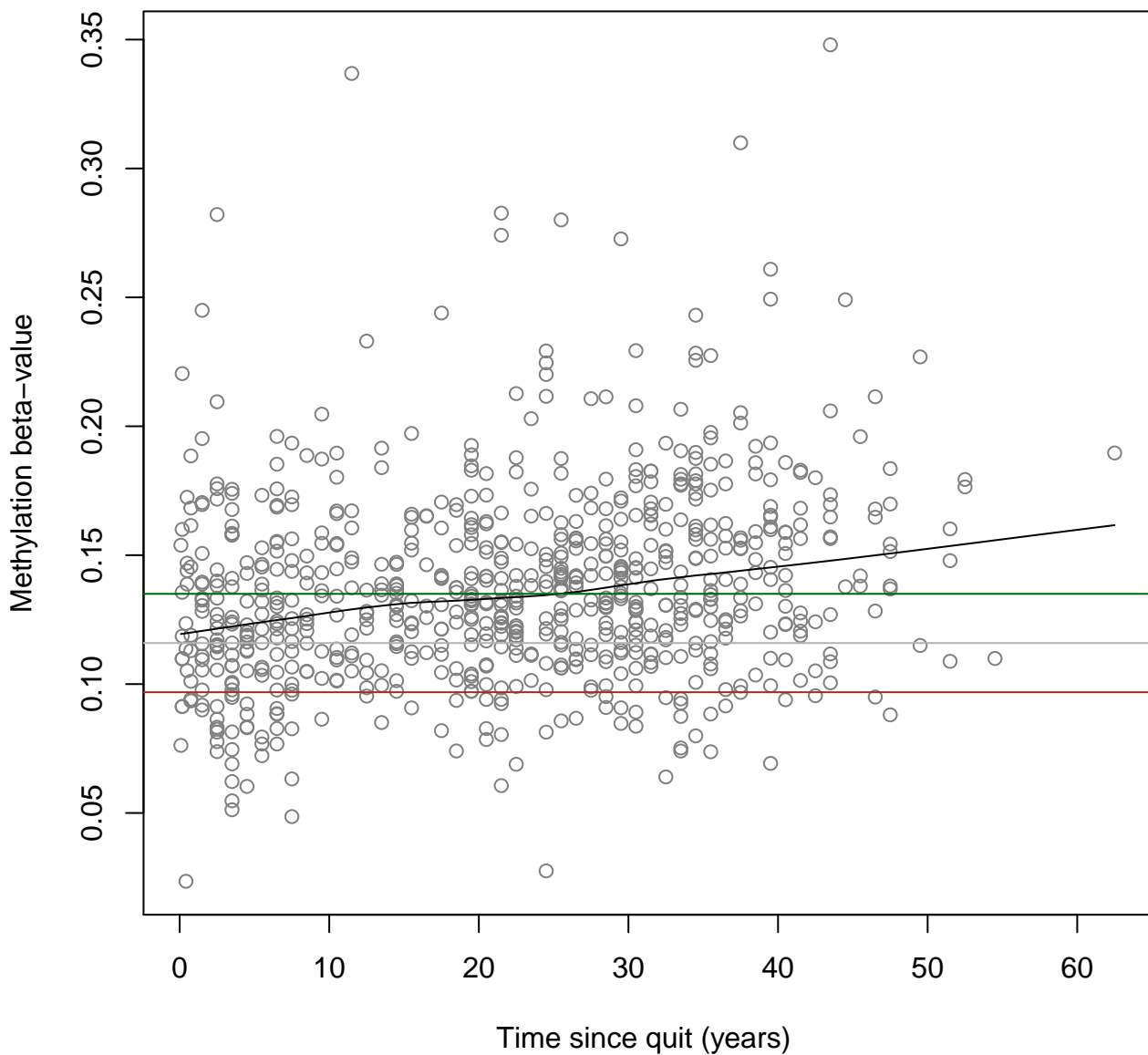

**cg15342087**

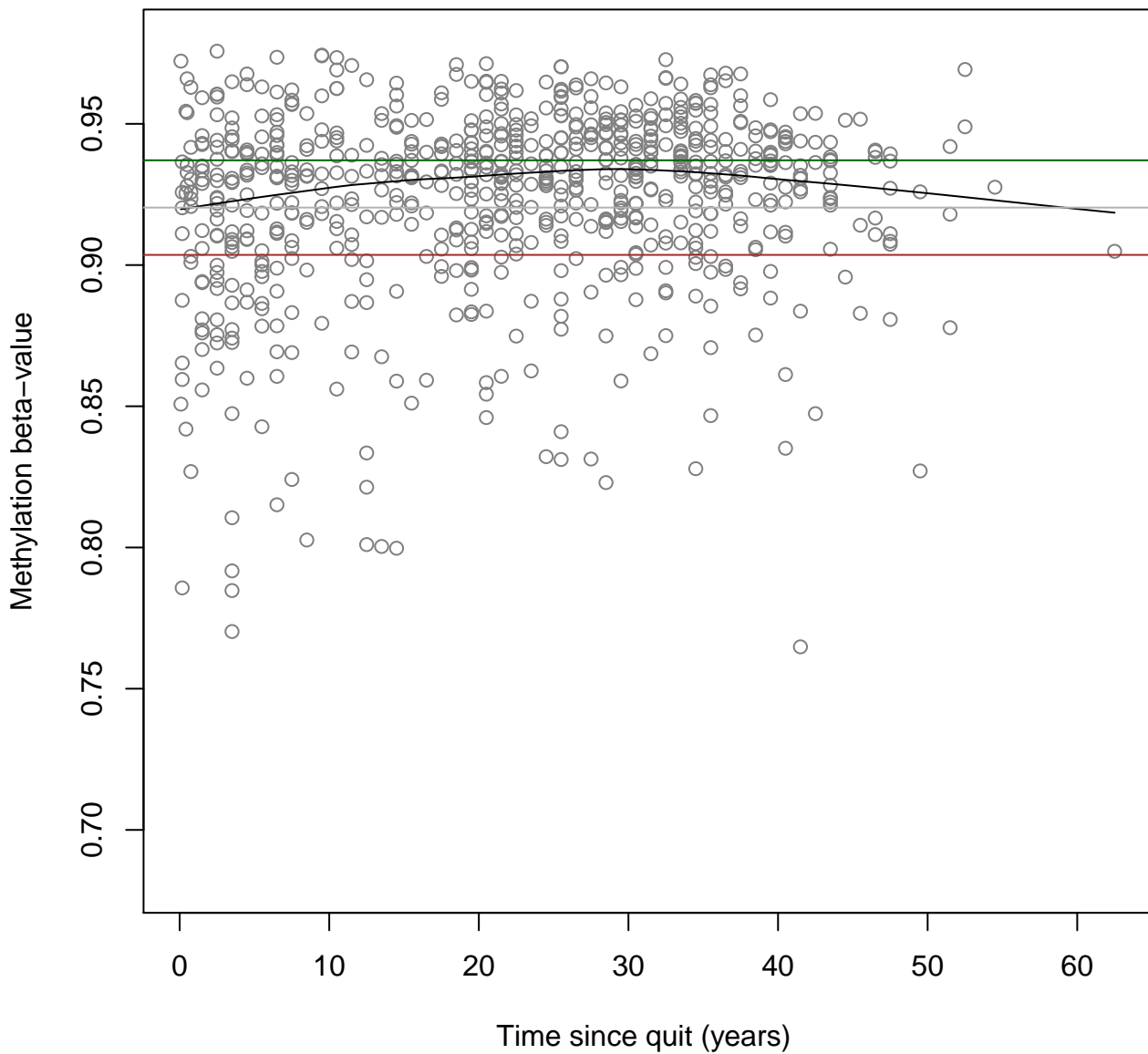

# cg15693572

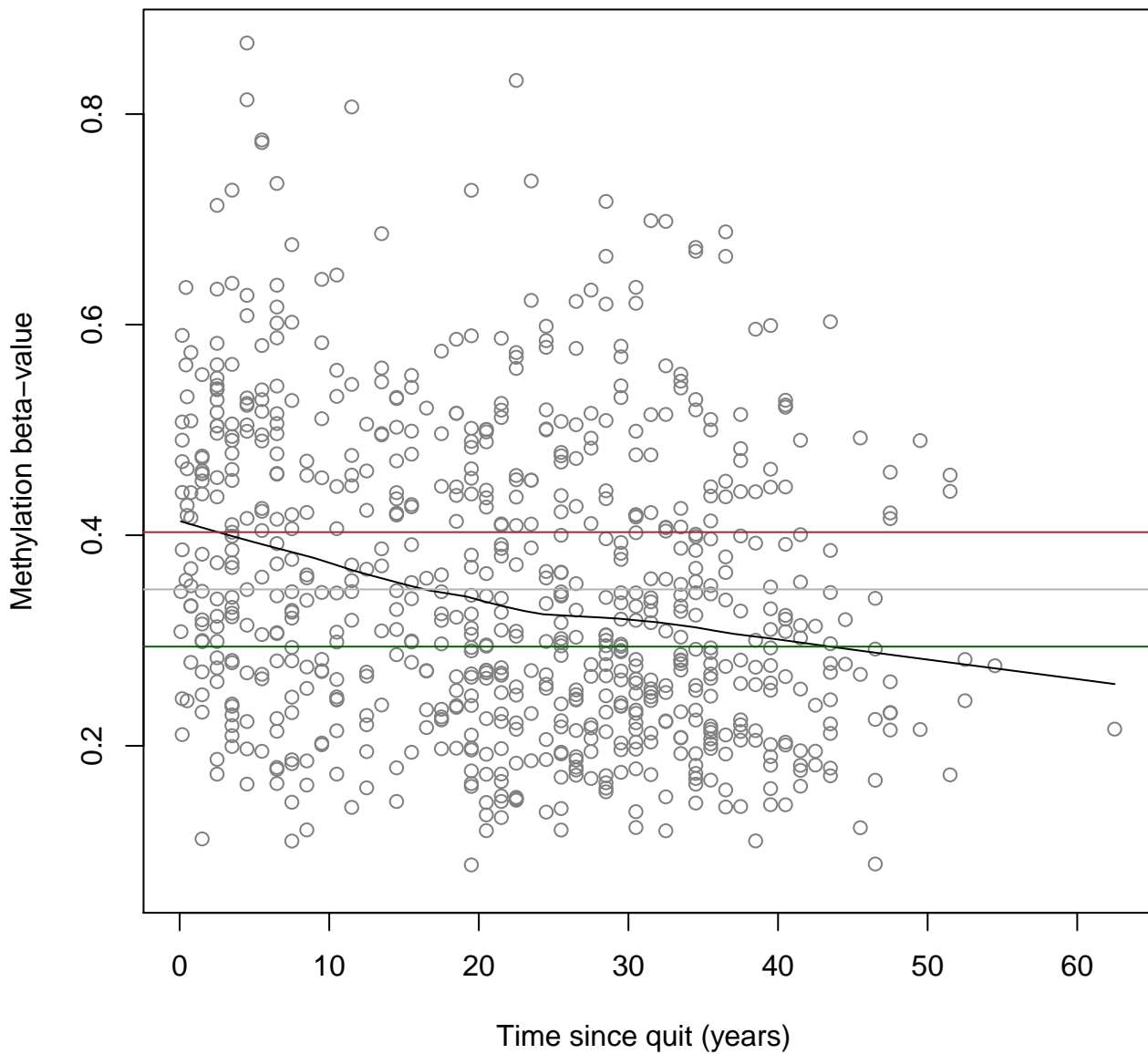

**cg17924476**

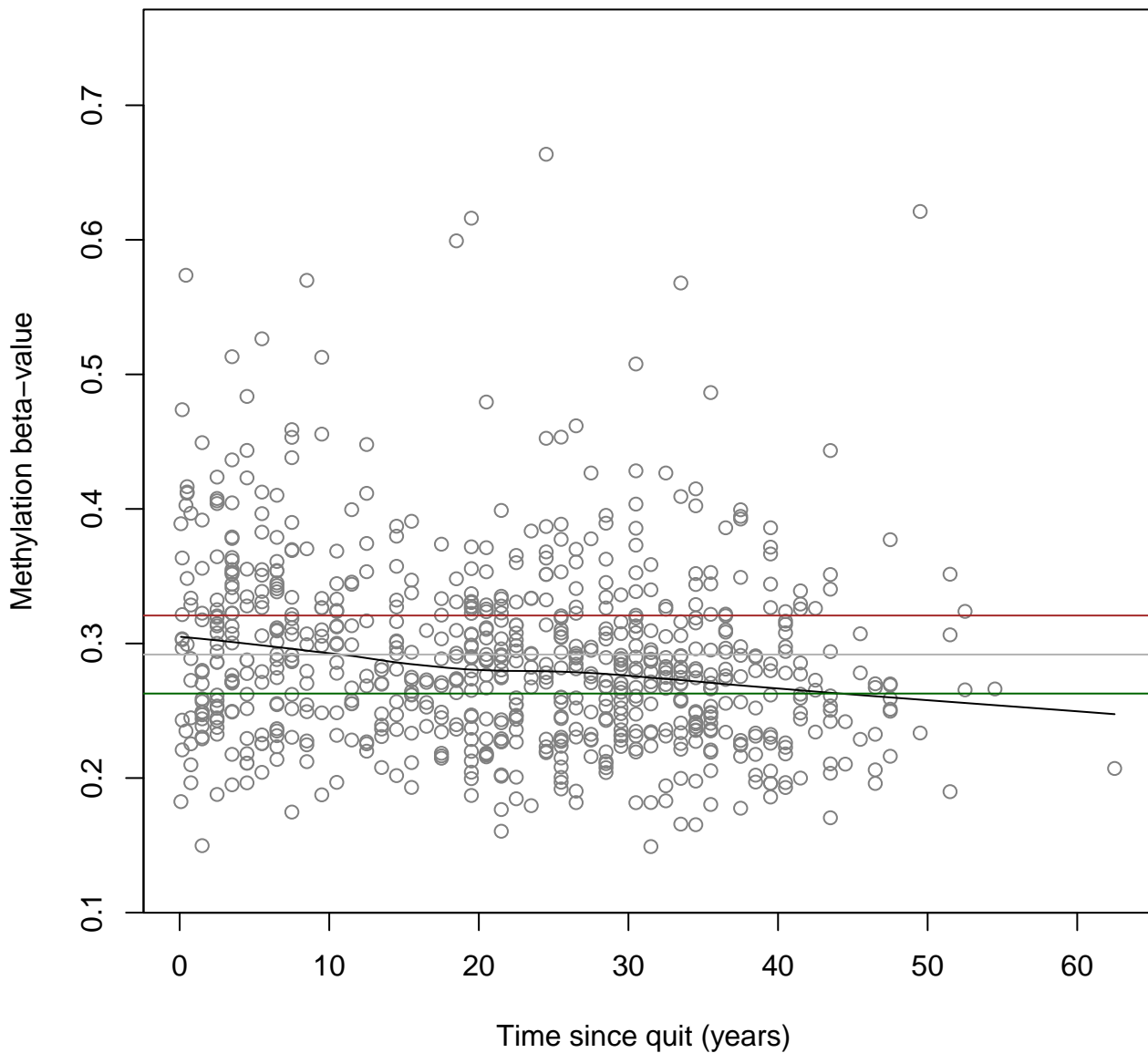

**cg19572487**

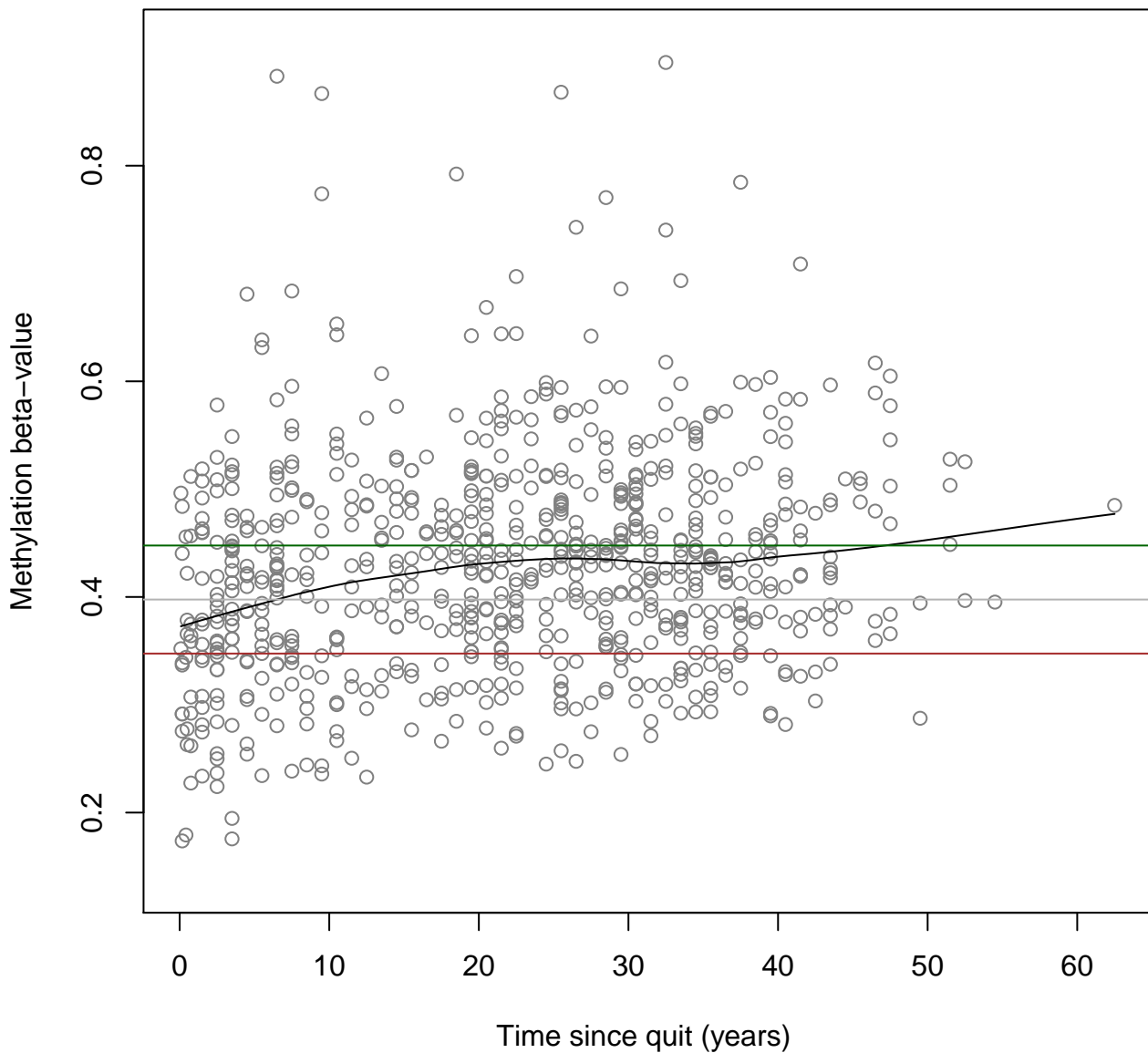

**cg21161138**

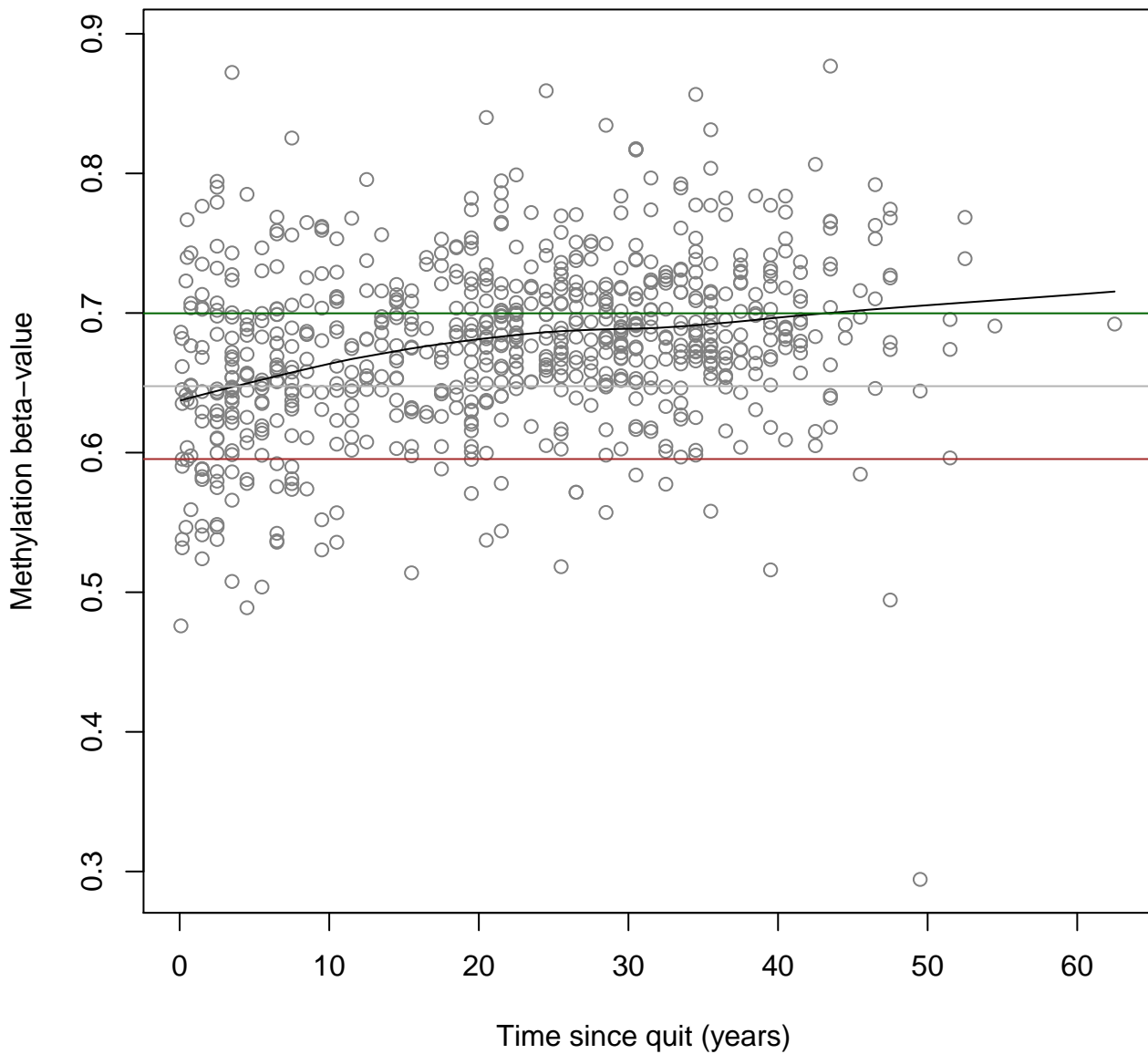

**cg21322436**

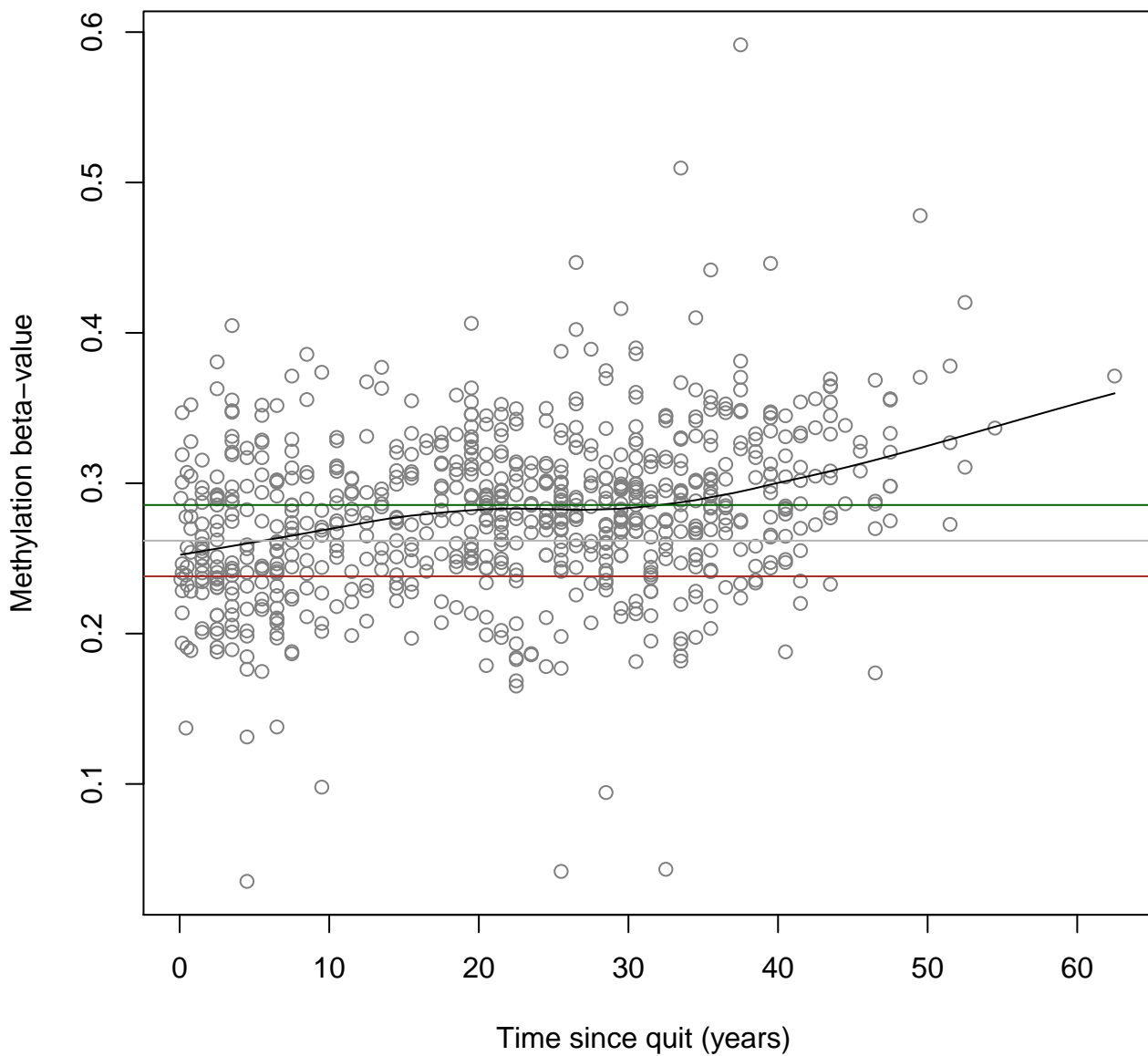

**cg21566642**

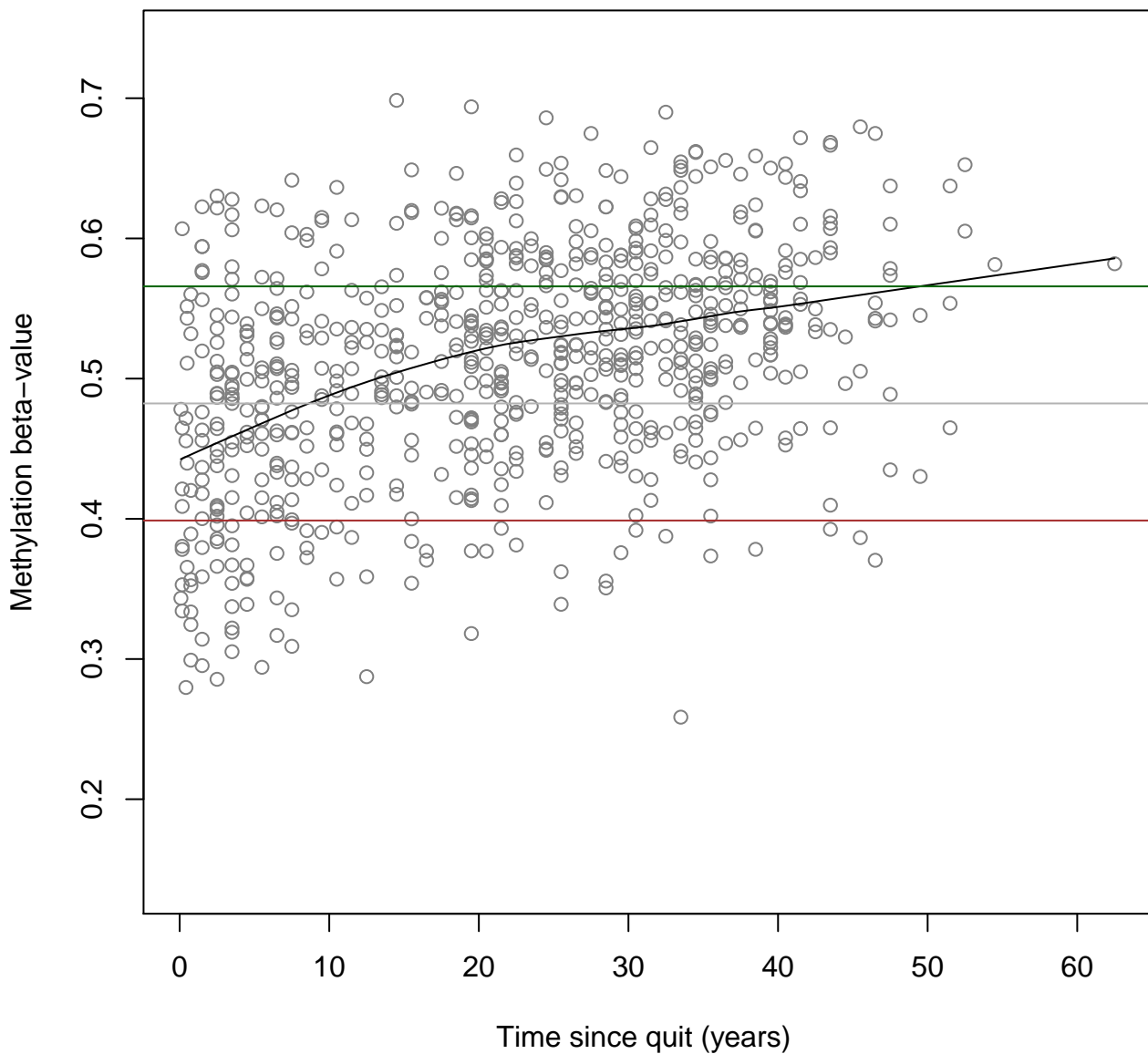

**cg23079012**

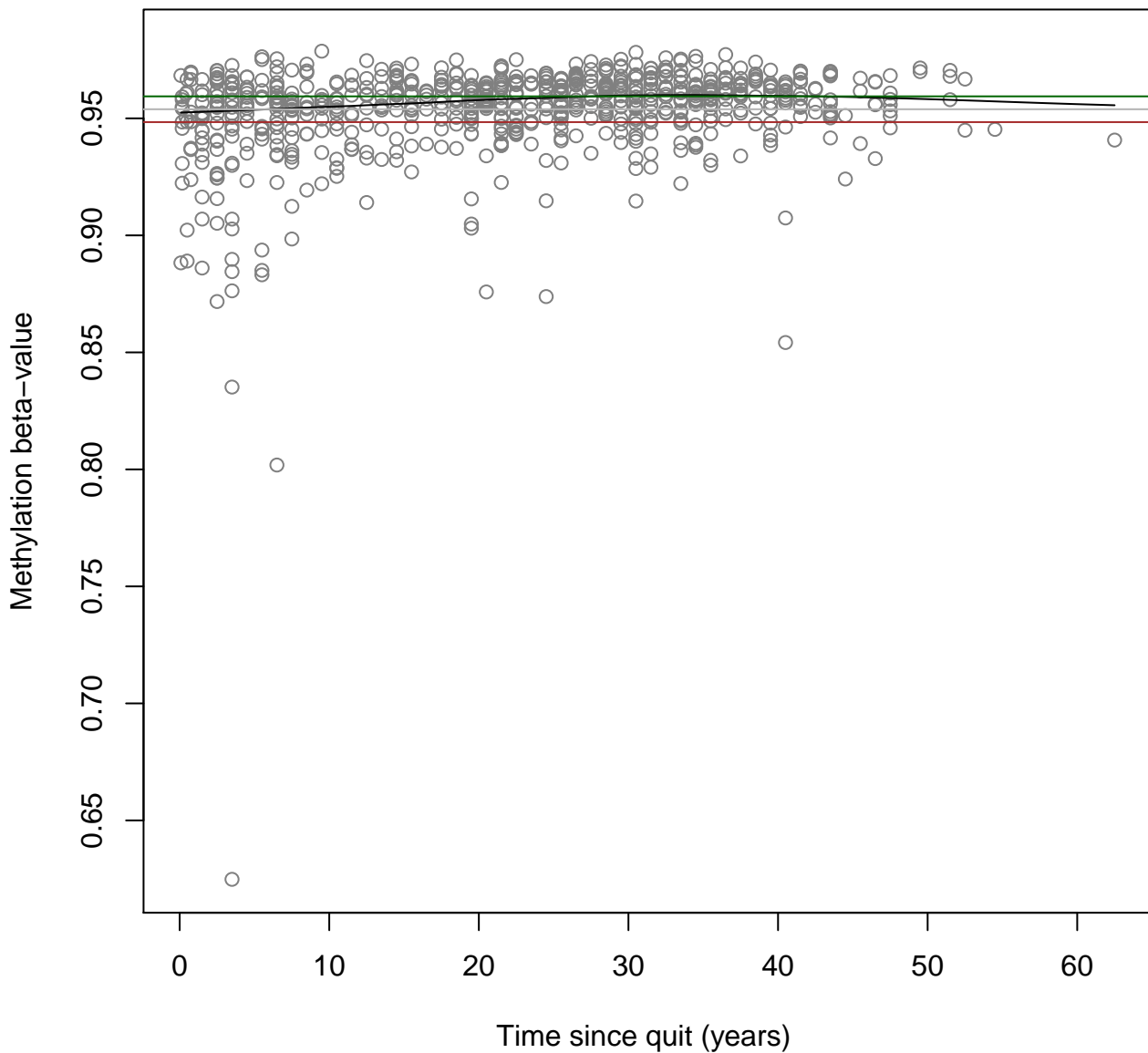

**cg23161492**

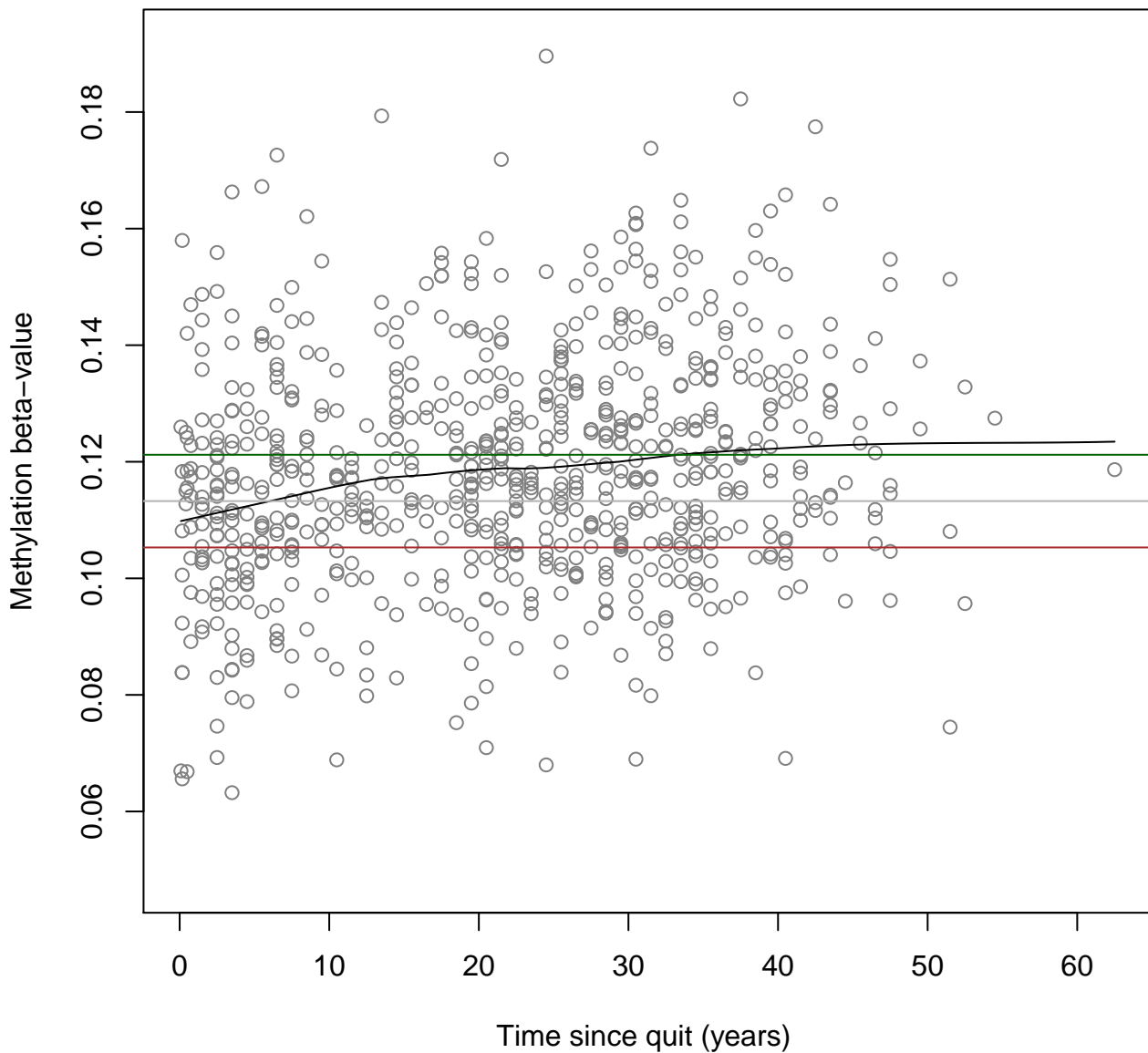

**cg23565821**

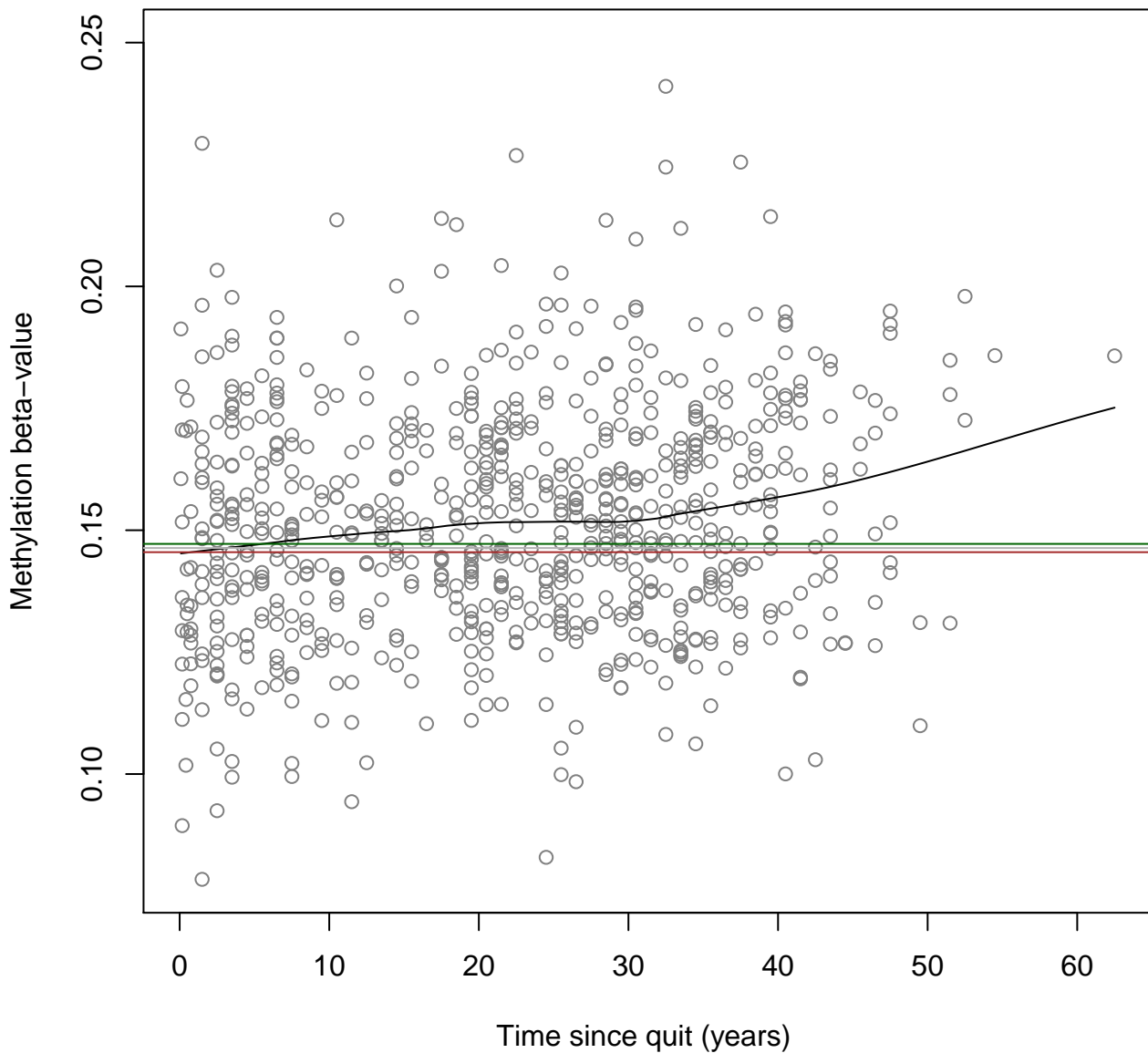

**cg23771366**

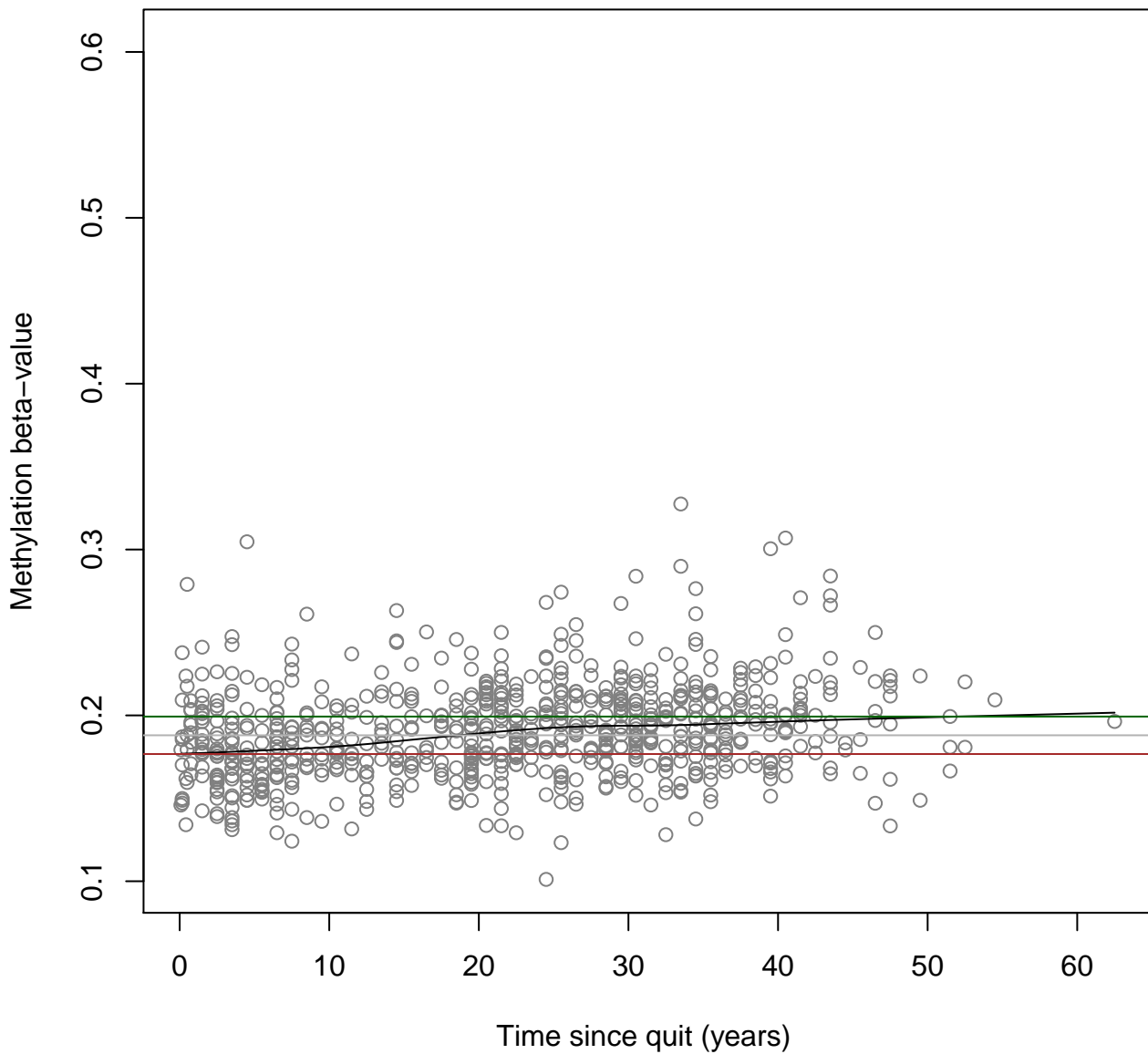

**cg24540678**

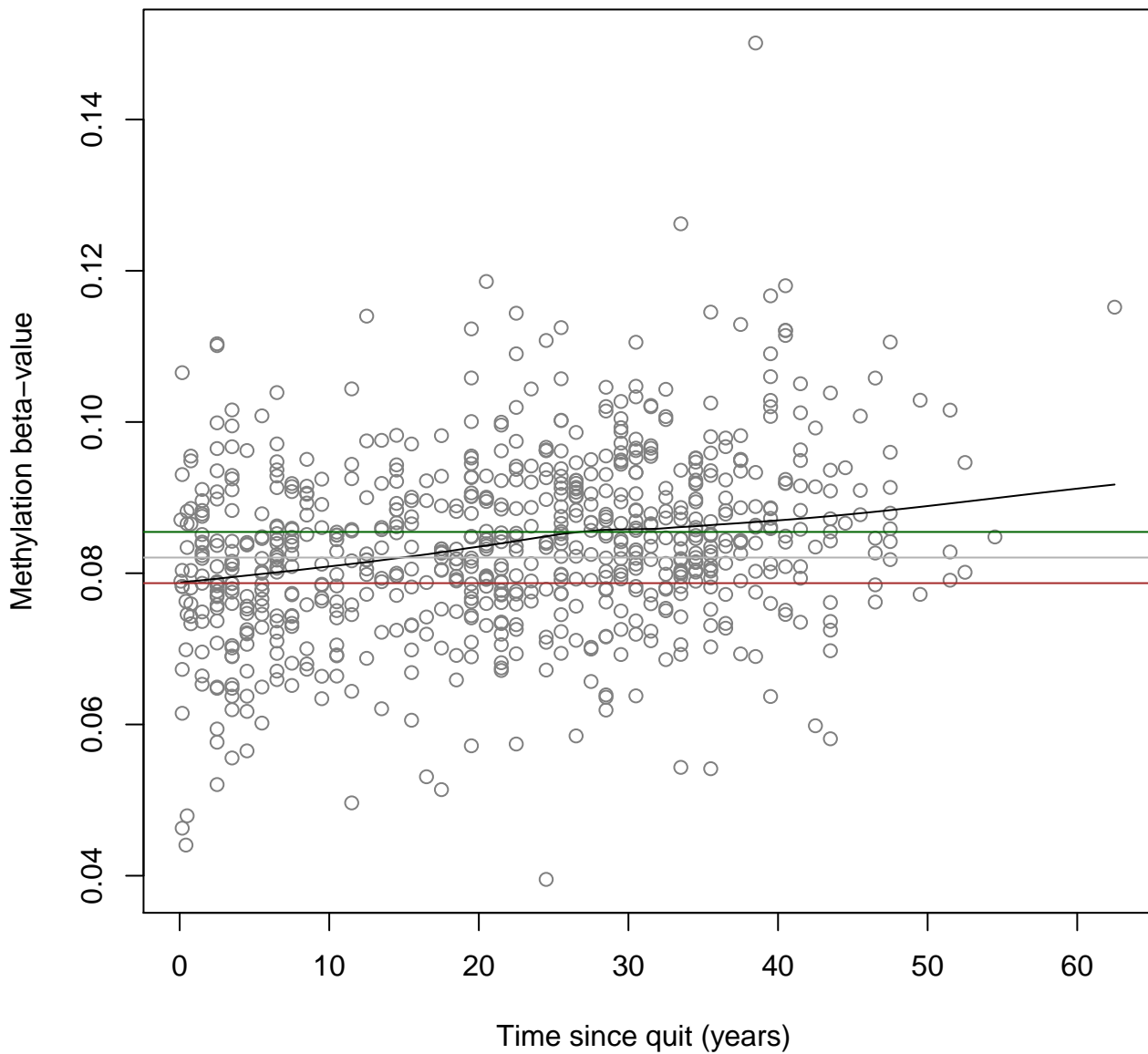

**cg24859433**

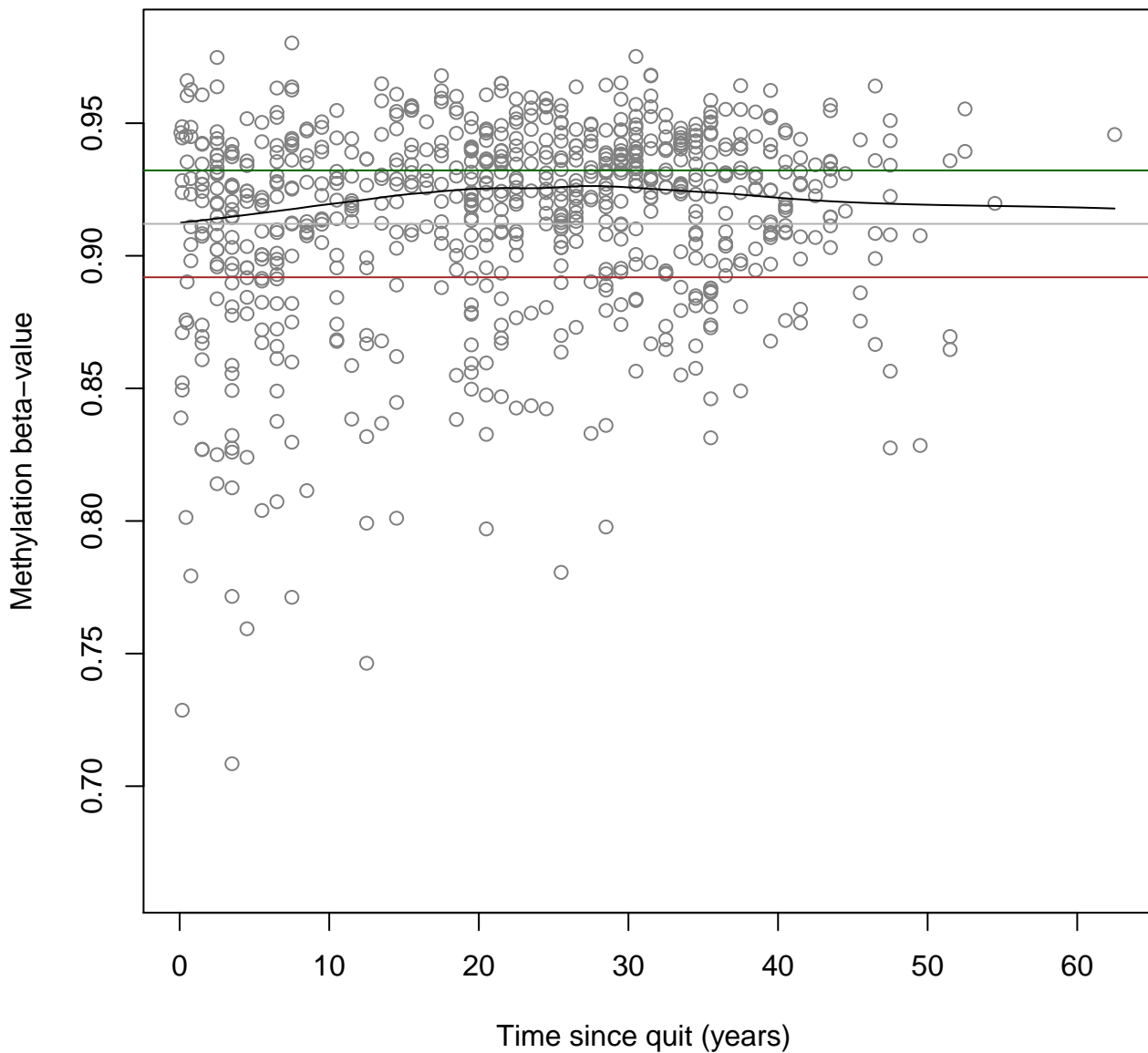

**cg25189904**

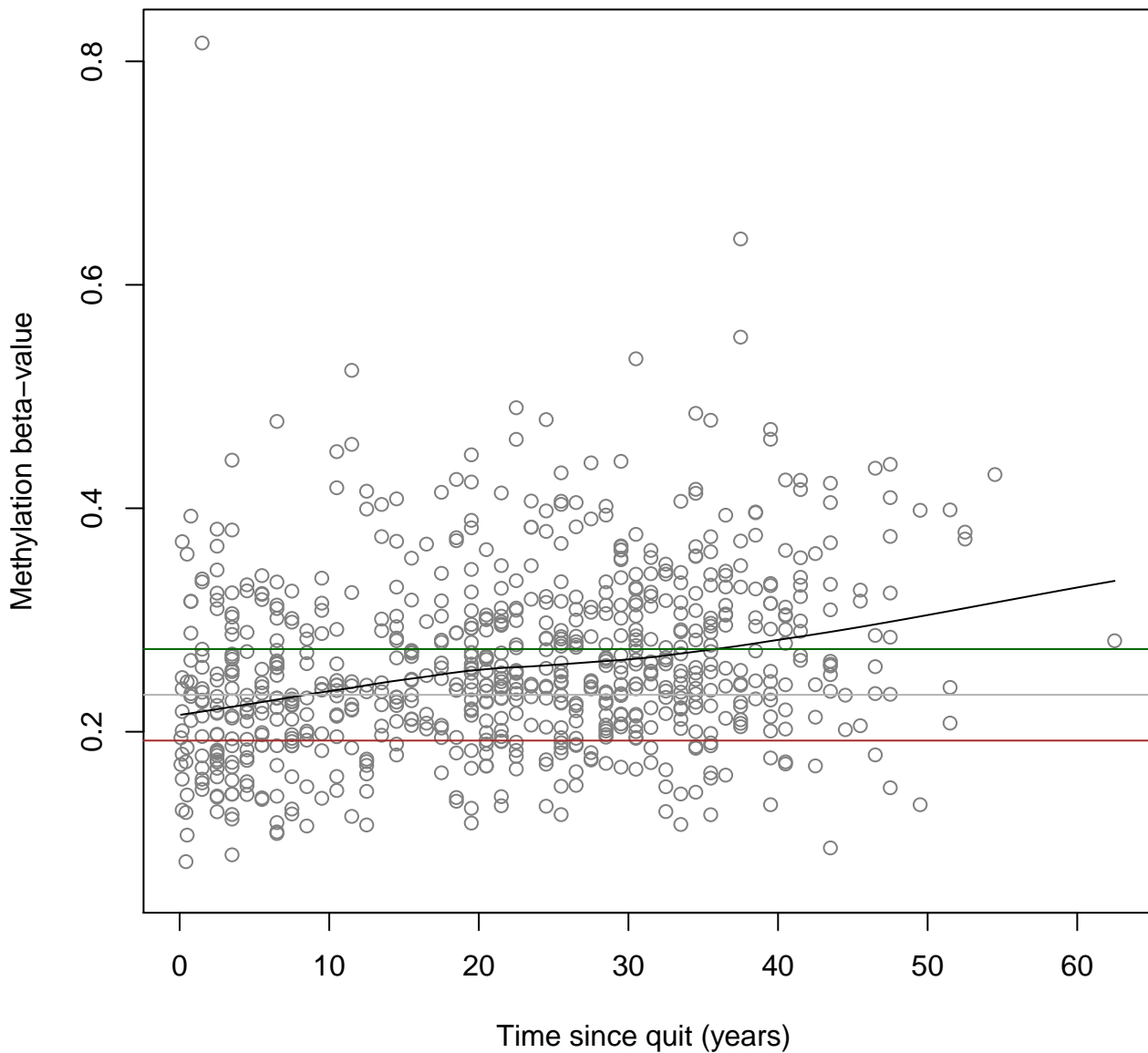

**cg25949550**

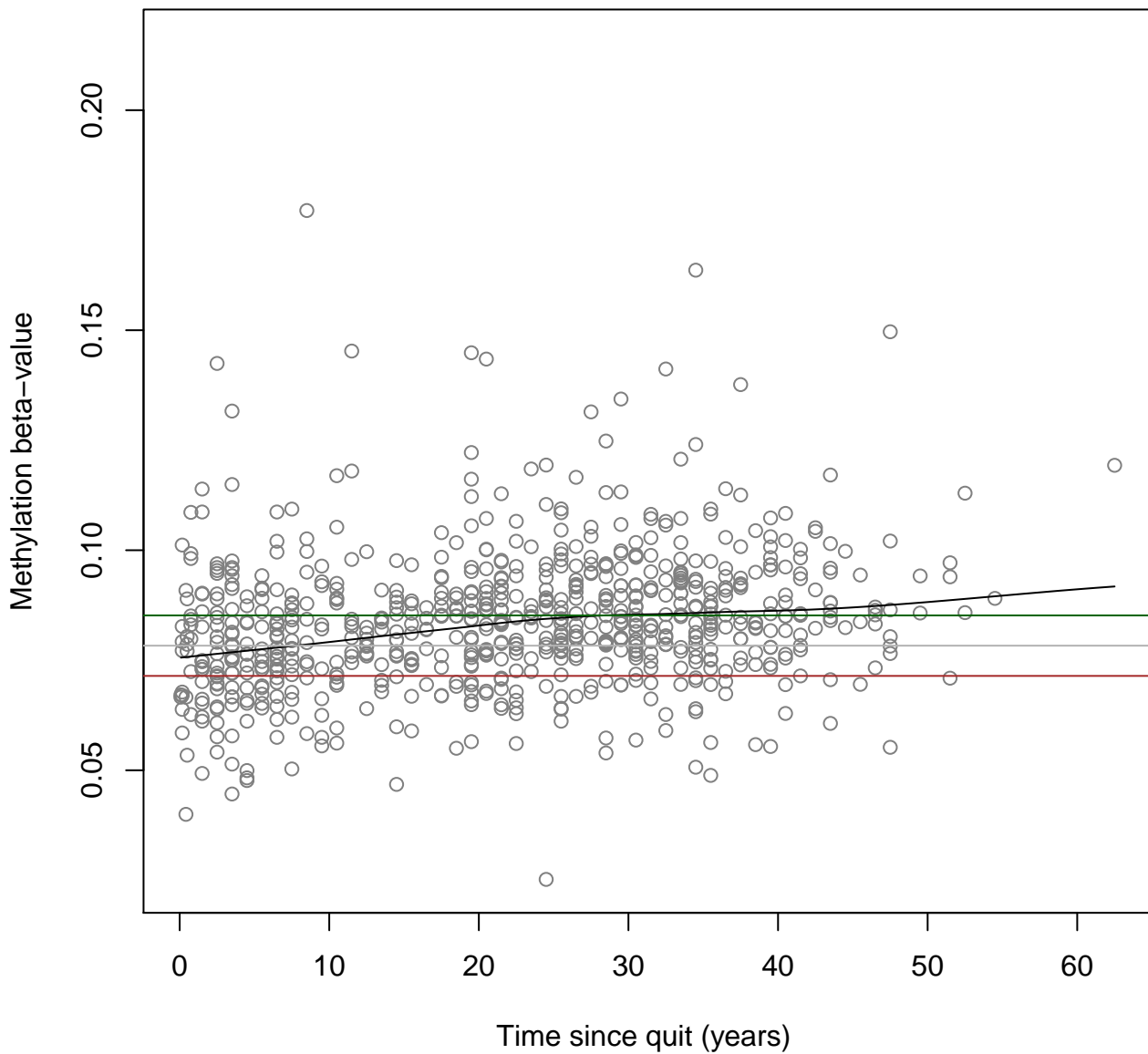

Supplement: Figure S2 — Influence of time since quitting on the DNA methylation state in former smokers. Illustrated by a loess curve in the scatterplots are the years needed for former smokers to acquire a median ß-value methylation state at single CpG sites that is closer to or equals the one of never smokers; the x-axis displays the cessation time in years, the y-axis displays the methylation level with the use of numbers between 0 (for 0% methylation) and 1 (for 100% methylation); horizontal brown line: median methylation level of current smokers; horizontal green line: median methylation level of never smokers; horizontal grey line: center line of current and never smokers median ß-value methylation; please see Table 4 for detailed data. (PDF) [file pone.0063812.s002.pdf]

**cg01940273**

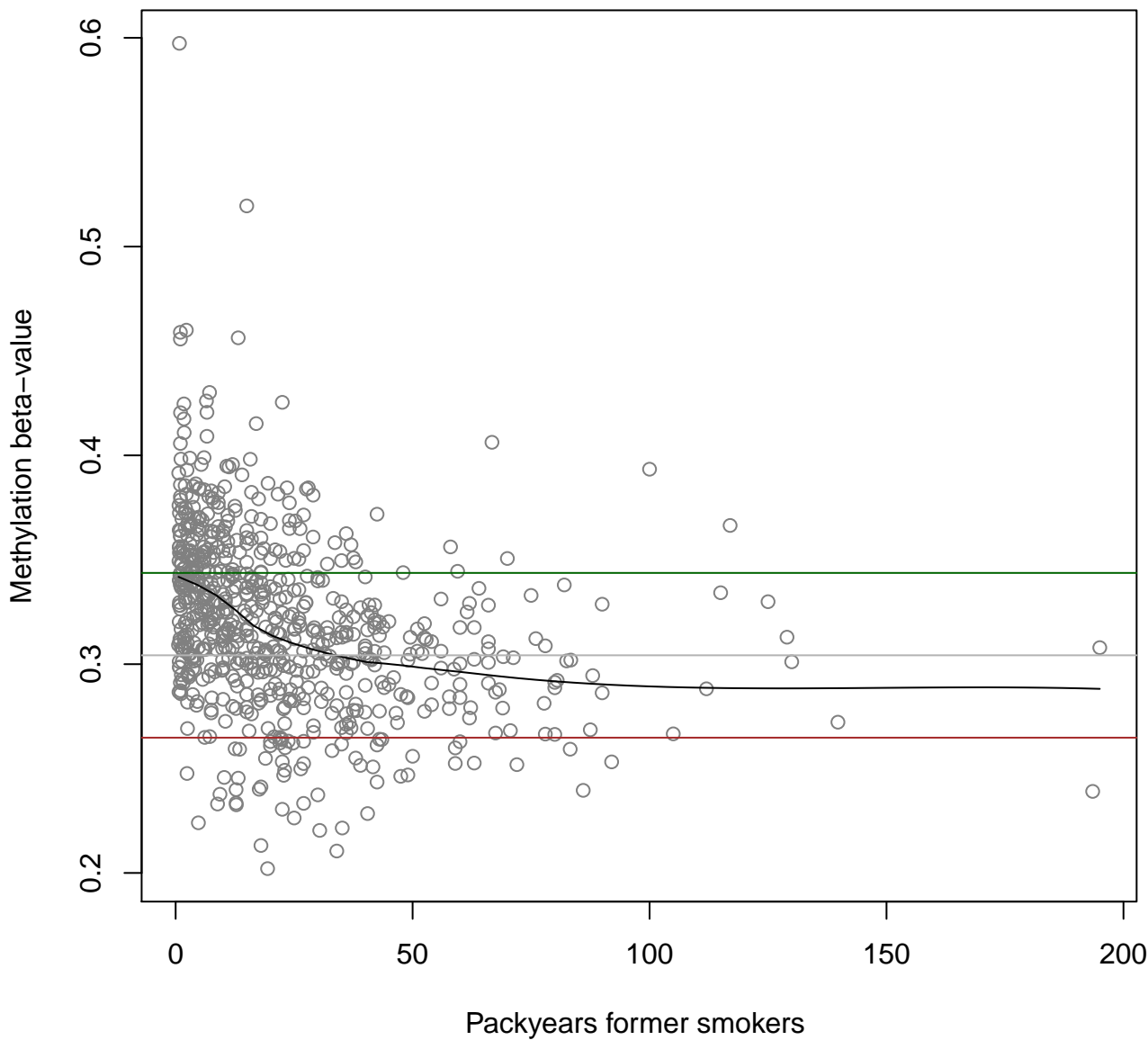

**cg03636183**

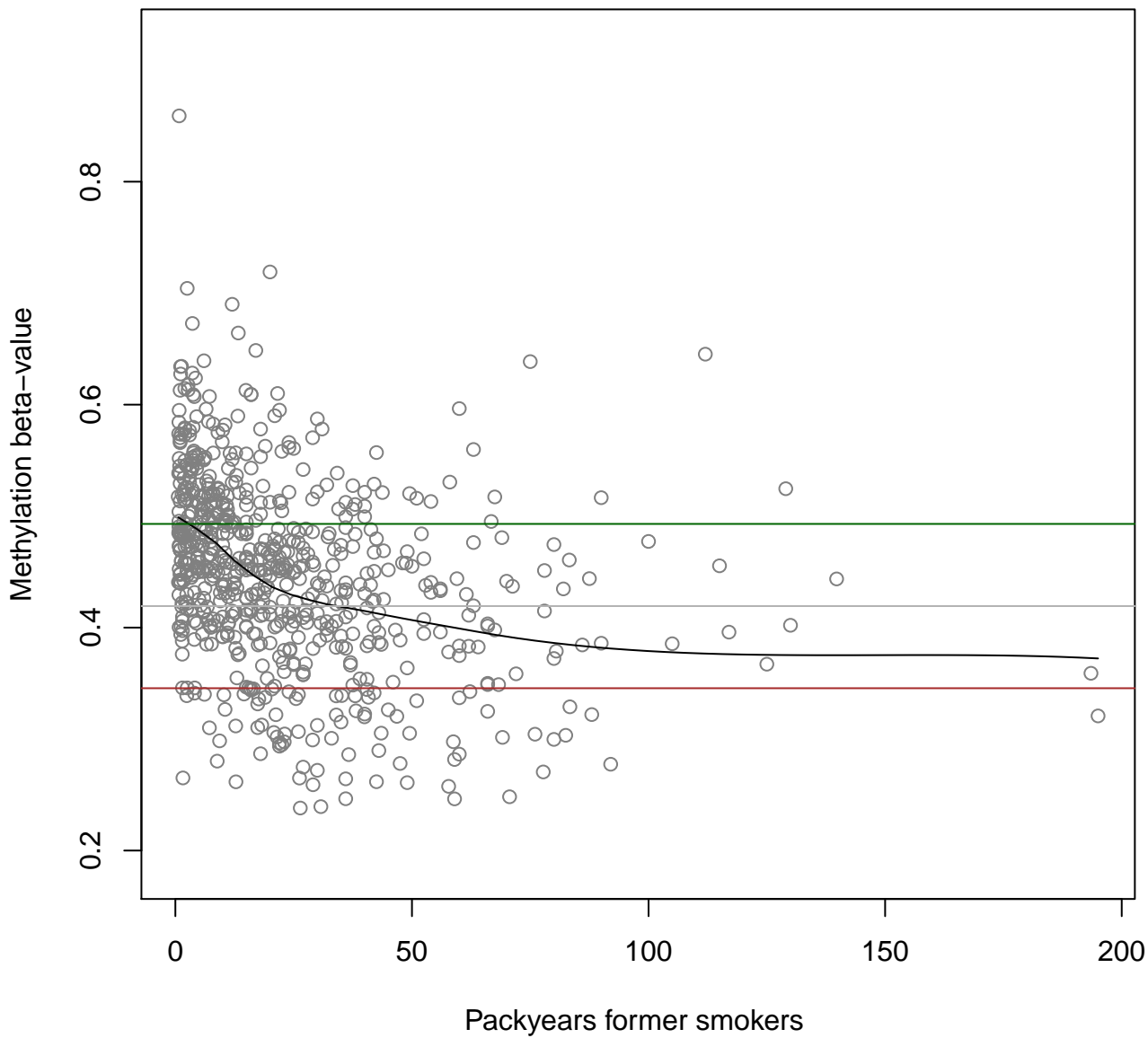

**cg05575921**

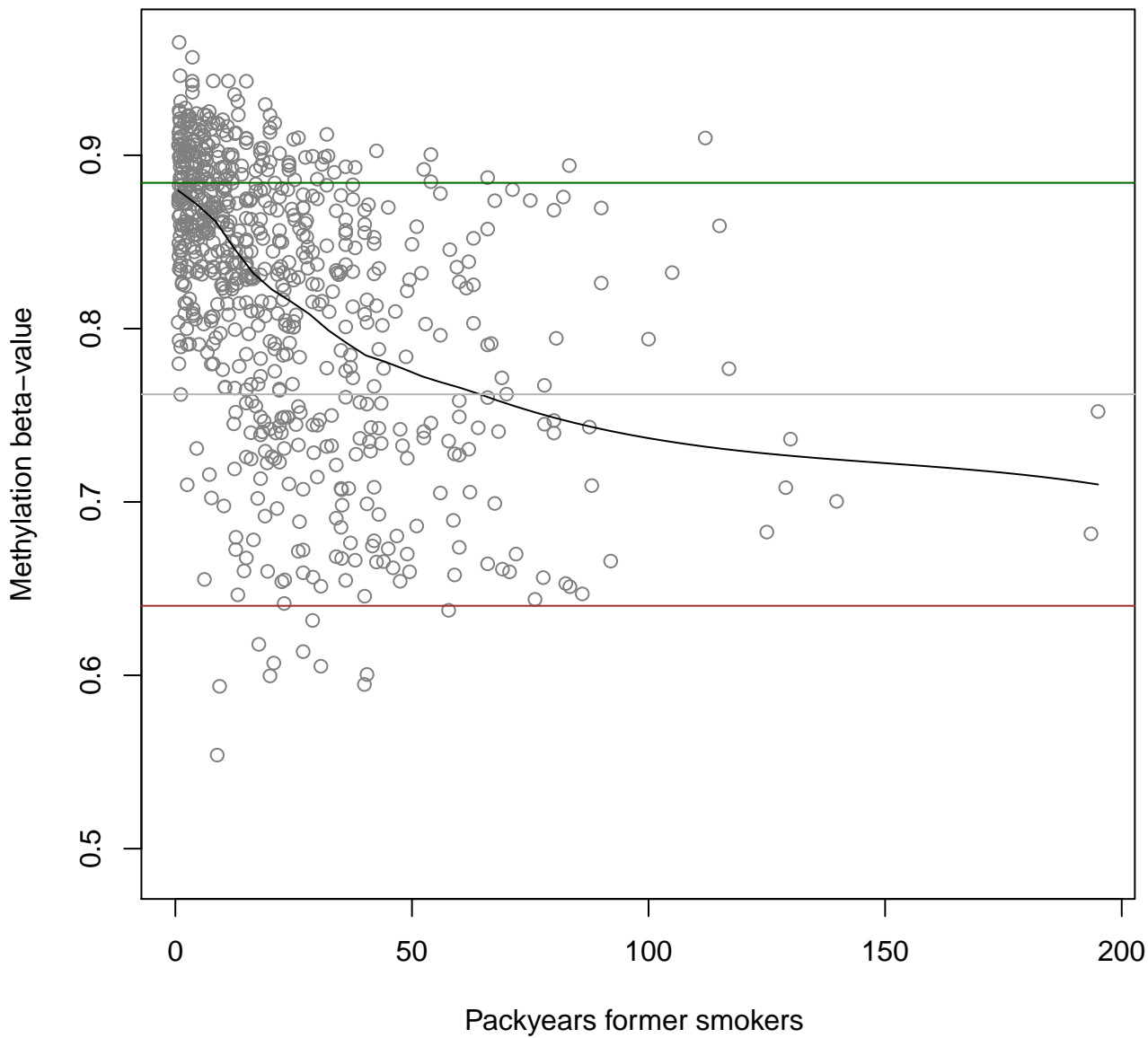

**cg05951221**

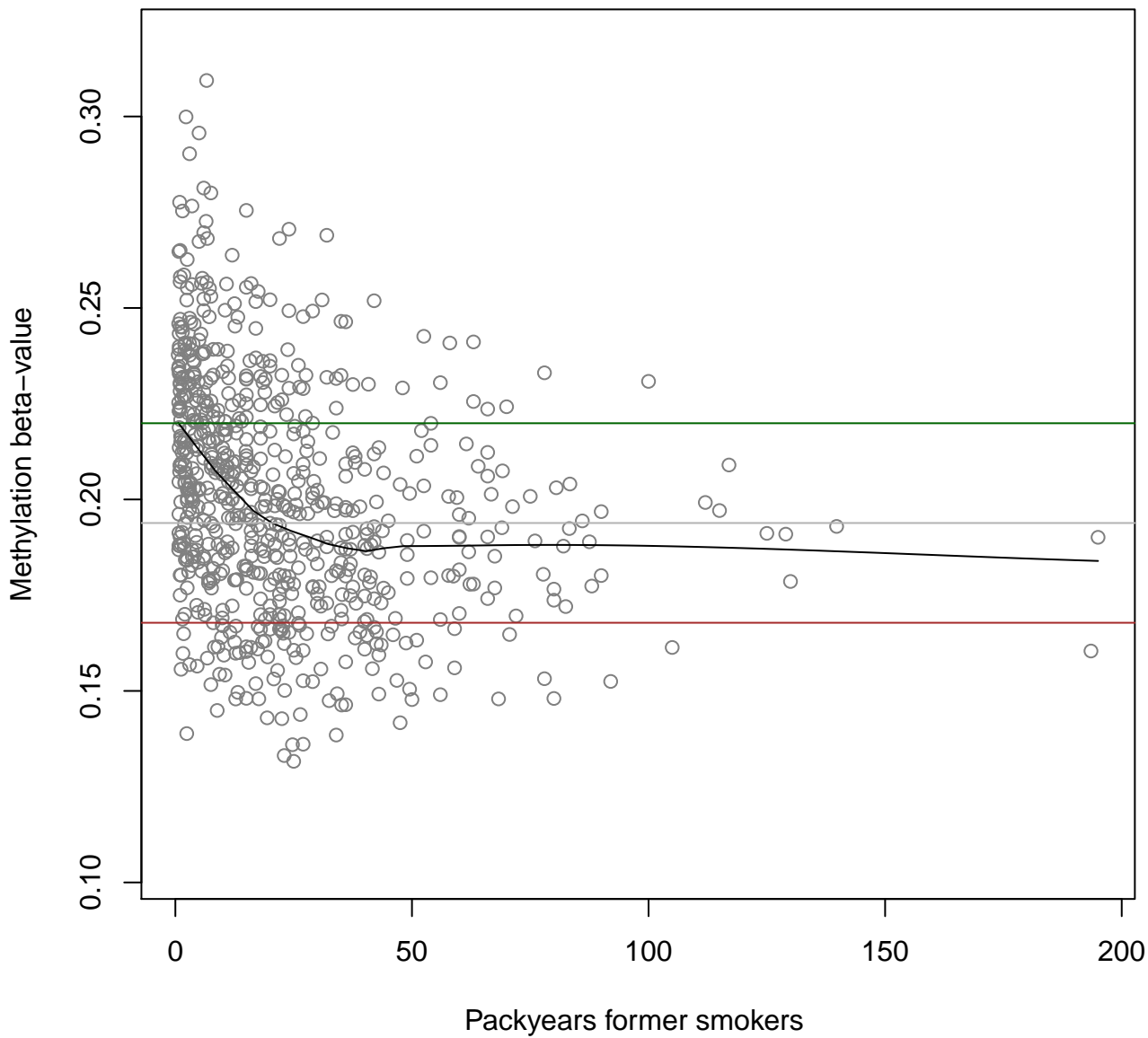

**cg06126421**

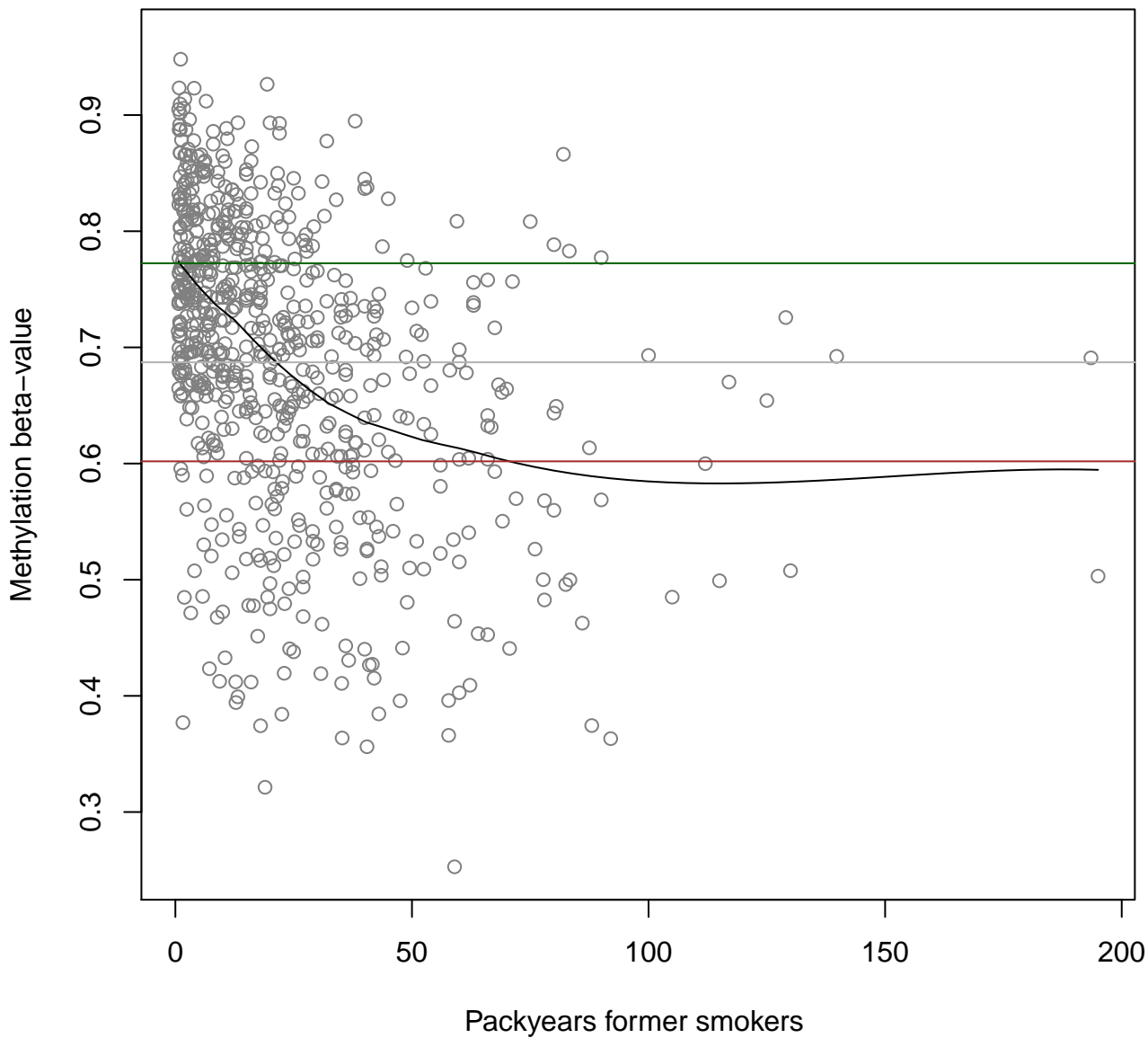

**cg06644428**

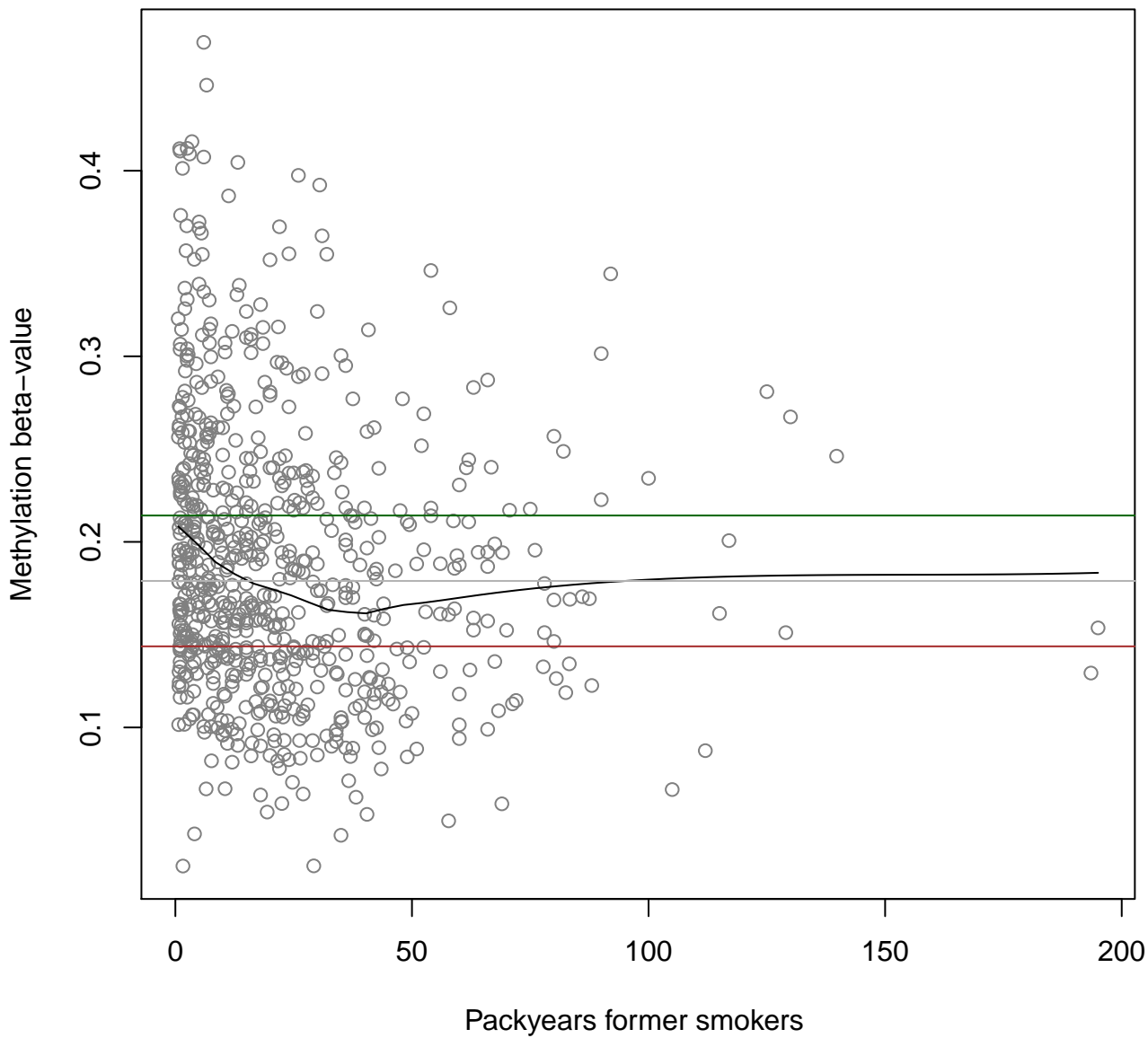

**cg11554391**

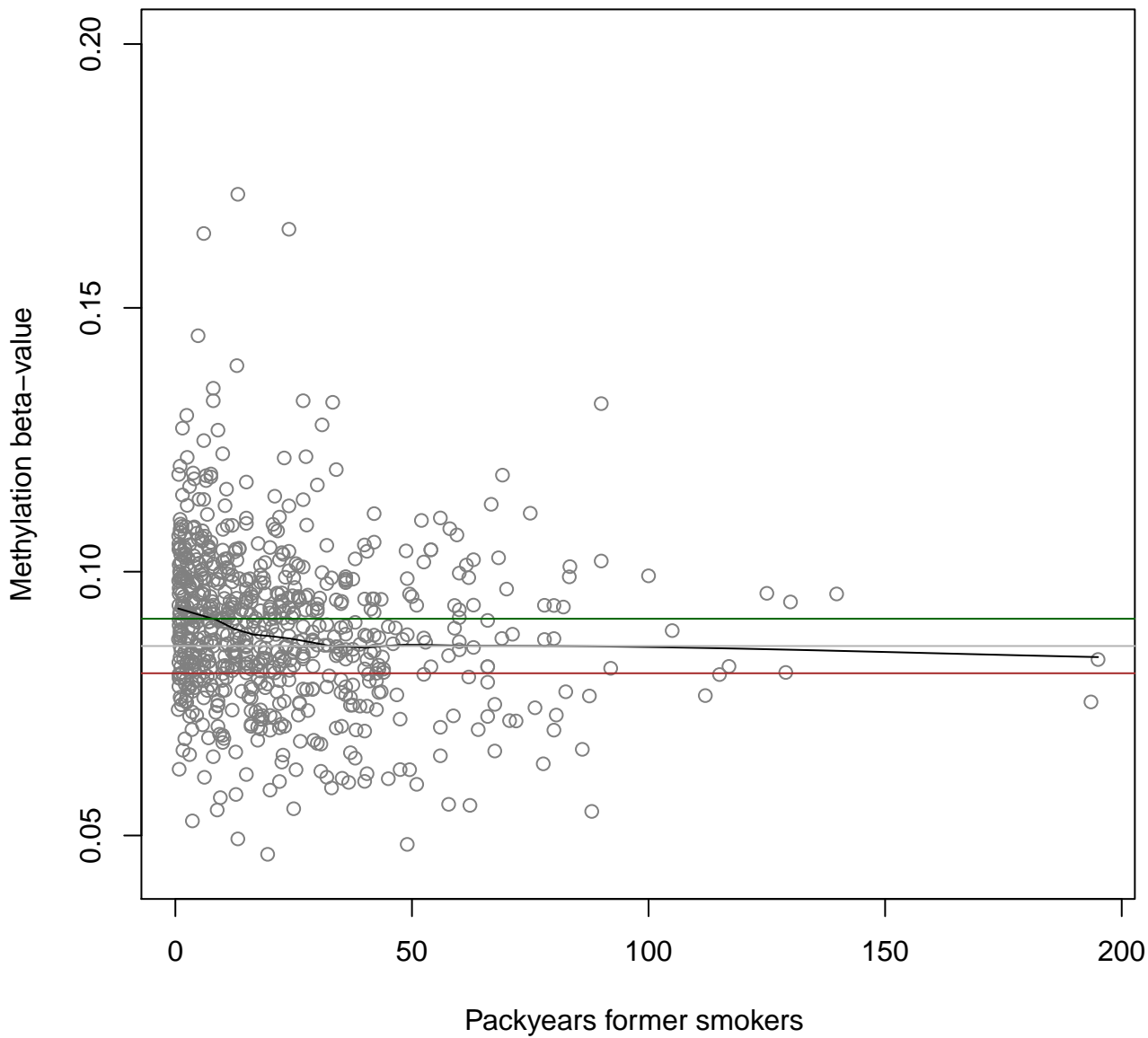

**cg11660018**

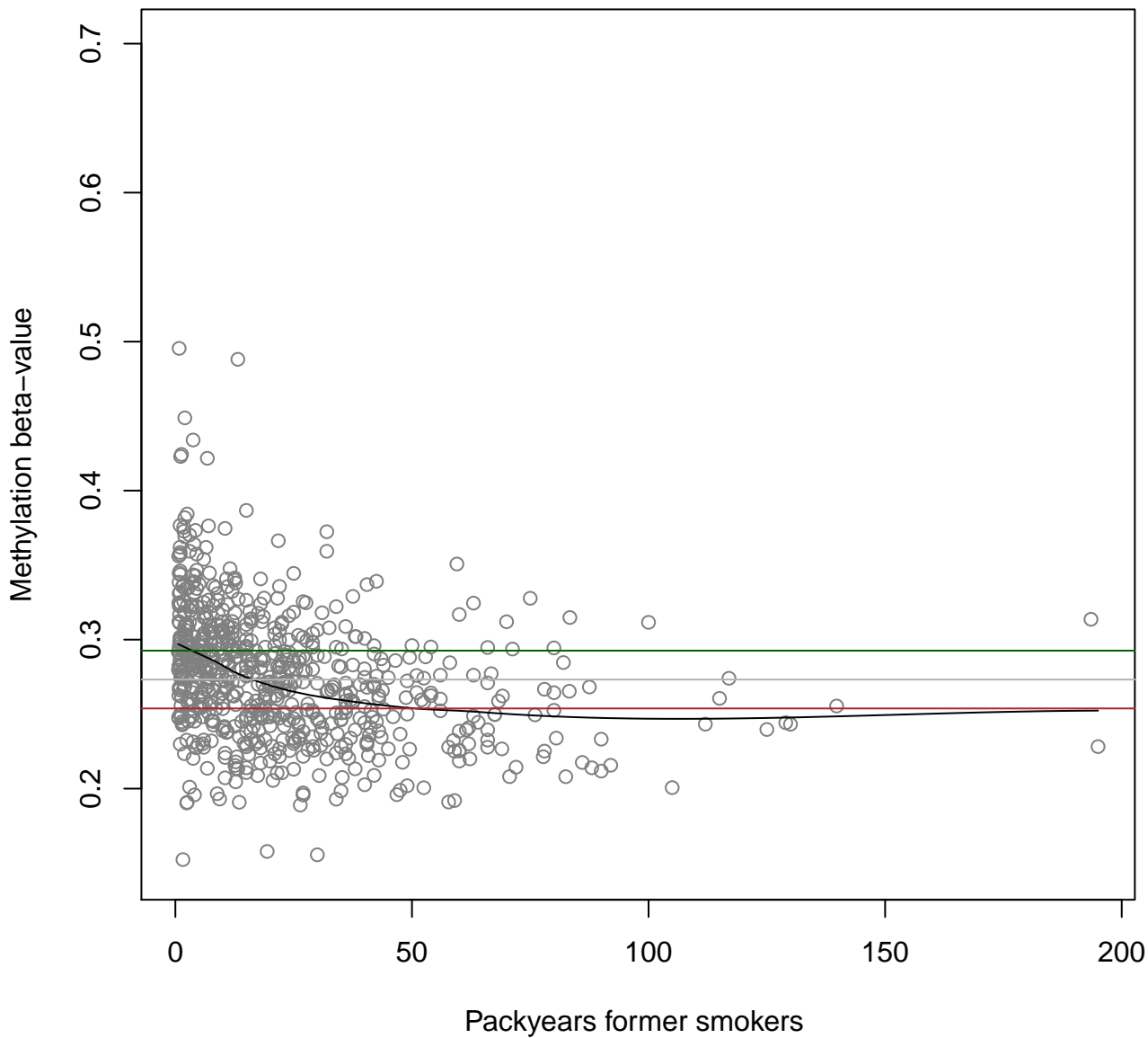

**cg14753356**

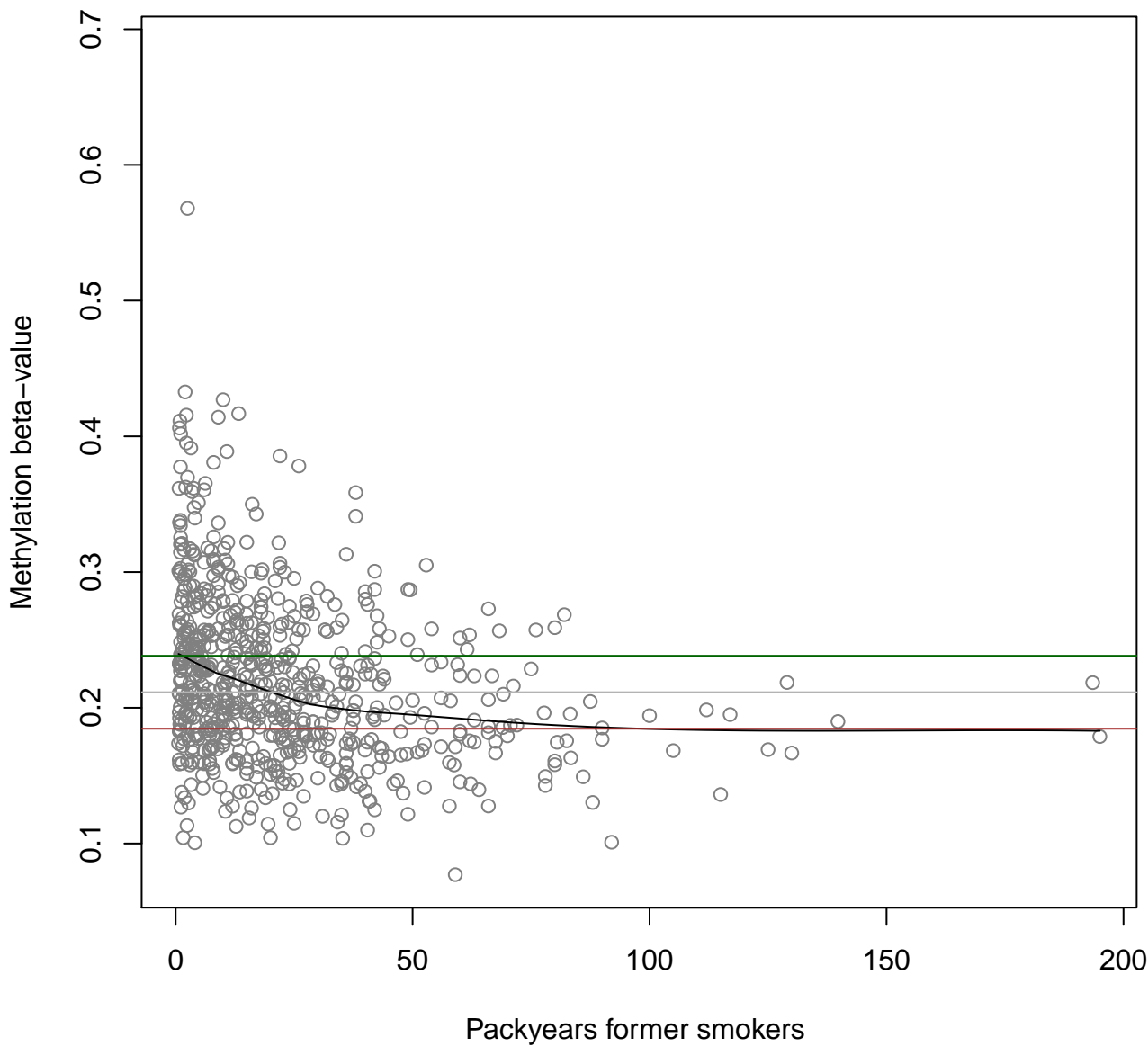

**cg21161138**

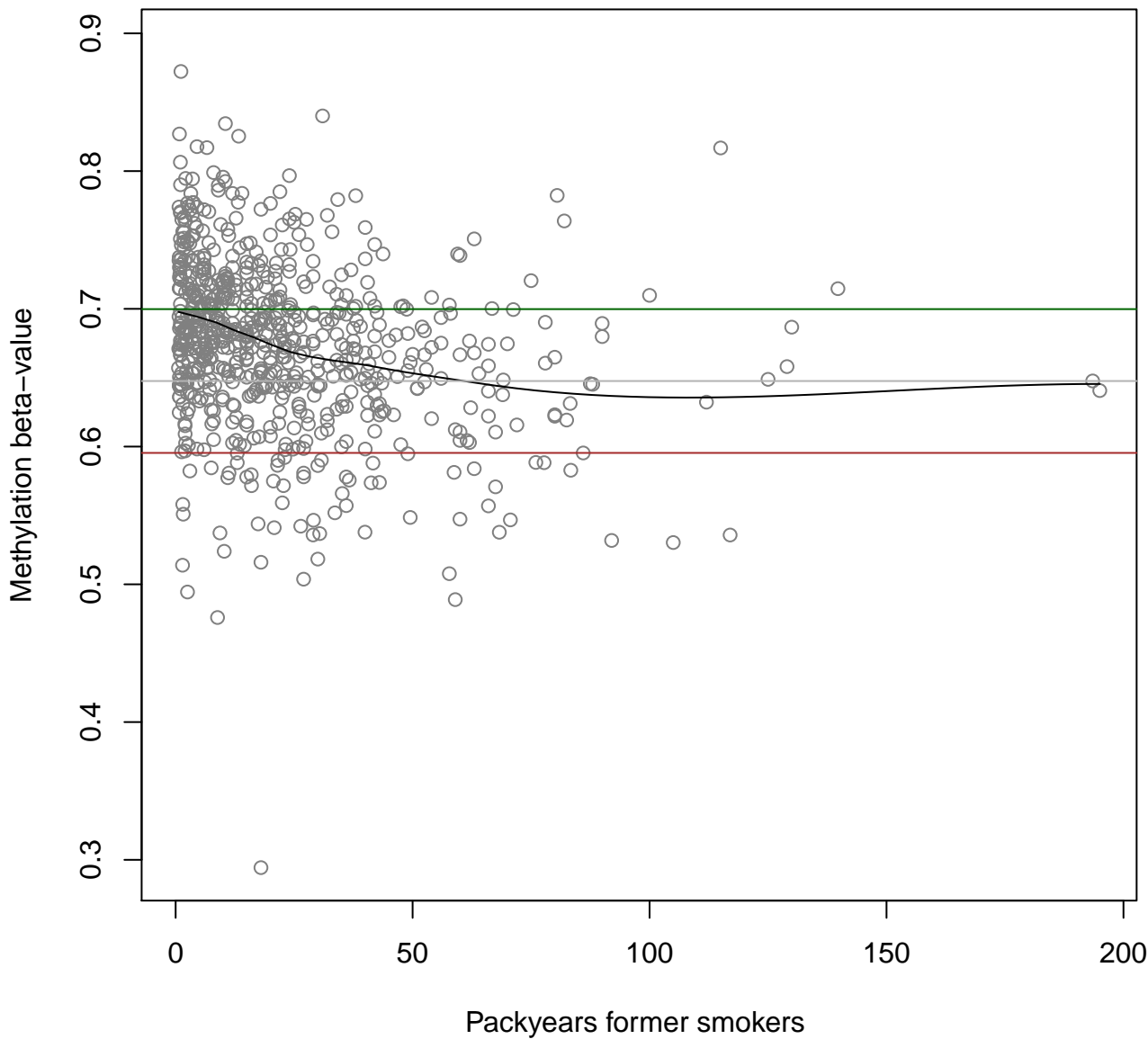

**cg21566642**

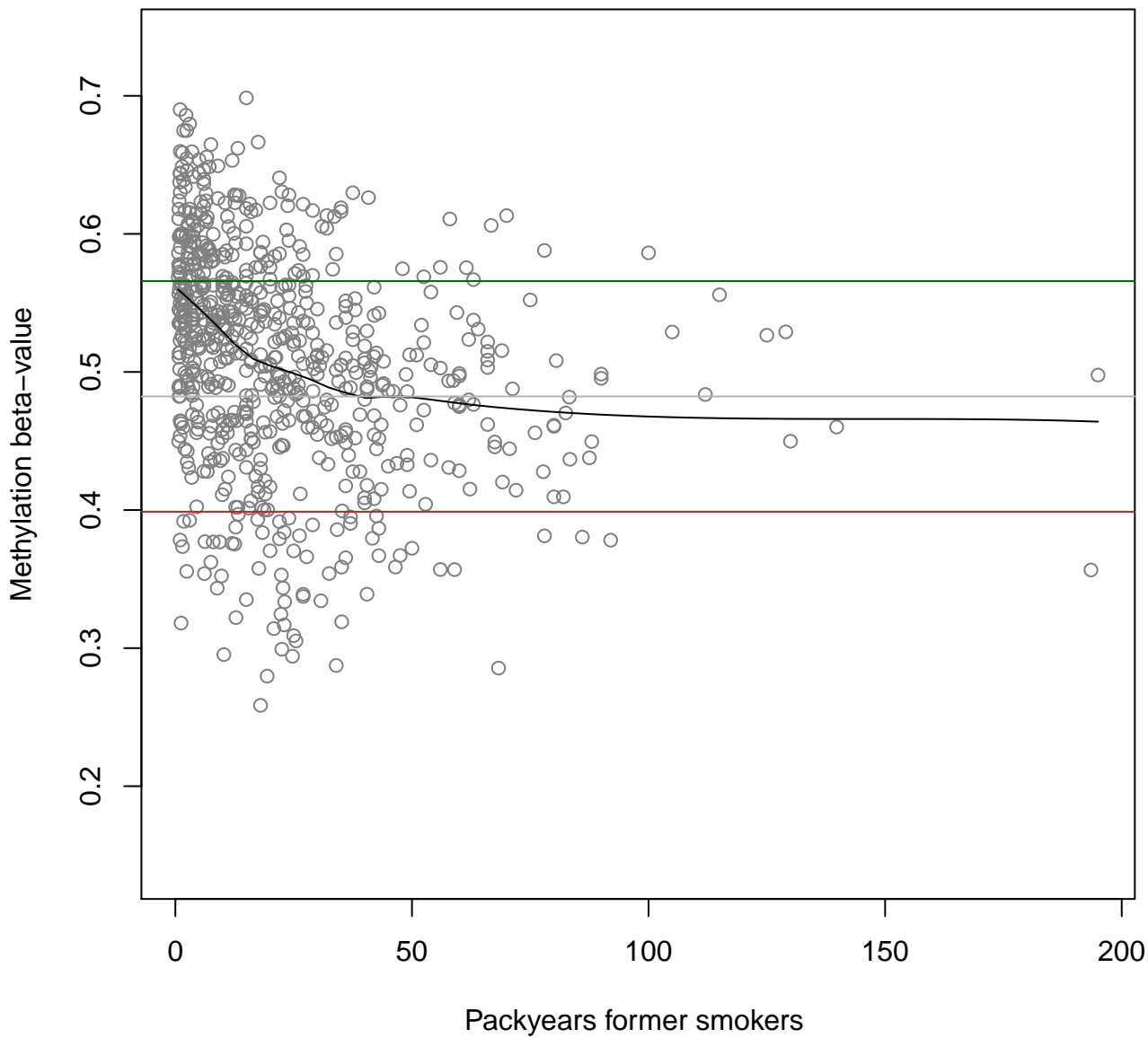

**cg23079012**

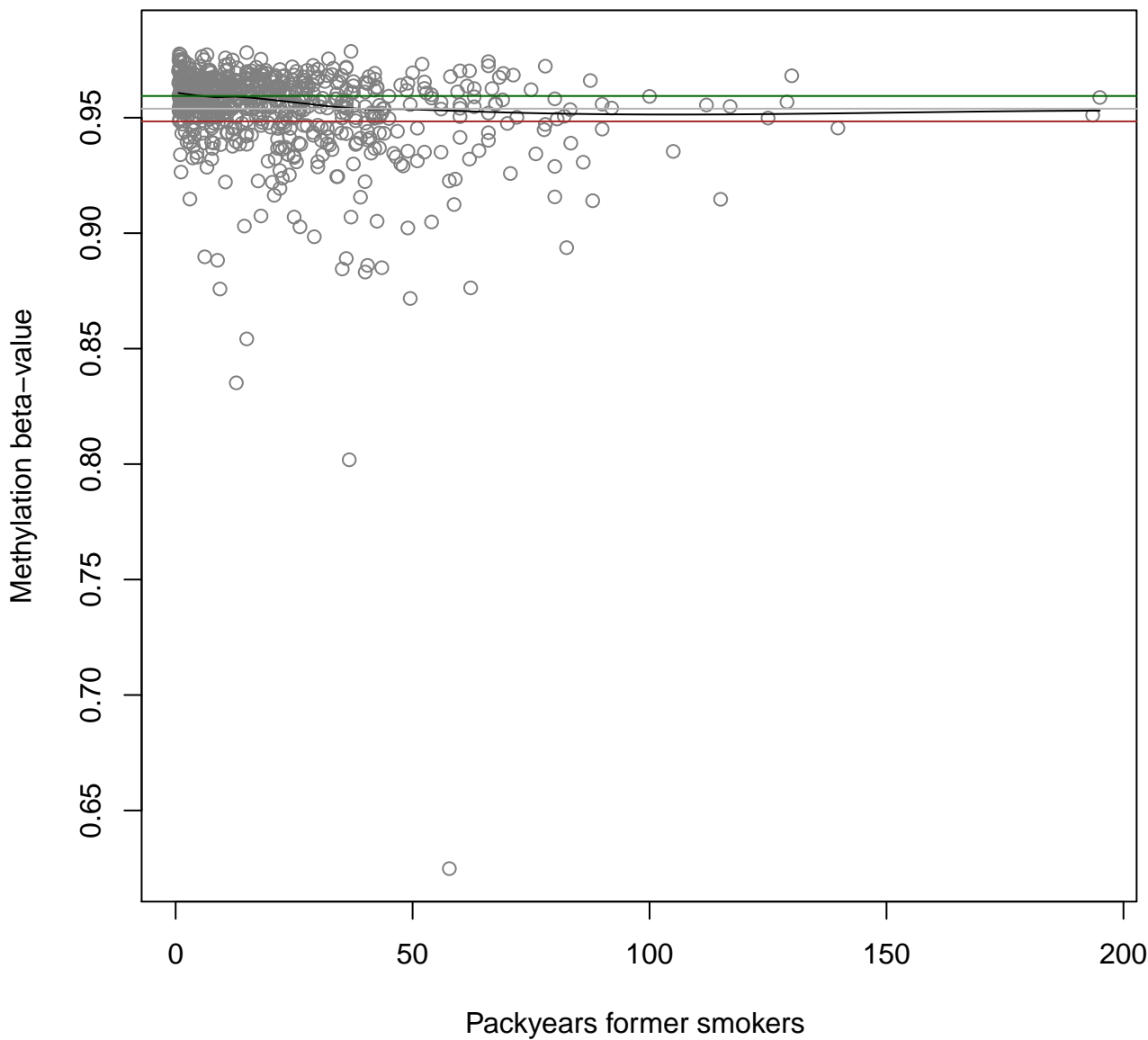

**cg23771366**

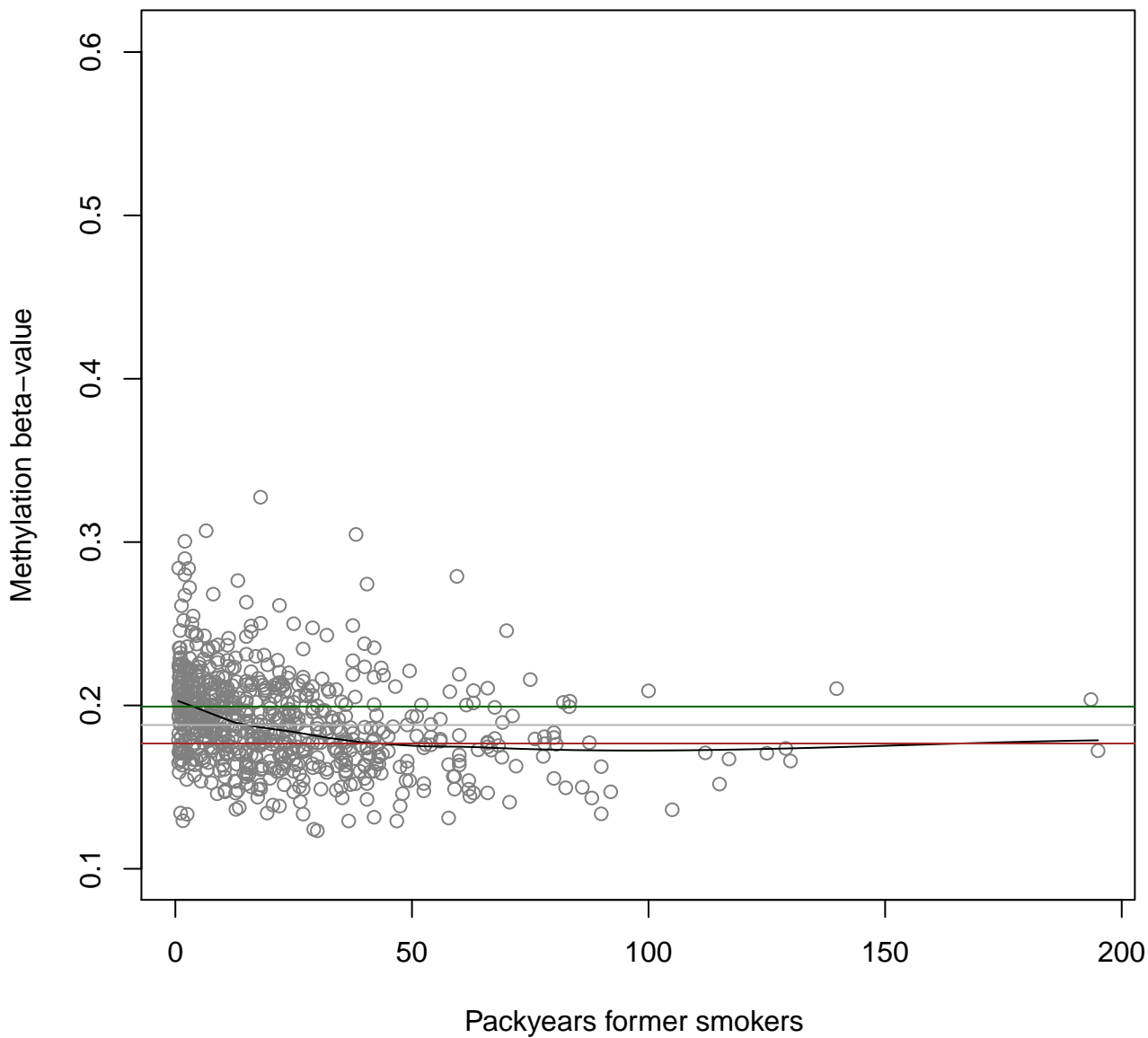

**cg25189904**

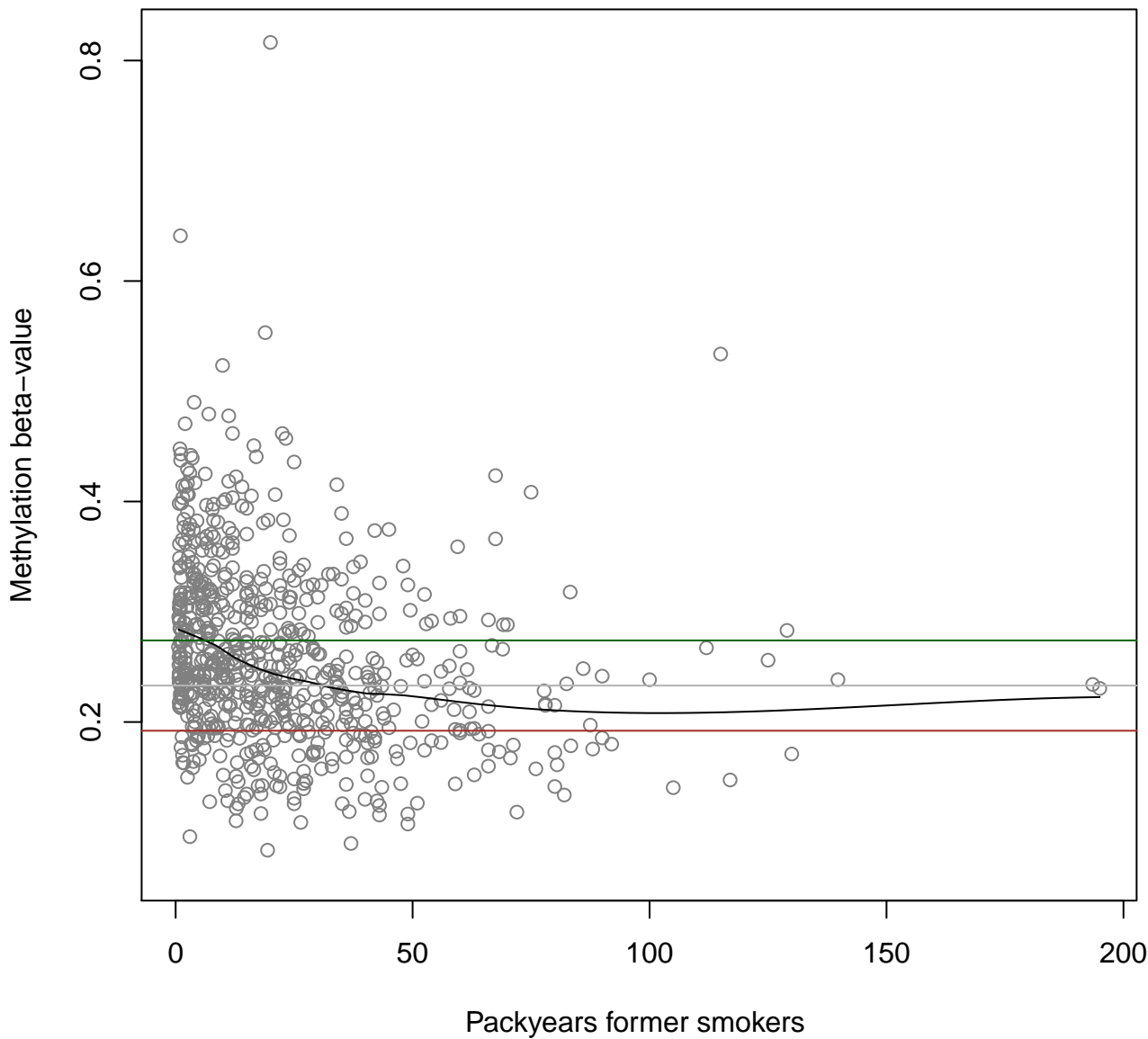

Supplement: Figure S3 — Influence of cumulative smoking exposure (pack-years) on the DNA methylation state in former smokers. The pack-years needed for former smokers to achieve a median ß-value methylation state at single CpG sites that is closer to or equals the one of current smokers is displayed by a loess curve in the scatterplots; the x-axis displays the number of pack-years, the y-axis displays the methylation level with the use of numbers between 0 (for 0% methylation) and 1 (for 100% methylation); horizontal brown line: median methylation of current smokers; horizontal green line: median methylation of never smokers; horizontal grey line: center line of current and never smokers median ß-value methylation; please see Table S6 for detailed data. (PDF) [file pone.0063812.s003.pdf]

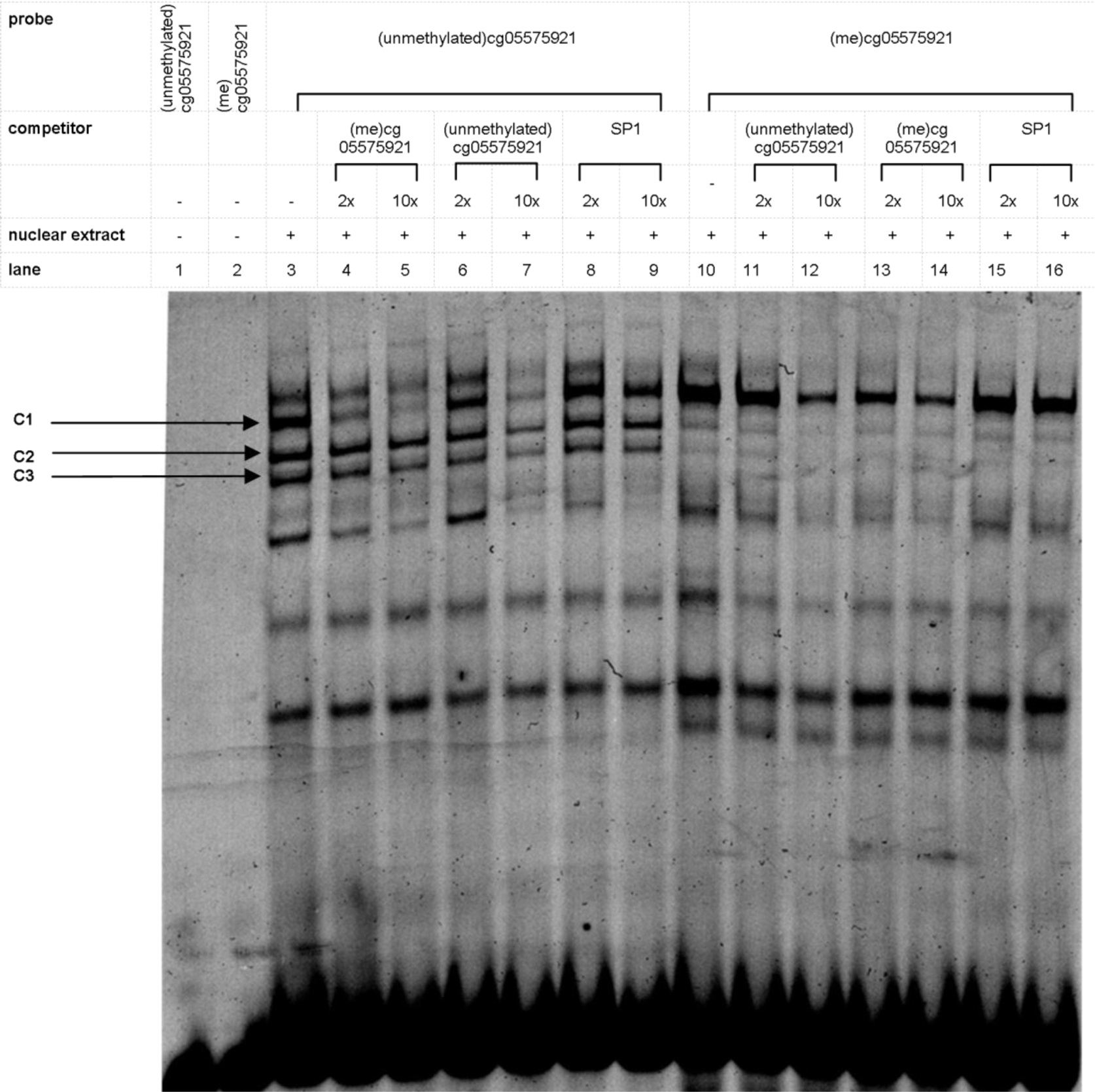

Supplement: Figure S4 — Methylation specific protein binding patterns of the CpG site cg05575921 in the AHRR gene. Methylated and unmethylated Cy5-labelled probes carrying the cg05575921 site were used in competition EMSAs using Raji and THP1 nuclear extracts. This figure shows one representative experiment of an EMSA using Raji nuclear extracts. Arrows indicate shifted protein-DNA complexes showing methylation specific binding patterns (C1–C3). In lane 1+2, free oligonucleotides without incubation with nuclear extracts are shown. Lane 3+10 show the results for EMSAs for the unmethylated and methylated variant without competition. In lane 4, 5, 11, 12 competitions with the unlabeled adverse oligonucleotides were performed, whereas competitions with the same unlabeled oligonucleotides were performed in lane 6, 7, 13, 14. To ensure specificity, competitions with unlabeled SP1-consensus oligonucleotides were performed in lane 8, 9, 15, 16. (me)cg: methylated c05575921, SP1 = Specificity protein 1. The experiment using THP1 nuclear extracts resulted in comparable methylation specific band patterns (data not shown). (TIF) [file pone.0063812.s004.tif]
