# Supplementary material for: Tobacco Smoking Leads to Extensive Genome-Wide Changes in DNA Methylation
Source: PLoS One. 2013 May 17;8(5):e63812. doi: 10.1371/journal.pone.0063812 (PMC3656907; doi:10.1371/journal.pone.0063812)
Supplement: Box S1 — Description of genes that correspond to CpG sites with a methylation difference of more than 5% in current vs. never smokers (in addition to AHRR and ALPP/ALPPL2 ). (PDF) [file pone.0063812.s014.pdf]

Box S1. Description of genes that correspond to CpG sites with a methylation difference of more than 5% in current vs. never smokers (in addition to AHRR and ALPP/ALPPL2)

HIVEP3: encodes for the transcription factor human immunodeficiency virus type I enhancer binding protein 3, which strongly inhibits TNF-alpha-induced NF-kappa-B activation and plays a critical role in inflammatory and apoptotic responses [2,3] as well as cell growth [3]. HIVEP3 is induced by T-cell receptor signaling and positively regulates the expression of IL2 in T-cells [6]. Furthermore, it is an essential regulator of adult bone formation [7].

GNG12: encodes for the guanine nucleotide binding protein (G protein), gamma 12. G proteins are involved as modulators or transducers in various transmembrane signaling systems and are composed of 3 units, alpha, beta and gamma [8]. The beta and gamma chains are required for the GTPase activity, for replacement of GDP by GTP, and for G protein-effector interaction. GNG12 might be a negative regulator of LPS-induced inflammation [9].

GFI1: encodes for the growth factor independent 1 transcription repressor and functions as a transcriptional repressor by controlling histone modifications that lead to silencing of the target gene promoters [10]. It plays a role in various developmental contexts [11], including lymphocyte development and activation [13], and is aberrantly expressed in lung tumors [14]. Gfi-1 plays a critical role both in enhancing Th2 cell expansion and in repressing induction of Th17 and CD103(+) iTreg cells [15].

***CACNA1D*:** encodes for calcium channel, voltage-dependent, L type, alpha 1D subunit, also known as Cav1.3. Voltage-gated Ca2+ channels divert Ca2+ signals to different cellular processes within different cell types, such as muscle contraction, neurotransmitter release, hormone secretion, gene expression, cell motility, cell division and cell death [18]. Cav1.3 can signal to transcriptional events and induce long lasting alterations of neuronal responsiveness [20,21]. It recently has been shown that CaV1.3 may play a crucial role in osmotic stress-induced Ca2+ influx and tight junction disruption in the intestinal epithelium [22].

TIAM2: encodes for T-cell lymphoma invasion and metastasis 2, which is a guanine nucleotide exchange factor that stimulates the GDP-GTP exchange activity of RHO-like GTPases and activates them. It connects extracellular signals to cytoskeletal activities. The encoded protein may play a role in neural cell development [23,24] and regulate cell migration by microtubule-mediated focal adhesion disassembly [25]. Recently it has been shown that the expression of TIAM2 promotes proliferation and invasion of liver cancer [26].

MYO1G: encodes for myosin 1G; is a plasma membrane-associated class I myosin, which is abundant in T and B lymphocytes and mast cells [27,28], and regulates cell elasticity [29].

…

…

CNTNAP2: encodes for contactin associated protein-like 2, a member of the neurexin family which functions in the vertebrate nervous system as cell adhesion molecule and receptor. CNTNAP2 has been associated with a wide spectrum of neuropsychiatric disorders such as developmental language and autism spectrum disorders, epilepsy and schizophrenia [1]. Furthermore, it undergoes aberrant methylation in pancreatic adenocarcinoma [4].

ZC3H3**:** encodes for zinc finger CCCH-type containing 3 and regulates mRNA nuclear adenylation and export [5].

LRP5: encodes for low density lipoprotein receptor-related protein 5, and binds and internalizes ligands in the process of receptor-mediated endocytosis. LRP5 plays a role in regulating bone mass [12], and development of lung microvessels and alveoli through the angiopoietin-Tie2 pathway [16]. It may play a role in smoke-induced bone loss [17] and contribute to the glucose-induced insulin secretion in the islets [19].

PCDH9: encodes for protocadherin 9. Protocadherins are a subfamily of cadherins, a large group of related glycoproteins that mediate calcium-dependent cell-to-cell adhesion via a homophilic mechanism. PCDH9 is localized to the cell membrane and expressed primarily in the brain, and is found in synaptic junctions, where it functions as a neuronal receptor involved in signal transduction and maintaining specific neuronal connections [30]. Expression of PCDH9 is found in hairy cell leukemia [31] and PCDH9 might function as a tumor suppressor during cancer development and progression [32]. PCDH9 might furthermore be a susceptibility locus for Rheumatoid arthritis [33].
Recently, a study characterized intra- and inter-individual methylomic variation across whole blood and multiple regions of the brain from multiple donors and found tissue-specific differentially methylated regions to be significantly enriched near genes involved in functional pathways related to neurodevelopment and neuronal differentiation, including PCDH9 [34].

RARA: encodes for retinoic acid receptor, alpha; which regulates the expression of target genes in a ligand-dependent manner and plays a role in acute promyelocytic leukaemia [35], germ cell development during spermatogenesis [36] and CD4+ T Cell Immunity and Homeostasis [37]. It plays an important role in cellular memory and imprinting by regulating the CpG methylation status of specific promoter regions [38].

LINGO3: encodes for leucine rich repeat and Ig domain containing 3, which is expressed in a broad but specific pattern in many tissues across the mouse embryo [39].

F2RL3: encodes for coagulation factor II (thrombin) receptor-like 3. The F2RL3 protein is relevant for cardiovascular physiology and plays a role in platelet activation [40] and cell signaling [41]. Breitling and co-workers reported an association of *F2RL3* methylation with mortality among patients with stable coronary heart disease [42].

References for Box S1.

1. Gregor A, Albrecht B, Bader I, Bijlsma EK, Ekici AB, et al. (2011) Expanding the clinical spectrum associated with defects in CNTNAP2 and NRXN1. BMC Med Genet 12: 106.
2. Oukka M, Kim ST, Lugo G, Sun J, Wu LC, et al. (2002) A mammalian homolog of Drosophila schnurri, KRC, regulates TNF receptor-driven responses and interacts with TRAF2. Mol Cell 9: 121-131.
3. Wu LC (2002) ZAS: C2H2 zinc finger proteins involved in growth and development. Gene Expr 10: 137-152.
4. Omura N, Li CP, Li A, Hong SM, Walter K, et al. (2008) Genome-wide profiling of methylated promoters in pancreatic adenocarcinoma. Cancer Biol Ther 7: 1146-1156.
5. Hurt JA, Obar RA, Zhai B, Farny NG, Gygi SP, et al. (2009) A conserved CCCH-type zinc finger protein regulates mRNA nuclear adenylation and export. J Cell Biol 185: 265-277.
6. Oukka M, Wein MN, Glimcher LH (2004) Schnurri-3 (KRC) interacts with c-Jun to regulate the IL-2 gene in T cells. J Exp Med 199: 15-24.
7. Jones DC, Wein MN, Glimcher LH (2007) Schnurri-3 is an essential regulator of osteoblast function and adult bone mass. Ann Rheum Dis 66 Suppl 3: iii49-51.
8. Hurowitz EH, Melnyk JM, Chen YJ, Kouros-Mehr H, Simon MI, et al. (2000) Genomic characterization of the human heterotrimeric G protein alpha, beta, and gamma subunit genes. DNA Res 7: 111-120.
9. Larson KC, Lipko M, Dabrowski M, Draper MP (2010) Gng12 is a novel negative regulator of LPS-induced inflammation in the microglial cell line BV-2. Inflamm Res 59: 15-22.
10. Duan Z, Zarebski A, Montoya-Durango D, Grimes HL, Horwitz M (2005) Gfi1 coordinates epigenetic repression of p21Cip/WAF1 by recruitment of histone lysine methyltransferase G9a and histone deacetylase 1. Mol Cell Biol 25: 10338-10351.
11. Moroy T (2005) The zinc finger transcription factor Growth factor independence 1 (Gfi1). Int J Biochem Cell Biol 37: 541-546.
12. Zhong Z, Williams BO (2012) Integration of cellular adhesion and Wnt signaling: Interactions between N-cadherin and LRP5 and their role in regulating bone mass. J Bone Miner Res 27: 1849-1851.
13. Moroy T, Khandanpour C (2011) Growth factor independence 1 (Gfi1) as a regulator of lymphocyte development and activation. Semin Immunol 23: 368-378.
14. Kazanjian A, Wallis D, Au N, Nigam R, Venken KJ, et al. (2004) Growth factor independence-1 is expressed in primary human neuroendocrine lung carcinomas and mediates the differentiation of murine pulmonary neuroendocrine cells. Cancer Res 64: 6874-6882.
15. Zhu J, Davidson TS, Wei G, Jankovic D, Cui K, et al. (2009) Down-regulation of Gfi-1 expression by TGF-beta is important for differentiation of Th17 and CD103+ inducible regulatory T cells. J Exp Med 206: 329-341.
16. Mammoto T, Chen J, Jiang E, Jiang A, Smith LE, et al. (2012) LRP5 regulates development of lung microvessels and alveoli through the angiopoietin-Tie2 pathway. PLoS One 7: e41596.
17. Ma D, Li Y, Hackfort B, Zhao Y, Xiao J, et al. (2012) Smoke-induced signal molecules in bone marrow cells from altered low-density lipoprotein receptor-related protein 5 mice. J Proteome Res 11: 3548-3560.
18. Yang SN, Berggren PO (2006) The role of voltage-gated calcium channels in pancreatic beta-cell physiology and pathophysiology. Endocr Rev 27: 621-676.
19. Fujino T, Asaba H, Kang MJ, Ikeda Y, Sone H, et al. (2003) Low-density lipoprotein receptor-related protein 5 (LRP5) is essential for normal cholesterol metabolism and glucose-induced insulin secretion. Proc Natl Acad Sci U S A 100: 229-234.
20. Deisseroth K, Mermelstein PG, Xia H, Tsien RW (2003) Signaling from synapse to nucleus: the logic behind the mechanisms. Curr Opin Neurobiol 13: 354-365.
21. Barbado M, Fablet K, Ronjat M, De Waard M (2009) Gene regulation by voltage-dependent calcium channels. Biochim Biophys Acta 1793: 1096-1104.
22. Samak G, Narayanan D, Jaggar JH, Rao R (2011) CaV1.3 channels and intracellular calcium mediate osmotic stress-induced N-terminal c-Jun kinase activation and disruption of tight junctions in Caco-2 CELL MONOLAYERS. J Biol Chem 286: 30232-30243.
23. Yoshizawa M, Hoshino M, Sone M, Nabeshima Y (2002) Expression of stef, an activator of Rac1, correlates with the stages of neuronal morphological development in the mouse brain. Mech Dev 113: 65-68.
24. Matsuo N, Hoshino M, Yoshizawa M, Nabeshima Y (2002) Characterization of STEF, a guanine nucleotide exchange factor for Rac1, required for neurite growth. J Biol Chem 277: 2860-2868.
25. Rooney C, White G, Nazgiewicz A, Woodcock SA, Anderson KI, et al. (2010) The Rac activator STEF (Tiam2) regulates cell migration by microtubule-mediated focal adhesion disassembly. EMBO Rep 11: 292-298.
26. Chen JS, Su IJ, Leu YW, Young KC, Sun HS (2012) Expression of T-cell lymphoma invasion and metastasis 2 (TIAM2) promotes proliferation and invasion of liver cancer. Int J Cancer 130: 1302-1313.
27. Patino-Lopez G, Aravind L, Dong X, Kruhlak MJ, Ostap EM, et al. (2010) Myosin 1G is an abundant class I myosin in lymphocytes whose localization at the plasma membrane depends on its ancient divergent pleckstrin homology (PH) domain (Myo1PH). J Biol Chem 285: 8675-8686.
28. Pierce RA, Field ED, Mutis T, Golovina TN, Von Kap-Herr C, et al. (2001) The HA-2 minor histocompatibility antigen is derived from a diallelic gene encoding a novel human class I myosin protein. J Immunol 167: 3223-3230.
29. Olety B, Walte M, Honnert U, Schillers H, Bahler M (2010) Myosin 1G (Myo1G) is a haematopoietic specific myosin that localises to the plasma membrane and regulates cell elasticity. FEBS Lett 584: 493-499.
30. Asahina H, Masuba A, Hirano S, Yuri K (2012) Distribution of protocadherin 9 protein in the developing mouse nervous system. Neuroscience 225C: 88-104.
31. Basso K, Liso A, Tiacci E, Benedetti R, Pulsoni A, et al. (2004) Gene expression profiling of hairy cell leukemia reveals a phenotype related to memory B cells with altered expression of chemokine and adhesion receptors. J Exp Med 199: 59-68.
32. Wang C, Yu G, Liu J, Wang J, Zhang Y, et al. (2012) Downregulation of PCDH9 predicts prognosis for patients with glioma. J Clin Neurosci 19: 541-545.
33. Wei Z, Li M (2007) Genome-wide linkage and association analysis of rheumatoid arthritis in a Canadian population. BMC Proc 1 Suppl 1: S19.
34. Davies MN, Volta M, Pidsley R, Lunnon K, Dixit A, et al. (2012) Functional annotation of the human brain methylome identifies tissue-specific epigenetic variation across brain and blood. Genome Biol 13: R43.
35. Wells RA, Catzavelos C, Kamel-Reid S (1997) Fusion of retinoic acid receptor alpha to NuMA, the nuclear mitotic apparatus protein, by a variant translocation in acute promyelocytic leukaemia. Nat Genet 17: 109-113.
36. Doyle TJ, Braun KW, McLean DJ, Wright RW, Griswold MD, et al. (2007) Potential functions of retinoic acid receptor A in Sertoli cells and germ cells during spermatogenesis. Ann N Y Acad Sci 1120: 114-130.
37. Hall JA, Cannons JL, Grainger JR, Dos Santos LM, Hand TW, et al. (2011) Essential role for retinoic acid in the promotion of CD4(+) T cell effector responses via retinoic acid receptor alpha. Immunity 34: 435-447.
38. Laursen KB, Wong PM, Gudas LJ (2012) Epigenetic regulation by RARalpha maintains ligand-independent transcriptional activity. Nucleic Acids Res 40: 102-115.
39. Haines BP, Rigby PW (2008) Expression of the Lingo/LERN gene family during mouse embryogenesis. Gene Expr Patterns 8: 79-86.
40. Kahn ML, Nakanishi-Matsui M, Shapiro MJ, Ishihara H, Coughlin SR (1999) Protease-activated receptors 1 and 4 mediate activation of human platelets by thrombin. J Clin Invest 103: 879-887.
41. Chen HT, Tsou HK, Tsai CH, Kuo CC, Chiang YK, et al. (2010) Thrombin enhanced migration and MMPs expression of human chondrosarcoma cells involves PAR receptor signaling pathway. J Cell Physiol 223: 737-745.
42. Breitling LP, Salzmann K, Rothenbacher D, Burwinkel B, Brenner H (2012) Smoking, F2RL3 methylation, and prognosis in stable coronary heart disease. Eur Heart J.
